# Tumorous IRE1α facilitates CD8^+^T cells-dependent anti-tumor immunity and improves immunotherapy efficacy in melanoma

**DOI:** 10.1186/s12964-024-01470-8

**Published:** 2024-01-30

**Authors:** Yuqi Yang, Sijia Wang, Xiang-xu Wang, Sen Guo, Huina Wang, Qiong Shi, Yangzi Tian, Hao Wang, Tao Zhao, Hengxiang Zhang, Baolu Zhang, Tianwen Gao, Chunying Li, Xiuli Yi, Weinan Guo

**Affiliations:** 1 Department of Dermatology, Xijing Hospital, Fourth Military Medical University, Xi’an, Shaanxi China; 2 Department of Dermatology, Nanfang Hospital, Southern Medical University, Guangzhou, Guangdong China; 3 Department of Clinical Oncology, Xijing Hospital, Fourth Military Medical University, Xi’an, Shaanxi China

**Keywords:** ER stress, Melanoma, Immunosurveillance, Immunotherapy, Anti-PD-1 antibody

## Abstract

**Background:**

Tumor cells frequently suffer from endoplasmic reticulum (ER) stress. Previous studies have extensively elucidated the role of tumorous unfolded protein response in melanoma cells, whereas the effect on tumor immunology and the underlying mechanism remain elusive.

**Methods:**

Bioinformatics, biochemical assays and pre-clinical mice model were employed to demonstrate the role of tumorous inositol-requiring transmembrane kinase/endoribonuclease 1α (IRE1α) in anti-tumor immunity and the underlying mechanism.

**Results:**

We firstly found that IRE1α signaling activation was positively associated with the feature of tumor-infiltrating lymphocytes. Then, pharmacological ER stress induction by HA15 exerted prominent anti-tumor effect in immunocompetent mice and was highly dependent on CD8^+^T cells, paralleled with the reshape of immune cells in tumor microenvironment via tumorous IRE1α-XBP1 signal. Subsequently, tumorous IRE1α facilitated the expression and secretion of multiple chemokines and cytokines via XBP1-NF-κB axis, leading to increased infiltration and anti-tumor capacity of CD8^+^T cells. Ultimately, pharmacological induction of tumorous ER stress by HA15 brought potentiated therapeutic effect along with anti-PD-1 antibody on melanoma in vivo.

**Conclusions:**

Tumorous IRE1α facilitates CD8^+^T cells-dependent anti-tumor immunity and improves immunotherapy efficacy by regulating chemokines and cytokines via XBP1-NF-κB axis. The combination of ER stress inducer and anti-PD-1 antibody could be promising for increasing the efficacy of melanoma immunotherapy.

**Supplementary Information:**

The online version contains supplementary material available at 10.1186/s12964-024-01470-8.

## Background

Melanoma is the most lethal skin cancer originated from the malignant transformation of epidermal melanocytes [1]. The incidence of melanoma is gradually increasing in the past few years, whereas the prognosis of patients remains unoptimistic even though the innovative proceedings of targeted therapy and immunotherapy [2]. Recently, investigations regarding melanoma pathogenesis begin to emphasize more on tumor microenvironment (TME). TME is composed of mixed populations of cells residing within dynamic micro-environmental niche, in which melanoma cells coexist with surrounding immune cells in extracellular matrix [3, 4]. Immune checkpoint molecules expressed by melanoma cells like PD-L1 contributes to the impairment of the function of infiltrating CD8^+^T cells and immune evasion [5], which provides the molecular basis of anti-PD-1/PD-L1 immunotherapy and have gained unprecedented progress in improving the outcome of patients [6]. Of note, tumor cells can employ multiple approaches to re-shape the profile of immune cells in TME and alter the characteristic of anti-tumor immunity. To be specific, mutant BRAF and MEK in melanoma cells regulate the infiltration and function of T cells and dendritic cells in TME via GSDME-dependent pyroptosis and HMGB1 secretion [7]. In addition, epigenetic enzyme KDM5B in melanoma cells recruits the H3K9 methyl-transferase SETDB1 to repress endogenous retroelements and subsequent type-I interferon response, so as to suppress anti-tumor immunity [8]. Therefore, to target specific characteristics and pathways in melanoma cells and to intervene the interplay between tumor cells and immune cells are of great potential for invigorating anti-tumor immunity. To forwardly elucidate how specific tumorous characteristics regulate immune cells in TME can help to provide more druggable targets for melanoma immunotherapy.

The endoplasmic reticulum (ER) is a critical intracellular organelle that is mainly responsible for protein synthesis, folding and modification in cells [9]. Multiple exogenous and cell-intrinsic events can impede the protein-folding capacity of ER, which leads to excessive accumulation of unfolded and misfolded proteins, and subsequently provokes a state called ER stress. In response, unfolded protein response would be activated to mediate the removal or recycling of misfolded protein and reinstate ER homeostasis [10]. It has been well documented that melanomas commonly endure persistent ER stress [11], which is a hallmark of tumor cells as is revealed by the up-regulation of molecules implicated in unfolded protein response (UPR) pathway. Some intrinsic cellular alterations including high transcription and translation rates, metabolic reprogramming and ROS over-production can promote the generation of misfolded proteins [12]. Moreover, solid tumors often endow micro-environmental conditions like nutrient deprivation, acidosis and hypoxia, all of which also prominently induce ER stress [13–16]. As a consequence, the activation of UPR machinery greatly contributes to tumor cell proliferation and metastasis, and mediates the resistance to current available therapies [17, 18], indicating that the critical pathogenic role of UPR in melanoma. By contrast, prolonged unresolved or extreme ER stress would exert lethal effect on tumor cells, and some therapeutic agents triggering ER stress have been proved of great potential for melanoma treatment [19, 20]. These studies indicate that to precisely interfere ER stress and UPR in a context-dependent manner is a potential strategy for controlling melanoma progression. Of note, previous studies have mainly demonstrated the effect of tumorous UPR on the intrinsic malignant behaviors of melanoma cells [11, 17]. However, whether tumorous UPR can modulate tumor immune microenvironment remains far from understood and needs further investigation. Recently, HA15 has been well proved as a potent and specific inhibitor of ER chaperone BiP/GRP78/HSPA5 that can prominently trigger ER stress via the suppression of ATPase activity of BiP [19]. While it exerts robust anti-tumor effect through the induction of ER stress, whether it can subsequently impact tumor immunology remains elusive.

In the present study, we firstly found that the activation of tumorous IRE1α was positively associated with the feature of tumor-infiltrating lymphocytes in TCGA SKCM database and melanoma tissues. Then, it was uncovered that pharmacological induction of ER stress by HA15 exerted better anti-tumor effect in immunocompetent mice and was highly dependent on CD8^+^T cells, paralleled with the reshape of immune cells in tumor microenvironment. This effect was dependent on tumorous IRE1α-XBP1 axis. Subsequently, the mechanism underlying the role of tumorous IRE1α in anti-tumor immunity was elucidated, with particular focus on the regulation of chemokines and cytokines. Ultimately, the effect of the combination of ER stress inducer and anti-PD-1 antibody was testified in pre-clinical mice model.

## Materials and methods

### Cell culture and reagents

Human melanoma cell lines A2058 and A375, and mouse melanoma cell line B16F10 were purchased from the American Type Culture Collection (ATCC). All cultured cell lines were grown in Dulbecco’s modified Eagle’s medium (DMEM) (Gibco, USA) supplemented with 10% fetal bovine serum (Gibco), 100 units/mL penicillin and 100 mg/mL streptomycin at 37 °C and 5% CO_2_. All melanoma cell lines were authenticated by short-tandem repeat (STR) fingerprinting in center of DNA typing in Fourth Military Medical University and tested for mycoplasma contamination. The ER stress inducer thapsigargin (TG) (HY-13,433, MCE, South Brunswick, USA) were dissolved in DMSO and used at the concentration of 0.5 µM for A2058 cell line, 0.2 µM for A375 cell line and 0.2 µM for B16F10 cell line. For in vitro experiment, HA15 (HY-100,437, MCE, South Brunswick, USA) were dissolved in DMSO and used at the concentration of 10 µM for A2058, A375 and B16F10 cell lines. For in vivo experiment, HA15 (HY-100,437, MCE, South Brunswick, USA) was dissolved in the clarified mixture of 10% DMSO and 90% corn oil (HY-Y1888, MCE, South Brunswick, USA) and used at the concentration of 0.7 mg/ 100 µl/ d/ mice. The IRE1α specific inhibitor STF-083010 (HY-15,845, MCE, South Brunswick, USA) was dissolved in DMSO and used at the concentration of 10 µM for A2058, A375 and B16F10 cell lines. The IRE1α specific inhibitor MKC8866 (HY-15,845, MCE, South Brunswick, USA) was dissolved in DMSO and used at the concentration of 0.5 µM for A2058, A375 and B16F10 cell lines. The NF-κB inhibitor BAY-11-7085 (HY-10,257, MCE, South Brunswick, USA) was dissolved in DMSO and used at the concentration of 1 µM for A2058, A375 and B16F10 cell lines.

### Western blot

 The protein extracts were lysed with RIPA buffer (P0013C, Beyotime Biotechnology) containing protease and phosphatase Inhibitor Cocktail (1:100, P002, NCM biotech) on ice for 30 min and were then centrifuged at 12,000 g for 15 mins. The supernatant was collected was quantified with a BCA Protein Assay Kit (P0012, Beyotime Biotechnology). Equal amounts of protein samples were separated by SDS-PAGE (P0690, Beyotime Biotechnology) and were then transferred to Immobilon-P PVDF membrane (IPVH00010, MilliporeSigma, Burlington, MA). Spectra Multicolor Broad Range Protein Ladder (26,617, Fermentas, Waltham, MA) was used to confirm protein electrophoresis and transferring. Then the membranes were blocked for 1 h with blocking solution (5% nonfat milk in 1× Tris buffered saline containing 0.1% Tween 20 (TBST) and incubated with primary antibodies overnight at 4 °C. After washing 3 times with 1 × TBST, the membranes were incubated with horseradish peroxidase-conjugated goat anti-mouse (1:5000, 115-035-003, Jackson ImmunoResearch Laboratories, West Grove, PA) or anti-rabbit IgG (1:5000, 111-035-003, Jackson ImmunoResearch Laboratories, West Grove, PA) for 1 h at room temperature, and the signals were detected with Pierce Fast Western Blot Kit, ECL Substrate (35,055, Thermo Fisher Scientific). GAPDH was used as a loading control. The dilutions of the primary antibodies used for immunoblotting were listed in Additional file 2: Table 2.

### RNA extraction and quantitative real-time PCR

 Total RNA was extracted from cultured cells using TRIzol reagent (15,596,018, Invitrogen, Carlsbad, CA) and then reversely transcribed to cDNA using the PrimeScript RT Master Mix kit (RR036A, TaKaRa, Maebashi, Japan) according to the manufacturer’s instructions. qRT-PCR was performed using SYBR Premix Ex TaqTM II kit (RR820A, TaKaRa) with Bio-Rad Multicolor Real-time PCR Detection System (iQTM5, Bio-Rad Laboratories, Hercules, CA). GAPDH mRNA was used as an internal control and threshold cycle values were used to calculate the relative mRNA expression level by the 2^−ΔΔCt^ method. The primer sequences used for qRT-PCR were listed in Additional file 2: Table 3.

### ELISA assay

ELISA analysis on culture medium of melanoma cells or the blood serum of mice after indicated treatment was performed using the Human IL-6 ELISA Kit (Neobioscience, EHC007.96), Human TNF-α ELISA Kit (Neobioscience, EHC103a.96), Human MIG/CXCL9 ELISA Kit (Neobioscience, EHC114.96), Human IP-10/CXCL10 ELISA Kit (Neobioscience, EHC157.96), Human I-TAC/CXCL11 ELISA Kit (Neobioscience, EHC084.96), Mouse TNF-α ELISA Kit (Elabscience, E-MSEL-M0002), Mouse IL-2 ELISA Kit (Elabscience, E-MSEL-M0036), Mouse IL-6 ELISA Kit (Elabscience, E-EL-M0044c), Mouse ELISA Kit MIG/CXCL9 ELISA Kit (Elabscience, E-EL-M3077), Mouse IP-10/CXCL10 ELISA Kit (Elabscience, E-EL-M0021c), Mouse I-TAC/CXCL11 ELISA Kit (Elabscience, E-EL-M0056c) and Mouse HMGB-1 ELISA Kit (Elabscience, E-EL-M0676c) according to the manufacturer’s instructions. The absorbance (A450 nm) was measured with a plate reader (Bio-Rad). The ELISA result was normalized by cell counting of the attached live cells after harvesting the supernatants.

### In vitro analysis of cell surface PD-L1 staining by flow cytometry

Melanoma cells with indicated treatment were washed twice with PBS, harvested using trypsin digestion, and further washed twice in FACS buffer by centrifugation. Cells were incubated with PE-conjugated anti-human CD274 (Biolegend, #329706) in FACS buffer for 30 min at 4 °C, subsequently washed once and resuspended in FACS buffer for PD-L1-positive cells detection by flow cytometry using on a BD LSR Fortessa Cell analyzer (BD Bioscience). Data were analyzed using FlowJo software. The mean fluorescence intensity (MFI) of PD-L1 relative to isotype control was calculated as the mean fluorescence of PD-L1-positive cells by using FlowJo V10 software (Treestar Inc).

### T cell-mediated tumor cell killing assay

The co-culture system for T cell and tumor cell was established as described previously [21]. To acquire activated T cells, human peripheral blood mononuclear cells (PBMCs) were isolated from whole blood of healthy donors and cultured in RPMI 1640 medium with ImmunoCult Human CD3/CD28/CD2 T cell activator (10,970; STEMCELL Technologies) and Recombinant Human IL-2 (1000 U/mL, 202-1 L-050, R&D) for 5 days according to the manufacturer’s protocol. The A2058 and A375 melanoma cells which were treated with HA-15 (10 µM) for 24 h after pretreated with or without IRE1α specific inhibitor STF-083010 (10 µM), MKC8866 (0.5 µM) or NF-κB inhibitor BAY-11-7085 (1 µM) for 24 h were allowed to adhere to the plates overnight and then incubated for 24 h with activated PBMC cells at the ratio of 1:3 in the presence of 100 ng/ml anti-CD3ε mAb (16–0037; eBioscience) and 1000 U/mL IL-2 (202-1 L-050, R&D). The living cancer cells were left to quantify by a spectrometer at OD (570 nm) followed by crystal violet staining. T cells were collected and assessed by flow cytometry. Zombie dye (Biolegend, #423108) was used for live/dead cell determination. For surface staining, cells were washed and stained with APC anti-human CD3 antibody (Biolegend, #300312), PE-cy7 anti-human CD8α antibody (Biolegend, #344750) and FITC anti-human CD69 antibody (Biolegend, #310904). For Granzyme B and IFN-γ staining, cells were fixed and permeabilized by Transcription Factor Buffer Set (Biolegend, # 424401), and then stained with PE- anti-human/ mouse Granzyme B antibody (Biolegend, #372208) and Pacific Blue anti-human IFN-γ antibody (Biolegend, #502522) according to the manufactures’ instructions. Stained cells were runed on BD LSR Fortessa Cell analyzer (BD Bioscience), and all data were analyzed using FlowJo v10 software (Treestar Inc) and GraphPad Prism 9.0 (GraphPad Software).

### Animal models

All NOD-SCID nude mice and C57BL/6 mice used in the experiments were female and approximately 6 weeks old. Mice health status was checked by following the protocols and The University Committee on Use and Care of Animals of Fourth Military Medical University approved all animal protocols used in this study. For in vivo studies, 5 × 10^5^ B16F10 melanoma cells, B16F10-sh-mNC1 melanoma cells, B16F10-sh-mIRE1α melanoma cells, B16F10-sh-mNC2 melanoma cells or B16F10-sh-mXBP1 melanoma cells were injected subcutaneously into the right posterior flanks of nude mice or C57BL/6 mice (Laboratory Animal Resources, Fourth Military Medical University, Shaanxi, China). The mice were randomly divided into the indicated groups for each experiment and monitored for the development of tumors by measurements of mice weight, tumor length (L), and tumor width (W). The tumor volume ([L × W^2^] × 0.5) was calculated based on caliper measurements every three day after injection. At the end of the experiment, mice were sacrificed by cervical dislocation. The tumor issues were excised, weighed, photographed and harvested for further tissue analyses.

For ER stress inducer HA15 treatments, mice were treated with HA15 (0.7 mg/mice) (MCE, HY-100,437) or corn oil (MCE, HY-Y1888) was intraperitoneally injected once every day for 9 days beginning on day 7 after the subcutaneous 5 × 10^5^ B16F10 melanoma cells inoculation. For antibody treatments, mice were treated with 100 µg of anti-PD-1 (BioXCell, BE0146) or an IgG isotype control (BioXCell, BE0083) per mouse via intraperitoneal injection every three days for 9 days beginning on day 7 after 5 × 10^5^ B16F10 melanoma cells were subcutaneously implanted. For combination treatment, mice were treated with HA15 (0.7 mg/ mice/ day), PD-1 antibodies (100 µg/ mice/ three days) or combination via intraperitoneal injection for 9 days beginning on day 7 after B16F10 cells were subcutaneously implanted. Prior to treatments initiation, mice were randomized into treatment or control groups with similar average tumor volumes.

### In vivo depletion of CD8^+^T cells

To deplete CD8^+^T cells in vivo, mice were intraperitoneally injected with 100 µg of anti-CD8α antibody (BioXCell, BE0117) per mice 2 days and 1 day before tumor inoculation and every 2 days thereafter to ensure sustained depletion of CD8^+^ T cell subset during the experimental period as previously described [22]. One group of mice treated with IgG isotype (BioXCell, BE0083) served as controls.

### Immunofluorescence staining analysis

Mice received 5 × 10^5^ murine melanoma cells, and each treatment was indicated as described above. The mice were euthanized and sacrificed at the end point, tumors were harvested and mounted in OCT embedding compound (Tissue Tek, Sakura) for frozen tissue sections or fixed in 4% paraformaldehyde for paraffin-embedded tissue sections. Four micrometer sections were cut using a cryostat and slides containing cryostat sections were stored at -80 °C for immunofluorescence staining analysis of frozen tissue sections. Five micrometer sections were cut and stored at room temperature (RT) for immunofluorescence staining analysis of paraffin-embedded tissue sections.

For frozen tissue sections, sections were blocked with 10% goat serum in PBS for 1 h at room temperature and then stained overnight at 4 °C with Rat mAb to CD8α (1:200, ab22378, abcam) antibody. After washed with PBS next day, goat anti-rat IgG H&L (FITC, 1:100, EK041, Zhuangzhibio, Xi’an, China) secondary antibody in PBS were used for 1 h at room temperature and DAPI (1:1000, Roche, Basel, Switzerland) was incubated for nuclear staining for 15 min. Sections were washed with PBS and obtained with a Zeiss LSM 800 confocal laser scanning microscope. Images were taken with ZEN Pro 2012 (Zeiss) software and processed with Fiji software.

For paraffin-embedded tissue sections, sections were deparaffinized and rehydrated with graded ethanol dilutions. Heat-mediated antigen retrieval with Tris-EDTA buffer (pH 9.0) was performed, the sections were blocked with 10% goat serum in PBS for 1 h at room temperature and then stained overnight at 4 °C with rabbit pAb to XBP1s (1:100, 24868-1-AP, proteintech), rabbit pAb to Annexin A1 (1:50, 21990-1-AP, proteintech), rabbit pAb to Calreticulin (1:50, 27298-1-AP, proteintech), rabbit pAb to HMGB1 (1:50, 10829-1-AP, proteintech), rabbit mAb to p-MLKL (Ser345) (1:500, 37,333, CST), mouse mAb to Foxp3 (1:50, 65089-1-Ig, proteintech), CoraLite® Plus 488 Rat mAb to CD4 (1:50, CL488-65141, proteintech), rabbit pAb to Granzyme B (1:50, 13588-1-AP, proteintech), rabbit pAb to IFN-γ (1:50, 105,995-T08, Sinobiological) or rat mAb to CD8α (1:50, 65069-1-Ig, proteintech) antibody. The remaining steps were the same as that for immunofluorescence staining of frozen tissue sections except the goat anti-rabbit IgG H&L (Cy3, 1:100, EK022, Zhuangzhibio, Xi’an, China), the goat anti-rabbit IgG H&L (FITC, 1:100, EK023, Zhuangzhibio, Xi’an, China), the goat anti-mouse IgG H&L (Cy3, 1:100, EK012, Zhuangzhibio, Xi’an, China) or the goat anti-rat IgG H&L (FITC, 1:100, EK041, Zhuangzhibio, Xi’an, China) secondary antibody were used.

### Analysis of tumor-infiltrating immune cells by flow cytometry

General, tumor samples were isolated from the mice with indicated treatment after the mice was sacrificed and grinded mechanically using a syringe plunger and filtered with a 70 µM filter filtration (FALCON cell strainer, Corning, NY, USA). The isolated single-cell suspensions from tumors were cultured in RPMI 1640 medium containing 10% FBS with or without activation by cell activation cocktail with Brefeldin A (Biolegend, #423304) for 5 h at 37 °C, then assessed by flow cytometry. Samples prepared as described above were blocked with anti-mouse Fc blocking Ab (Biolegend, #101320) to reduce nonspecific immunofluorescent staining at room temperature (RT) for 15 min, and the dead and living cells were stained with Zombie UV™ Fixable Viability Kit (Biolegend, #423108) at RT for 15 min. For surface staining, cells were washed and then stained with an isotype or the antibodies listed in Additional file 2: Table 2 for 30 min at 4 °C. For intracellular staining, cells were fixed and permeabilized using True-Nuclear™ Transcription Factor Buffer Set (Biolegend, #424401), then stained with antibodies for intracellular staining listed in Additional file 2: Table 2 according to the manufactures’ instructions. After 30 min incubation at 4 °C, cells were washed twice with FACS buffer and were fixed with 1% Formaldehyde overnight at 4 °C for next analysis. Flow cytometry was performed on BD LSR Fortessa Cell analyzer (BD Bioscience) and BD FACS Diva Software version 7, and data were analyzed using FlowJo v10 software (Treestar Inc) and GraphPad Prism V.9.1.1 (GraphPad Software). All samples were analyzed by gating on viable cells followed by exclusion of duplets. All results show fluorescence on a biexponential scale.

### Statistical analysis

Statistical analysis was performed using GraphPad Prism 9 software (GraphPad Software, San Diego, CA). When normally distributed according to one of these tests, two-tailed Student’s t test was applied to determine the significance of differences between two groups of independent samples, while one-way ANOVA or two-way ANOVA were used to analyze the differences among multiple groups. Pearson’s correlation analysis was performed to determine the correlation between two variables. All p values are indicated in the figure (^*^p < 0.05; ^**^p < 0.01; ^***^p < 0.001; ns, not significant).

## Results

### IRE1α activation is highly correlated with the feature of tumor-infiltrating lymphocytes in melanoma

**Fig. 1:**
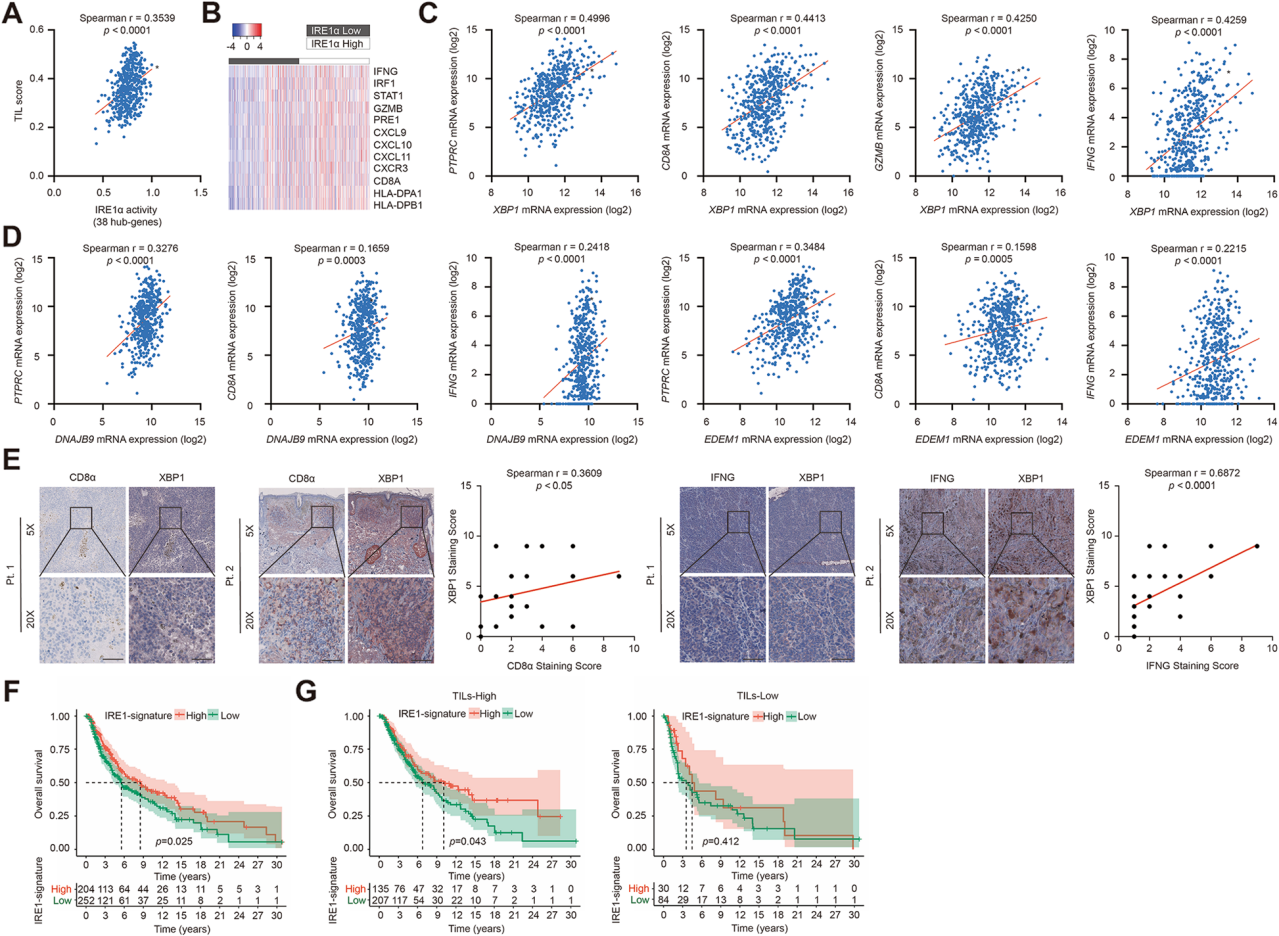
IRE1α signature score is highly correlated with the feature of tumor-infiltrating lymphocytes in melanoma. **A** Correlation analysis of 38-hub genes representative of the IRE1α signature with tumor-infiltrating lymphocytes score in TCGA SKCM database. **B** The mRNA expression of Th1-, cytotoxic mechanisms-, chemokines-, T cell, and MHC class I and II genes based on the groups defined relative to IRE1α activity (High or Low) in TCGA SKCM database. **C** Correlation analysis of *XBP1* with *PTPRC*, *CD8A*, *GZMB* and *IFN-γ* expression in TCGA SKCM database. **D** Correlation analysis of *DNAJB9* and *EDEM1* with *PTPRC*, *CD8A* and *IFN-γ* expression in TCGA SKCM database. **E** Immunohistochemical staining and correlation analysis of XBP1, CD8α and IFN-γ in a cohort of 31 melanoma tissues. Pt.1, Patient 1; Pt.2, Patient 2. Scale bar = 50 μm. **F** The overall survival of melanoma patients based on the groups defined relative to IRE1α activity (High or Low) in TCGA SKCM database. **G** The overall survival of melanoma patients with high TIL score (hot tumor) or low TIL score (cold tumor) based on the groups defined relative to IRE1α activity (High or Low) in TCGA SKCM database. *r* value was calculated by Spearman correlation. *p* value was calculated by two tailed Student’s *t*-test

 In order to elucidate whether ER stress regulates tumor immunology in melanoma, we turned to TCGA skin cutaneous melanoma (SKCM) database to figure out the relationship between three UPR branches and tumor-infiltrating lymphocytes score. While neither the expression of IRE1α, ATF6 nor PERK (encoded by *ERN1*, *ATF6* and *EIF2AK3* respectively) was not strongly associated with tumor-infiltrating lymphocytes (TIL) score (Additional file 1: Fig. S1A), the activity of IRE1α, reflected by a cluster of gene sets demonstrated in a previous report [23], was prominently correlated with TIL score in melanoma (Fig. 1A). In particular, a IRE1-dependent gene expression signature in U87 cells utilizing IRE1 dominant-negative-expressing cells has been previously discerned. The gene expression signature then underwent processing through the Bioinfominer pipeline to enhance its functional relevance with IRE1, resulting in the identification of 38 IRE1 signaling hub genes. This 38-gene signature was subsequently compared with the transcriptomes of the GBM TCGA (Cancer Genome Atlas Research Network, 2008) and GBMmark (in-house) cohorts. The heatmap displayed that melanomas with high IRE1α activity have increased expressions of TIL signature molecules like *IFNG*, *GZMB*, *CD8A* and *CXCL10* (Fig. 1B). Moreover, the expression of the critical downstream mediator of IRE1α pathway, *XBP1*, was also highly associated with the mRNA levels of the indicators of lymphocytes and anti-tumor immunity, including *PTPRC* (encoding CD45), *CD8A*, *GZMB* and *IFNG* (Fig. 1C). Moreover, the mRNA expressions of two critical XBP1 target genes *ERDJ4* and *EDEM1* were also significantly correlated with *PTPRC*, *CD8A* and *IFNG*, respectively (Fig. 1D), indicating the potential facilitative role of IRE1α-dependent UPR branch in anti-tumor immunity in melanoma. Besides, we also employed immunohistochemical staining analysis in a cohort of 31 melanoma tissues (Additional file 2: Table 1), which revealed that the staining scores of XBP1 were highly correlated with those of CD8α and IFN-γ, respectively (Fig. 1E). Moreover, we analyzed the relationship between IRE1α activity and patients’ survival in TCGA SKCM database. A total of 456 melanoma patients with available survival data were classified into the high IRE1α-signature and low IRE1α-signature groups based on the optimal cutoff value of IRE1α-signature score (high group includes 204 patients and low group includes 252 patients). The patients with high IRE1α-signature score led a better prognosis than patients with low IRE1α-signature score in the total cohort, as well as in TILs-high subgroups (IRE1α-signature high group includes 135 patients and low group includes 207 patients), but not in TILs-low subgroups (IRE1α-signature high group includes 30 patients and low group includes 84 patients) (Fig. 1F, G). The classification of patients by TILs scores were also based on the optimal cutoff value. Together, these results suggest the close relationship between IRE1α-dependent UPR branch and the feature of TIL in melanoma.

### Tumorous IRE1α facilitates anti-tumor immunity in a CD8^+^T cells-dependent manner

**Fig. 2:**
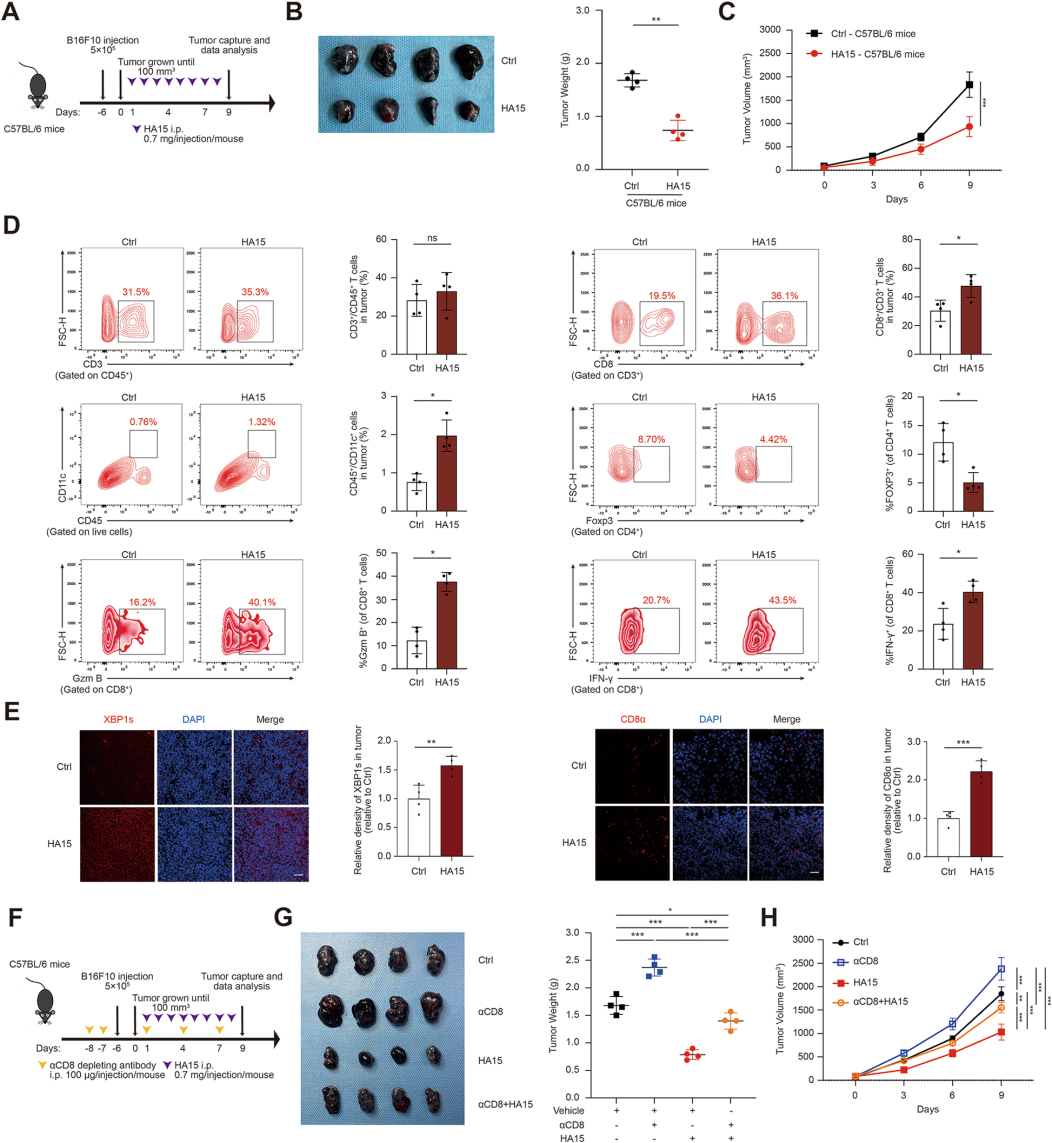
Tumorous IRE1α facilitates anti-tumor immunity in a CD8^+^T cells-dependent manner. **A** Schema of the treatment in C57BL/6 mice bearing B16F10 tumors received HA15 treatment as indicated. Tumor burdens, weights and volumes in each group were calculated and displayed in **B** and **C**. **D** Representative flow cytometry data and summary plots of the frequency of CD3^+^CD45^+^, CD8^+^CD3^+^, CD11c^+^CD45^+^, Foxp3^+^CD4^+^ and CD8^+^T-cells evaluated for expression of Granzyme B and IFN-γ in tumor from xenografts with indicated treatment. **E** Immunofluorescence staining of XBP1s or CD8α in B16F10 xenografts with or without the treatment of HA15. Scale bar, 50 μm. **F**-**H** Scheme representing the experimental procedure (**F**), tumor burdens, tumor weight (**G**), and tumor volume (**H**) of C57BL/6 mice injected subcutaneously with B16F10 tumors with treatment of HA15 and αCD8 depleting antibodies either alone or in combination. Symbols of one dot indicates one mouse, and the error bars are mean with ± SD (*n* = 4). Two-tailed Student’s t-test (*p < 0.05; **p < 0.01; ***p < 0.001; ns, not significant)

Since that IRE1α-dependent UPR branch was positively associated with the feature of TIL in melanoma, we proposed that tumorous IRE1α might display significant influence on anti-tumor immunity and thereby affect melanoma progression. HA15 has been well proved as a potent anti-tumor agent in melanoma through the induction of ER stress via direct interaction with BiP [19]. Our in vitro CCK8 and immunoblotting analysis have revealed that HA15 treatment for 24 h could suppress the cell viability of B16F10 melanoma cells in a dose-dependent manner (Additional file 1: Fig. S2A), and 10 µM HA15 treatment triggered prominent activation of IRE1α branch as revealed by the up-regulation of phosphor-IRE1α and spliced XBP1 (XBP1s) (Fig. S2B), indicating HA15 works properly to induce ER stress and thereby the activation of IRE1α signaling. The increased expression of XBP1s, as well as its transcriptional targets *Erdj4* and *Sec24D*, triggered by HA15 was significantly reversed by IRE1α specific inhibitor STF-083010 (10 µM) or MKC8866 (0.5 µM) of the concentration that induced little reduction of cell viability of B16F10 cell lines (Additional file 1: Fig. S2A-C). Thereafter, B16F10 melanoma cells were inoculated subcutaneously into the flanks of immunocompetent C57BL/6 mice that subsequently received intraperitoneal injection of pharmacological ER stress inducer HA15 (Fig. 2A). In line with previous report [19], HA15 treatment was capable of inducing significant delay of melanoma growth in C57BL/6 mice (Fig. 2B-C). Immune cell composition analysis showed that while HA15 treatment did not alter the total amount of CD3^+^CD45^+^T cells in TME, it significantly increased the infiltration of CD8^+^CD3^+^T cells (Fig. 2D). In addition, the number of CD45^+^CD11c^+^ dendritic cells was prominently increased in TME after HA15 treatment, whereas the number of Foxp3^+^CD4^+^Treg cells was significantly reduced (Fig. 2D). Of note, flow cytometry analysis also revealed that the percentages of Granzyme B^+^ and IFN-γ^+^CD8^+^T cells were increased after the treatment with HA15 (Fig. 2D), indicating the potentiation of the anti-tumor capacity of CD8^+^T cells in TME. In consistent with this, immunofluorescence staining analysis displayed the up-regulation of XBP1s and more infiltration of CD8^+^ T cells in implanted tumor in HA15-treated C57BL/6 mice (Fig. 2E). Moreover, we have also examined the total number of CD8^+^T cells and Treg cells in circulation of mice after HA15 treatment. While the number of tumor-infiltrating CD8^+^T was increased and the number of tumor-infiltrating Tregs was decreased, the numbers of either CD8^+^T cells or Tregs in circulation were not significantly altered (Additional file 1: Fig. S2D-E). Therefore, HA15 might exert its tumor-suppressive role via the increase of CD8^+^T cells in tumor, whereas has no prominent impact on peripheral lymphocytes. To further figure out whether HA15 regulated melanoma growth by modulating the anti-tumor capacity of CD8^+^T cell, specific antibody targeting CD8 were injected intraperitoneally to block CD8^+^T cells systemically in immunocompetent C57BL/6 mice burdened with B16F10 melanoma tumor (Fig. 2F; Additional file 1: Fig. S2F). The elimination of CD8^+^T cells substantially attenuated the tumor-suppressive role of HA15, as revealed by the reverse of tumor volumes and tumor weights (Fig. 2F-H). Taken together, these data have demonstrated that HA15 could promote anti-cancer immunosurveillance to suppress melanoma growth in a CD8^+^T cells-dependent manner.

The dysregulation of systemic inflammation due to the toxicity of some agents might indirectly affect the tumor microenvironment. To assess the systemic toxicity of HA15 treatment in vivo, we measured the serum cytokines, blood cell counts and body weights. As was revealed, the levels of serum cytokines including IL-2, TNFα, and IL-6 after HA15 (0.7 mg/ mice/ day) i.p. administration for 9 days in C57BL/6 mice was not significantly altered compared to the control, indicating the hypo-immunogenicity and minimal effect on immune system of HA15 when delivered systemically (Additional file 1: Fig. S3A). Moreover, the potential safety of HA15 for therapeutic applications was confirmed by the steady-state in blood cell counts of WBC (white blood cells), RBC (red blood cells), HB (hemoglobin), HCT (Hematocrit), MCV (mean corpuscular volume), MCH (mean corpuscular hemoglobin), MCHC (Mean corpuscular hemoglobin concentration), PLT (platelet), NEUT (neutrophil) and LYMPH (lymphocyte) after 9 days of treatment (Additional file 1: Fig. S3B). The body weights of C57BL/6 mice after HA15 treatment also displayed no significant pathological dysregulation (Additional file 1: Fig. S3C). In total, the effect of systemin HA15 application on immune infiltration in TMA is not associated with the alteration of systemic inflammation due to drug toxicity.

Previous studies have revealed that the induction of tumor cell immunologic death or necroptotic death can be helpful to form an inflamed TMA and enhance the anti-tumor immunity [24–27]. To figure out whether these two types of cell death are associated with enhanced anti-tumor immunity induced by HA15 administration, immunofluorescence staining analysis was employed to evaluate the expressions of immunologic death markers Annexin A1, Calreticulin and HMGB1, and necroptotic death marker p-MLKL. As was revealed, there was a slight increase but not statistically significant alteration of the staining intensity of Annexin A1, Calreticulin, HMGB1 and p-MLKL in HA15-treated group compared to the control group (Additional file 1: Fig. S4A-D). For further verification, we conducted experiments to investigate the expression of Annexin A1, Calreticulin, and HMGB1 in B16F10 cells subjected to varying durations of HA15 treatment. The results revealed a significant increase in the staining intensity of calreticulin in HA15-treated B16F10 cells at the 2-hour time point, an up-regulation of HMGB1 mRNA expression in HA15-treated B16F10 cells at 6 h, 12 h, and 24 h, as well as an increase in HMGB1 secretion in HA15-treated B16F10 cells at 12 and 24 h (Additional file 1: Fig. S4E-G). Of note, the increase of calreticulin was transient, and the increase of HMGB1 expression and secretion was significant whereas gradually decreased as the time progressed. Therefore, the anti-tumor effect and enhanced anti-tumor immunity after HA15 treatment in immunocompetent C57BL/6 mice is probably not associated with the occurrence of either immunogenic death or necroptotic death.

**Fig. 3:**
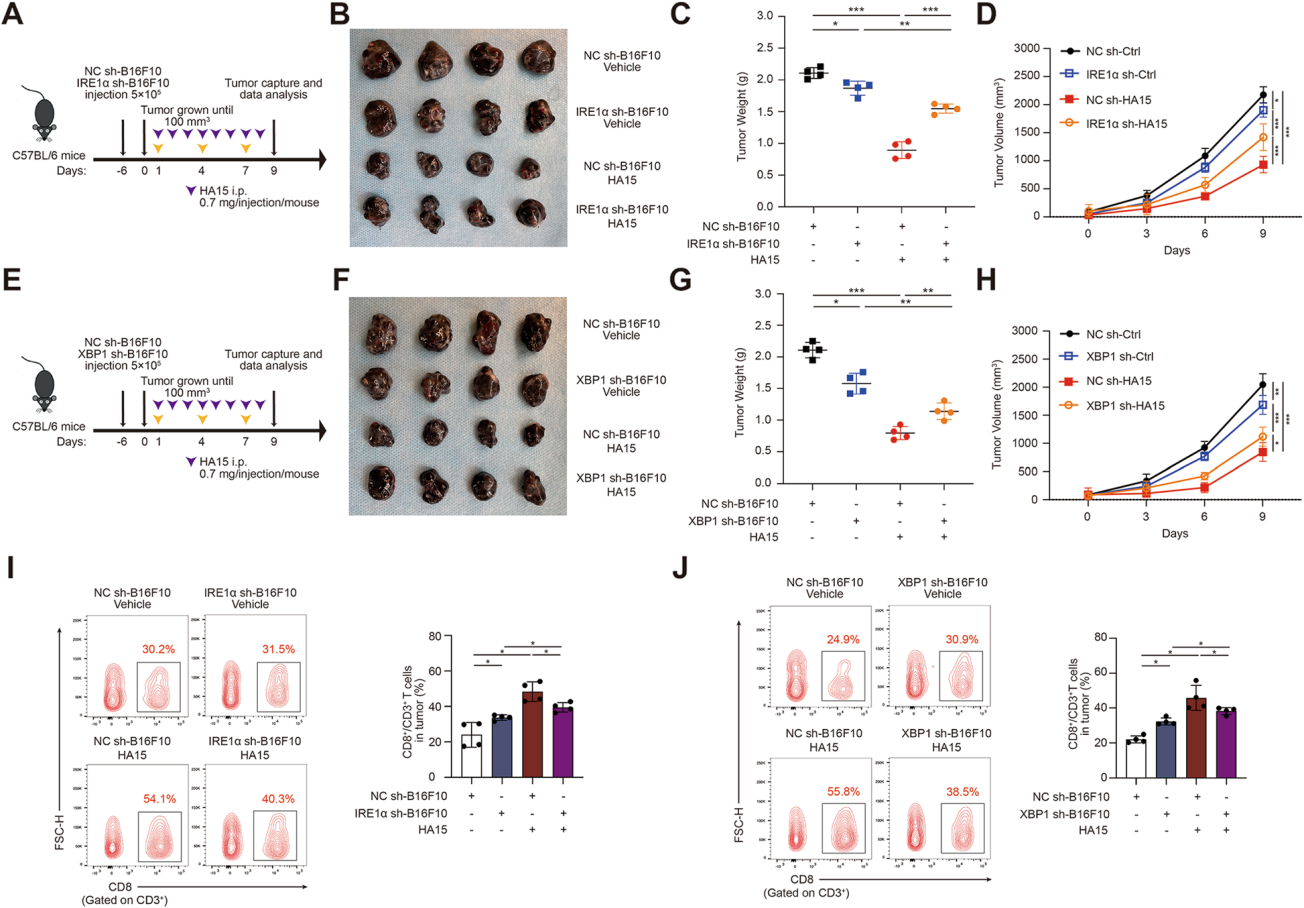
Tumorous IRE1α-XBP1 axis activates CD8^+^T cell-dependent anti-tumor immunity and mediates the tumor-suppressive role of HA15. **A**,** E** Schematic of the treatment in C57BL/6 mice bearing with sh-NC-B16F10, sh-IRE1α-B16F10 or sh-XBP1-B16F10 tumors with or without HA15 treatment as indicated. **B**, **F** Images of isolated tumors from mice that received indicated treatment. Tumor weights and volumes in each group were calculated and displayed in **C**, **G** and **D**, **H**. **I-J** Representative flow cytometry data and summary plots of the frequency of CD8^+^ in tumor from xenografts with indicated treatment. Symbols of one dot indicates one mouse, and the error bars are mean with ± S.D (*n* = 4). Two-tailed Student’s t-test (*p < 0.05; **p < 0.01; ***p < 0.001)

Forwardly, we went on to testify whether the activation of IRE1α-XBP1 axis in tumor mediates the effect of systemic administration of HA15 on tumor growth and anti-tumor immunity in mice. To this end, the knockdown of either IRE1α or XBP1 in B16F10 were obtained individually (Additional file 1: Fig. S5A) and then these cells were inoculated subcutaneously into the flanks of immunocompetent C57BL/6 mice that subsequently received intraperitoneal injection of HA15 (Fig. 3A, E). As was shown, either the deficiency of IRE1α or XBP1 in tumor cell prominently abrogated the role of HA15 in delaying the growth of B16F10 tumor (Fig. 3B-D and F-H). In line with this, either IRE1α or XBP1 deficiency restrained the facilitative effect of HA15 on the recruitment of CD8^+^T cells into TME (Fig. 3I-J). What’s more, we have conducted an evaluation of the number of regulatory T cells (Tregs) or the percentage of Granzyme B^+^ and IFN-γ^+^CD8^+^T cells in HA15-treated tumor harboring IRE1α or XBP1 deficiency using immunofluorescence staining analysis. While HA15 treatment led to substantially reduction of Foxp3^+^CD4^+^ Tregs in TME, either the deficiency of IRE1α or XBP1 in tumor cells could partially reverse this alteration (Additional file 1: Fig. S5B). In addition, HA15 treatment also resulted in robust increase of both Granzyme B^+^ and IFN-γ^+^ CD8^+^T cells in TME compared with control, but the knockdown of either IRE1α or XBP1 in tumor cells significantly mitigated this facilitative effect (Additional file 1: Fig. S5C-D). Taken together, tumorous IRE1α-XBP1 axis activates CD8^+^T cell-dependent anti-tumor immunity and mediates the tumor-suppressive role of HA15.

### Tumorous IRE1α promotes the secretion of Th1-related chemokine and cytokines by activating NF-κB

**Fig. 4:**
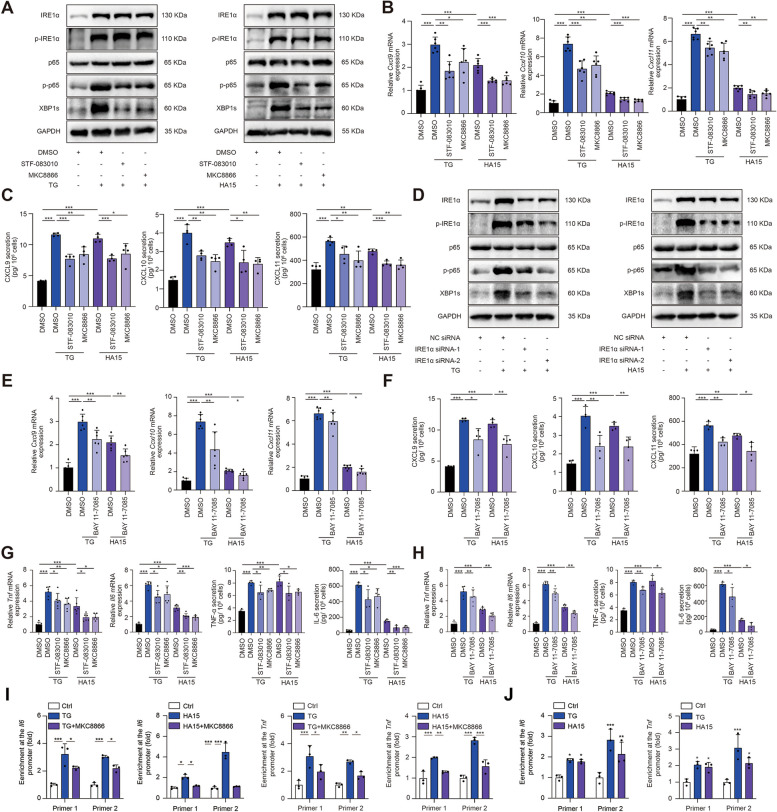
Tumorous IRE1α promotes the secretion of Th1-related chemokine and cytokines by activating NF-κB. **A**, **D** Immunoblotting analysis of IRE1α, p-IRE1α, p65, p-p65, XBP1s and GAPDH in A2058 cells treated with TG (0.5 µM) or HA15 (10 µM) for 24 h after pretreated with or without STF-083010 (10 µM), MKC8866 (0.5 µM) or IRE1α siRNA for 24 h. **B**-**C**, **E**-**H** Relative mRNA level (*n* = 6) and ELISA (*n* = 4) analysis of CXCL9, CXCL10, CXCL11, TNF-α and IL-6 in A2058 cells treated with TG (0.5 µM) or HA15 (10 µM) for 24 h after pretreated with or without STF-083010 (10 µM), MKC8866 (0.5 µM) or BAY 11-7085 (1 µM) for 24 h. **I** A2058 cells treated with TG (0.5 µM) or HA15 (10 µM) for 24 h after pretreated with or without MKC8866 (0.5 µM) for 24 h were subjected to ChIP with normal mouse IgG, NF-κB or Pol-II antibody as indicated (*n* = 3). **J** A2058 cells treated with TG (0.5 µM) or HA15 (10 µM) for 24 h were subjected to ChIP with normal mouse IgG, XBP1s or Pol-II antibody as indicated (*n* = 3). ChIP samples were analyzed by qPCR using primers indicated in Additional file 2: Table S3. Data are representative of at least three independent experiments and shown as mean ± SD. Two-tailed Student’s t-test or two-way ANOVA (*p < 0.05; **p < 0.01; ***p < 0.001)

The infiltration and anti-tumor function of CD8^+^T cells in TME can be regulated by chemokine-dependent recruitment signaling and pro-inflammatory cytokines-mediated activation. To be specific, CXCL9, CXCL10 and CXCL11 that are known as IFN-γ-induced chemokines that can bind to the CXCR3 receptor on CD8^+^T cells to drive their recruitment to tumor bed [28]. To figure out whether these chemokines mediate tumor IRE1α activation-induced increased infiltration of CD8^+^T cells in melanoma under ER stress, we testified the alterations of these chemokines in response to the treatment with either TG or HA15 in both A2058 and A375 melanoma cell lines. Similar to that in B16F10, either the treatment with TG or HA15 for 24 h could suppress cell viability in a dose-dependent manner in both A2058 and A375 melanoma cells (Additional file 1: Fig. S6A). The sublethal concentration of TG or HA15 that triggered ~ 20% reduction of cell viability (TG, 0.5 µM for A2058 and 0.2 µM for A375; HA15, 10 µM for both A2058 and A375) could induce prominent activation of IRE1α-XBP1 axis, as was shown by increased expressions of phosphor-IRE1α and XBP1s, as well as downstream transcriptional targets of XBP1, *Erdj4*, *Sec61A* and *p58IPK* (Fig. 4A; Additional file 1: Fig. S6B-C). As was revealed, either treatment with TG or HA15 could robustly promote the transcriptional level of *Cxcl9*, *Cxcl10* and *Cxcl11* in both A2058 and A375 cell lines (Fig. 4B; Additional file 1: Fig. S6D). In line with this, enzyme-linked immune sorbent (ELISA) assay also revealed that the secretion of CXCL9, CXCL10 and CXCL11 in culture supernatant was also prominently enhanced in response to ER stress inducers (Fig. 4C; Additional file 1: Fig. S6E). Furthermore, we also confirmed that the expression and secretion of the above chemokines in B16F10 cells were increased under ER stress induction (Additional file 1: Fig. S7A-D). It has been proved that the IRE1α branch of UPR was in positive correlation with TIL signature in melanoma, which is also supported by a previous report [29]. Therefore, we hypothesized that IRE1α-XBP1s axis might be involved in ER stress-induced chemokine production. To this end, IRE1α specific inhibitors STF-083010 and MKC8866 were employed in TG/HA15-stimulated melanoma cells to block IRE1α-XBP1 axis. Of note, either STF-083010 or MKC8866 treatment for 24 h would also induce the reduction of cell viability as the concentration increases (Additional file 1: Fig. S6F), and the concentration that triggers little impairment of cell viability (STF-083010, 10 µM for both A2058 and A375; MKC8866, 0.5 µM for both A2058 and A375) could efficiently suppress TG/HA15-induced up-regulation of XBP1s, as well as XBP1 targets *ERDJ4*, *Sec61A* and *p58IPK* (Fig. 4A; Additional file 1: Fig. S6B-C). Either STF-083010 or MKC8866 treatment could significantly reverse the increase of both the transcription and secretion of CXCL9, CXCL10 and CXCL11 in A2058, A375 and B16F10 cell lines (Fig. 4B-C; Additional file 1: Fig. S6D-E; Additional file 1: Fig. S7A-B).

Since that there is no potential binding region of XBP1 at the promoter of *CXCL9*, *CXCL10* or *CXCL11* (data not shown), alternative transcriptional factor downstream XBP1 should mediate the transcriptional activation of these chemokines in response to ER stress. It has been reported that NF-κB is a critical transcriptional factor of CD8^+^T cell chemokines [30–32], and could be regulated by IRE1α-XBP1 axis in response to ER stress inducers and other stimuli [33–36]. Therefore, we proposed that NF-κB might connect IRE1α-XBP1 axis to downstream chemokines up-regulation. Our immunoblotting analysis showed that while the phosphorylation of NF-κB p65 subunit was increased after the treatment with either TG or HA15, either the knockdown of IRE1α or the treatment with STF-083010/MKC8866 could prominently reverse this alteration (Fig. 4A, D; Additional file 1: Fig. S6B, G). BAY-11-7085 is a specific inhibitor of NF-κB. We employed 1 µM BAY-11-7085 treatment for 24 h in both A2058, A375 and B16F10 cell lines that triggered little reduction of cell viability (Additional file 1: Fig. S2A; Fig. S6H), whereas prominently suppressed the phosphorylation of p65 under ER stress (Additional file 1: Fig. S6I). BAY-11-7085 treatment was capable of suppressing TG/HA15-induced increased transcription and secretion of chemokines CXCL9, CXCL10 and CXCL11 in A2058, A375 and B16F10 cell lines (Fig. 4E-F; Additional file 1: Fig. S6J-K; Additional file 1: Fig. S7C-D). Therefore, IRE1α-NF-κB pathway mediates the facilitative role of ER stress in chemokine expression and production in melanoma.

Apart from chemokines, the anti-tumor capacity of CD8^+^T cells was largely regulated by pro-inflammatory cytokines such as IL-6 and TNF-α [37]. We found that in response to TG or HA15 treatment, both the transcription and secretion of IL-6 and TNF-α were prominently increased in both A2058 and A375 melanoma cell lines (Fig. 4G; Additional file 1: Fig. S6L). Pharmacological inhibition of either IRE1α or NF-κB by STF-083010/ MKC8866 and BAY-11-7085 respectively could significantly suppress TG or HA15-induced increased transcription and secretion of both IL-6 and TNF-α in both A2058 and A375 melanoma cell lines (Fig. 4G-H; Additional file 1: Fig. S6L-M).

The bioinformatics analysis unveiled that there are potential binding sites of NF-κB p65 subunit in the promoters of *IL-6* and *TNF-α* (Additional file 1: Fig. S8A). Chromatin-immunoprecipitation assay confirmed the binding of NF-κB p65 subunit to the promoter regions, which could be potentiated by TG and HA15 and re-reversed by MKC8866 (Fig. 4I; Additional file 1: Fig. S8B). These results suggested that the enhanced expression of IL-6 and TNF-α that related to activated CD8^+^T cells under ER stress was dependent on tumorous IRE1α-NF-κB pathway. Of note, some previous reports have demonstrated that XBP1s can directly bind to the promoters of both *IL-6* and *TNF-α* [38, 39], which is also predicted by the bioinformatic analysis (Additional file 1: Fig. S8C). Our ChIP assay proved the direct binding of XBP1s to the promoters of both *IL-6* and *TNF-α*, which could be potentiated by either TG or HA15 treatment (Fig. 4J; Additional file 1: Fig. S8D). Therefore, apart from the regulation by NF-κB downstream of IRE1α, the expression and secretion of IL-6 and TNF-α might also be directly transcriptionally regulated by XBP1 downstream of IRE1α in melanoma undergoing ER stress.

According to previous report, the activation of RIDD (Regulated IRE1α-dependent decay) downstream of IRE1α activation could promote the activation of NF-κB via RIG-1 [40]. Therefore, we speculate that apart from the regulation by XBP1, the activation of NF-κB downstream IRE1α might also be mediated by RIG-1. To this end, we first testified the activation of RIDD in response to TG treatment in melanoma cells. As was revealed, the expressions of typical IRE1α RIDD targets, *Blos1*, *Scara3*, *Pmp2* and *Col6a1*, were significantly down-regulated after TG treatment, indicating the activation of RIDD (Additional file 1: Fig. S9A). Meanwhile, the expression of *RIG-1* was also significantly increased after the treatment with TG, and this alteration was mitigated after the co-treatment with STF-083010 or MKC8866 (Additional file 1: Fig. S9B-C), confirming the activation of RIG-1 paralleled with RIDD activation was dependent on IRE1α endoribonuclease activity. Then, we obtained the knockdown of RIG-1 in melanoma cells (Additional file 1: Fig. S9D), which significantly reduced the phosphorylation of p65 induced by TG treatment (Additional file 1: Fig. S9E). In consistent with this, the up-regulation of the mRNA levels of pro-inflammatory chemokines and cytokines downstream of NF-κB, including *Cxcl9*, *Cxcl10*, *Cxcl11*, *Tnf* and *Il6*, were also significantly mitigated by the knockdown of RIG-1 after TG treatment (Additional file 1: Fig. S9F). Therefore, the activation of RIDD downstream of IRE1α is also responsible for NF-κB activation in melanoma in response to ER stress.

### Tumorous IRE1α-dependent NF-κB activation contributes to the activation of CD8^+^T cells

**Fig. 5:**
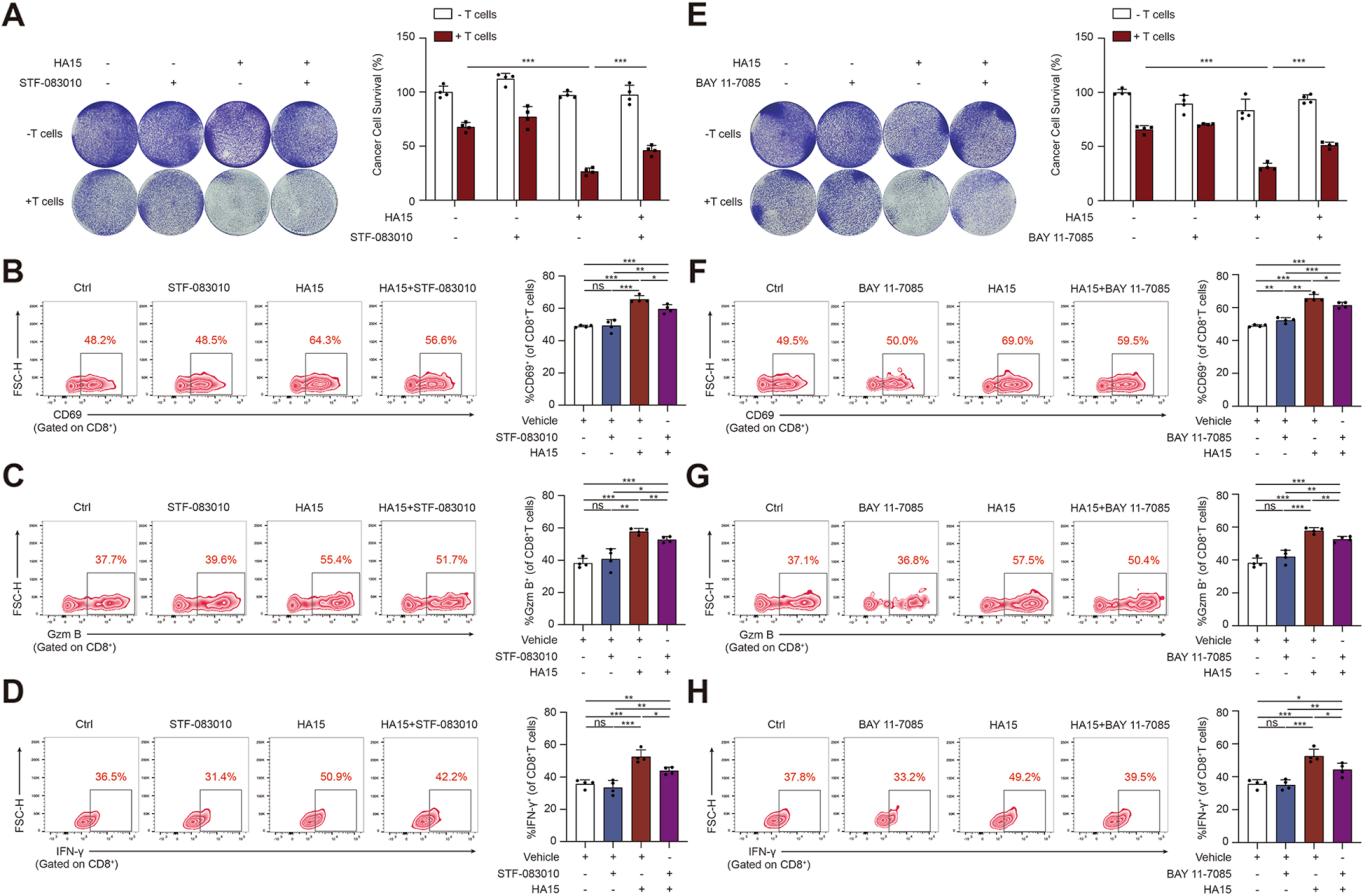
Tumorous IRE1α-induced potentiated activation of CD8^+^T cells was mediated by NF-κB. **A**, **E** A2058 melanoma cells treated with HA15 (10 µM) for 24 h after pre-treated with or without STF-083010 (10 µM) or BAY 11-7085 (1 µM) for 24 h cocultured with or without activated T cell (1:3) for 24 h were subjected to crystal violet staining. Cytotoxicity was quantified by a spectrometer at OD (570 nm) and normalized ratio of cancer cell survival was shown for each well (*n* = 4). **B-D**, **F-H** Representative flow cytometry data and summary plots of the frequency of CD8^+^ T cells evaluated for expression of CD69, Granzyme B and IFN-γ in co-culture system with indicated treatment (*n* = 4). Data are representative of four independent experiments and shown as mean ± SD. Two-tailed Student’s t-test (*p < 0.05; **p < 0.01; ***p < 0.001; ns, not significant)

To further prove that tumorous IRE1α-induced potentiated activation of CD8^+^T cells was dependent on NF-κB signaling, co-culture system harboring both melanoma cells and PBMCs were employed, in which A2058 or A375 melanoma cells were pre-stimulated with HA15 (10 µM for both A2058 and A375) for 24 h to induce intracellular ER stress and UPR activation, and meanwhile triggered little reduction of cell viability (Additional file 1: Fig. S6A). Compared to control, HA15-stimulated A2058 or A375 melanoma cells were more sensitive to the killing by CD8^+^T cells (Fig. 5A; Additional file 1: Fig. S10A). In addition, the percentage of CD69, Granzyme B and IFN-γ in CD8^+^T cells in co-culture system were also potentiated robustly (Fig. 5B-D; Additional file 1: Fig. S10B-D). More importantly, pre-treatment of melanoma cells with either IRE1α inhibitor STF-083010, MKC8866 or NF-κB inhibitor BAY-11-7085 (STF-083010, 10 µM for both A2058 and A375; MKC8866, 0.5 µM for both A2058 and A375; BAY-11-7085, 1 µM for both A2058 and A375) could reverse the above-mentioned alteration of CD8^+^T cells-mediated killing capacity of melanoma cells (Fig. 5A-H; Additional file 1: Fig. S10A-H; Additional file 1: S11A-H). In aggregate, tumorous IRE1α promoted the recruitment and functional activation of surrounding CD8^+^T cells in NF-κB pathway-dependent manner in melanoma undergoing ER stress.

### Tumorous IRE1α up-regulates PD-L1 expression via NF-κB pathway upon ER stress

PD-L1 is a critical immune checkpoint that is associated with tumor immune evasion and has been documented as a valuable biomarker for predicting the response to anti-PD-1 immunotherapy [41]. The correlation analysis in TCGA SKCM database revealed that the UPR component XBP1 that reflects the activity of IRE1α was highly associated with the expression of PD-L1 (encoded by *CD274*) (Additional file 1: Fig. S12A). Therefore, we hypothesize that tumorous IRE1α might also mediate PD-L1 expression to keep the immune reaction in balance, in addition to its role in promoting the secretion of chemokines and cytokines to amplify the anti-tumor capacity of CD8^+^T cells. qRT-PCR and immunoblotting analysis showed that either TG or HA15 treatment induced prominent up-regulation of PD-L1 at both the mRNA and protein levels (Additional file 1: Fig. S12B-C). In addition, flow cytometry analysis also revealed that membrane PD-L1 level was also prominently induced in response to the treatment with either TG or HA15 (Additional file 1: Fig. S12D). Since that the correlation between XBP1 and PD-L1, as well as IRE1α and PD-L1, was more prominent compared to the correlation between ATF6/ PERK and PD-L1 in TCGA SKCM database (Additional file 1: Fig. S12A), we speculated that IRE1α branch of UPR might mediate ER stress-induced increase of PD-L1. To this end, ER stress-stimulated melanoma cells were pre-treated with IRE1α specific inhibitor STF-083010 or MKC8866, and the increased PD-L1 expression induced by TG or HA15 was significantly suppressed by the inhibition of IRE1α in either transcriptional level or protein level (Additional file 1: Fig. S12E-F). Moreover, flow cytometry analysis showed that the up-regulation of membrane level of PD-L1 in response to ER stress was also diminished after the inhibition of IRE1α (Additional file 1: Fig. S12G), further supporting that tumorous IRE1α promotes the expression of PD-L1 upon ER stress. Of note, it has been reported that NF-κB pathway could be activated downstream of IRE1α and this regulatory effect was confirmed by our previous results (Fig. 4A, D; Additional file 1: Fig. S6B, G) [42, 43]. In addition, the transcription of PD-L1 could be positively regulated by NF-κB [44, 45]. Therefore, it was proposed that NF-κB might mediate the role of IRE1α in the regulation of PD-L1 expression under ER stress. TG or HA15-stimulated melanoma cells were pre-treated with NF-κB inhibitor BAY-11-7085, which revealed that the increased expressions of PD-L1 at either mRNA, protein or membrane level were all prominently suppressed by the inhibition of NF-κB in both A2058 and A375 cell lines (Additional file 1: Fig. S12H-J). In aggregate, these results demonstrated that tumorous IRE1α up-regulates PD-L1 expression via NF-κB pathway under ER stress. This alteration might be a mechanism to keep the immune reaction in balance.

### ER stress inducer enhances the anti-tumor activity of anti-PD-1 antibody in vivo

**Fig. 6:**
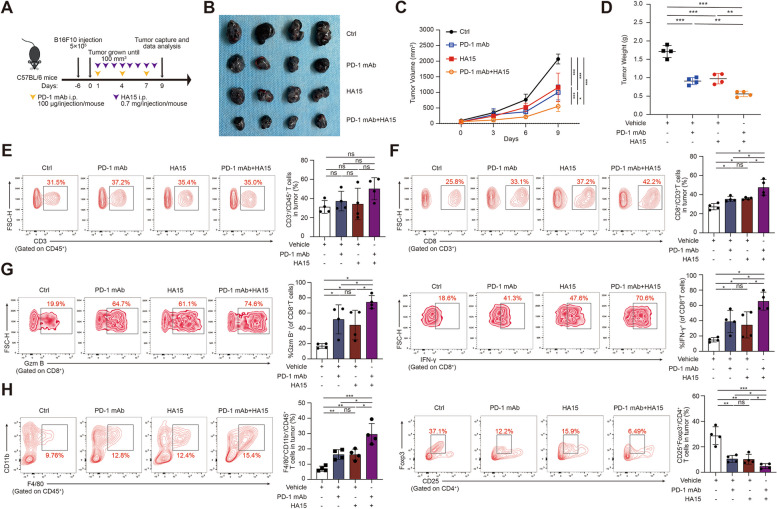
ER stress inducer enhances the anti-tumor activity of anti-PD-1 antibody in vivo. **A** Schema of the treatment in C57BL/6 mice bearing B16F10 tumors received HA15 with or without anti-PD-1 antibody combination treatment as indicated. **B** Images of isolated tumors from mice that received indicated treatment. Tumor volumes and weights in each group were calculated and displayed in **C** and **D**. **E**-**H** Representative flow cytometry data and summary plots of the frequency of CD3^+^CD45^+^, CD8^+^CD3^+^, F4/80^+^CD11b^+^CD45^+^, Foxp3^+^CD25^+^CD4^+^ and CD8^+^T-cells evaluated for expression of Granzyme B and IFN-γ in tumor from xenografts with indicated treatment. Symbols of one dot indicates one mouse, and the error bars are mean with ± S.D (*n* = 4). Two-tailed Student’s t-test (*p < 0.05; **p < 0.01; ***p < 0.001; ns, not significant)

Our previous results have unveiled that the treatment with ER stress inducer HA15 can facilitate the infiltration and activation of cytotoxic CD8^+^T cells to enhance the anti-tumor capacity, which is predicted to be associated with potentiated treatment efficacy of anti-PD-1 immunotherapy [46, 47]. To prove this, C57BL/6 mice were subcutaneously implanted with B16F10 melanomas and then received the combination of both HA15 and anti-PD-1 antibody treatment (Fig. 6A). While the monotherapy with either HA15 or anti-PD-1 antibody could slow down tumor growth, the combination group exhibited a more pronounced suppression of tumor volumes and tumor weights (Fig. 6B-D), supporting the conclusion that ER stress inducer enhances the anti-tumor activity of anti-PD-1 antibody in vivo. Through flow cytometry analysis of the suspensions of isolated tumors, it was uncovered that while the infiltration of CD3^+^CD45^+^T cells was not significantly altered in combined treatment group compared with mono-treatment group and control, the number of CD3^+^CD8^+^T cells was increased in TME (Fig. 6E-F). Of note, the percentage of either Granzyme B-positive or IFN-γ-positive CD8^+^T cells was also significantly potentiated (Fig. 6G), suggesting the enhanced anti-tumor capacity of CD8^+^T cells after the combined treatment. What’s more, it was observed that the infiltration of F4/80^+^CD11b^+^ macrophages was increased, whereas the percentage of Foxp3^+^CD25^+^ in CD4^+^T cells was down-regulated in combined treatment group compared to mono-treatment group and control (Fig. 6H). Therefore, the numbers of macrophages and Treg cells were also accordingly altered to mediate the enhanced anti-tumor capacity after the combined treatment.

## Discussion

**Fig. 7:**
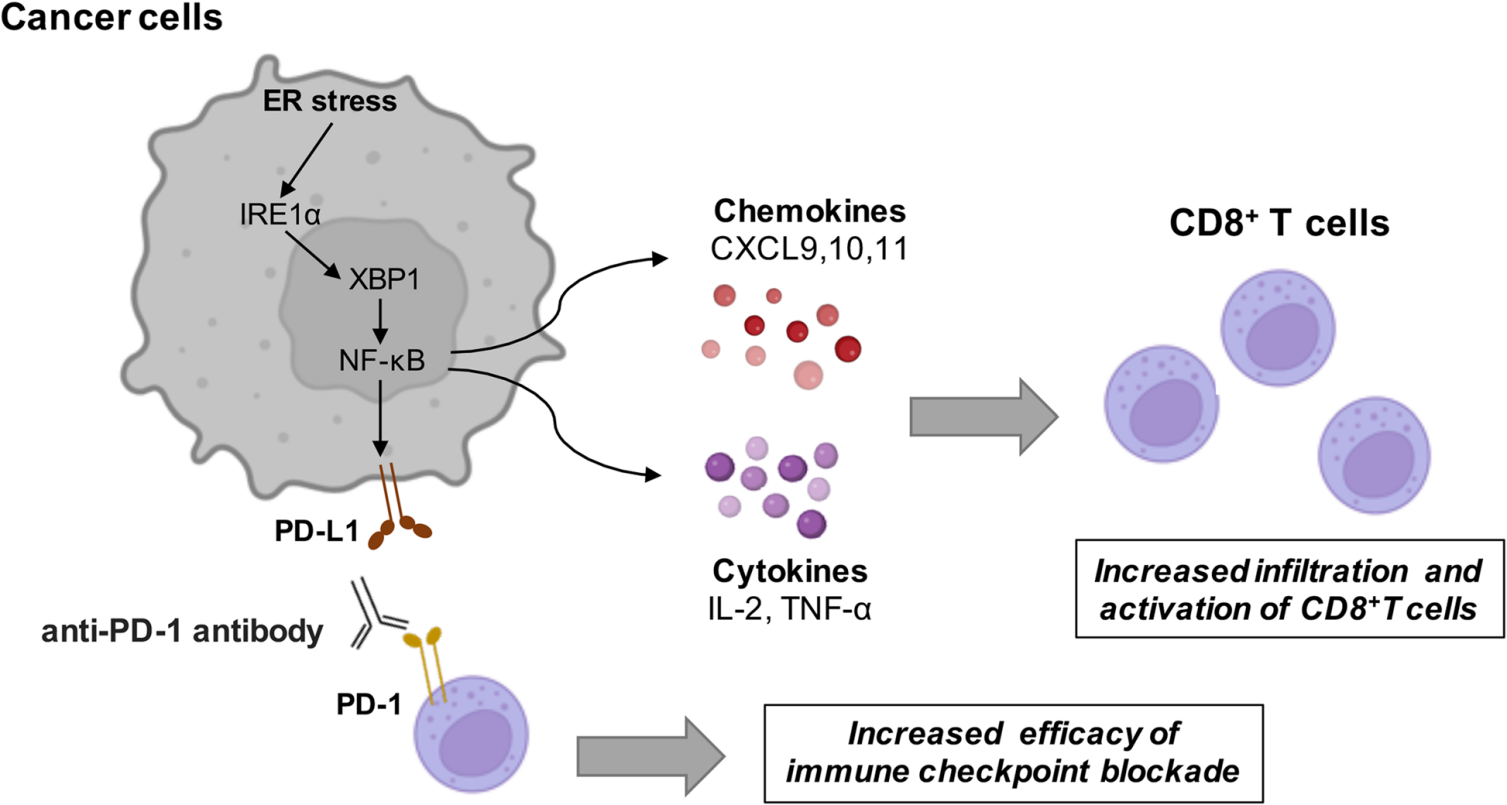
A schematic model summarizing tumorous IRE1α facilitates CD8^+^T cells-dependent anti-tumor immunity and improves immunotherapy efficacy in melanoma

In the present study, we firstly discovered that the IRE1α branch of UPR was in positive correlation with TIL score in melanoma. Then, we proved that pharmacological induction of ER stress by HA15 exerted better anti-tumor effect in immunocompetent mice and was highly dependent on tumor-infiltrating CD8^+^T cells. In parallel, the profile of immune cells in TME was significantly re-shaped, revealing the feature of potentiated anti-tumor immunity. Subsequent mechanistic studies showed that tumorous IRE1α facilitated the expression and secretion of Th1-related chemokines and cytokines by activating NF-κB in melanoma cells, which was highly associated with the recruitment and activation of CD8^+^T cells. Ultimately, the effect of the combination of ER stress inducer and anti-PD-1 antibody was confirmed in pre-clinical mice model. Taken together, these results demonstrate that tumorous IRE1α can facilitate CD8^+^T cells-dependent anti-tumor immunity by promoting the expression and secretion of Th1-related chemokines and cytokines. The employment of ER stress inducer can robustly improve the efficacy of anti-PD-1 immunotherapy in melanoma by eliciting pro-inflammatory immune microenvironment (Fig. 7).

During the process of tumor growth and metastasis, melanoma cells frequently suffer from ER stress resulted from both endogenous and exogenous sources. On one hand, the synthesis of proteins in mutated forms that are required for tumor malignancy is increased in rapidly-proliferating tumor cells, inducing the accumulation of misfolded proteins [48]. On the other, tumor microenvironment is usually characterized by stressful conditions like hypoxia, acidosis and nutrients deprivation that can elicit ER stress [49]. In response, the sustained activation of IRE1α and ATF6 branches driven by MEK/ERK pathway endows tumor cells with enhanced survival capacity [50, 51], with the involvement of the activation of downstream autophagy, mitochondrial fission, mitophagy and JAK/ STAT3 pathway [11, 17, 18, 52]. Apart from the protective role in tumor cell survival, the activation of UPR also contributes to the formation of metastases and the resistance to current available therapy in melanoma. Specifically, the activities of the three main branches of the UPR were all increased in metastatic compared to non-metastatic melanoma cells. Pharmacological antagonization of the UPR via 4-PBA resulted in the reduction of FGF expression and the impairment of melanoma cell migration [53]. In addition, shortly after BRAF inhibitor treatment, a PERK-dependent UPR would be initiate to activate cyto-protective autophagy, which elicits lower response rates to treatment and a shorter duration of progression-free survival of patients [54]. Therefore, the tumorigenic effects of UPR have been well elucidated in previous studies, with particular emphasis on the regulation of tumor cells themselves. Recently, a body of literature has indicated that cancer cell-intrinsic UPR can influence the function of immune cells that coexist in the TME. The IRE1α-XBP1 arm represses the expression of the NKG2D ligand MHC class I polypeptide-related sequence A (MICA) in human melanoma cell lines undergoing ER stress [55]. Besides, the activation of the PERK-eIF2α arm of the UPR in melanoma cells undergoing ER stress could induce the expression of B7H6, which is a ligand for the NK cell receptor NKp30 [56]. Accordingly, ER-stressed melanoma cells overexpressing B7H6 were sensitized to killing by CAR-T cells specifically redirected against this ligand. In contrast to these reports, some evidence supports that melanoma cells with UPR activation could significantly up-regulate immunosuppressive Arginase 1 and PGE2 in DCs while simultaneously inhibit their capacity to cross-present antigens to CD8^+^T cells [57], which termed “transmissible ER stress” [58]. In this regard, the role of tumorous UPR in anti-tumor immunity remains under debate from the perspective of the regulation of the function of DCs. Our present study has demonstrated that tumorous IRE1α significantly potentiated anti-tumor immunity in response to ER stress, which was highly associated with increased expression and secretion of pro-inflammatory cytokines and chemokines triggered by XBP1 and NF-κB downstream of IRE1α. Meanwhile, it was also observed that the infiltration of F4/80^+^CD11b^+^ macrophages in TME was also increased, suggesting that apart from CD8^+^T cells-dependent adaptive immune response, innate immune components like macrophages can also be responsible for the anti-tumor effect of HA15. The destruction and elimination of tumor cells by HA15 might also lead to the generation and exposure of neo-antigen to assist in the development of T cells-dependent adaptive immune response. What’s more, IRE1α branch of UPR was highly associated with TIL score in TCGA SKCM database. In aggregate, our data provided both genomic and functional study evidence supporting the facilitative role of tumorous IRE1α in anti-tumor immunity in melanoma. Pharmacological induction of ER stress in tumor is a valuable strategy to elicit and amplify tumor immunology.

The effects of UPR in multiple cancer-associated immune cells and anti-tumor immunity have been gradually revealed. For example, IRE1α-XBP1 over-activation in dendritic cells could disrupt the lipid metabolic homeostasis and cripple antigen presentation to T cells, thereby impeding the protective immune responses against tumor cells in ovarian cancer [59]. In line with this, antigen-derived hydrophobic peptides could directly engage ER-resident IRE1α, the activation of which depletes MHC-I heavy-chain mRNAs through IRE1α-dependent decay (RIDD), curtailing antigen cross-presentation. The disruption of IRE1α increased MHC-I expression on tumor-infiltrating DCs and enhanced recruitment and activation of CD8^+^ T cells [60]. In addition, neutrophils and MDSCs could utilize IRE1α-XBP1 and CHOP respectively to promote the expression of arginase that can actively suppress the function of T cells within TME [61]. In melanoma, tumor-MDSCs harbor higher activation of PERK, the deletion of which transformed MDSCs into myeloid cells that activated CD8^+^T cell-mediated immunity against tumor cells [62]. More importantly, tumor tissues enriched with cholesterol in tumor-infiltrating CD8^+^T cells induced prominent ER stress and consequently XBP1-dependent transcription of PD-1 and 2B4, leading to the exhaustion of CD8^+^T cells and impaired anti-tumor immunity [63]. In contrast to the above-mentioned reports that all documented the immunosuppressive effects of ER stress and UPR in multiple cancer-associated immune cells, the activation of IRE1α-XBP1 is necessary for the optimal proliferative capacity of NK cells via the activation of c-Myc under homeostatic conditions and in the setting of melanoma models. The establishment of transplanted B16F10 tumors into conditional knockout mice lacking IRE1α or XBP1 in NK cells resulted in decreased intra-tumoural NK cell infiltration, increased lung nodules and reduced host survival [64]. Therefore, the role of UPR in tumor-infiltrating immune cells is in a context-dependent manner, which suggests that the intervention of UPR in TME should take the type of immune cells into consideration. While tumor microenvironment of melanoma is a multi-component and complex network consisting of tumor cells, keratinocytes, adipocytes, and various types of immune cells [3, 4], the quantity of tumor cells dominates within TME and they bear the most part of ER stress. In this regard, the regulatory role of tumorous UPR in anti-tumor immunity might be more prominent than immune cells’ intrinsic UPR. Of note, we did not observe the impairment of the number or the function of infiltrating CD8^+^ T cells in xenograft tumor undergoing ER stress, indicating that the pharmacological induction of ER stress in our pre-clinical mice model is proper that can induce robust UPR in tumor whereas not intervene the cytotoxicity of CD8^+^T cells. Th1-related chemokines and pro-inflammatory cytokines act as the bridge between tumorous UPR and surrounding immune cells. Other secretory intermediates like exosomes might also play crucial role in transducing UPR signaling in tumor to infiltrating immune cells, which needs further investigations.

Previously, some reports have also elucidated the role of ER stress in the activation of NF-κB, as well as the underlying mechanisms. To be specific, Kaneko et al. has proved that ER stress-induced NF-kB activation is dependent on the interaction between IRE1α and TRAF2, and the kinase activity of IRE1α is greatly implicated in [65]. Then, the results obtained by Tam et al. revealed that IRE1α acts to maintain IKK (the inhibitor of NF-κB) basal activity through kinase activity instead of RNase activity. Inputs from IRE1α and IKK, in combination with translation repression by PERK, another UPR initiator, lead to maximal NF-κB activation during the UPR [66]. Therefore, apart from the reports that NF-κB could be regulated by IRE1α-XBP1 axis that is dependent on the endonuclease activity of IRE1α [33–36], IRE1α might also participate in the regulation of NF-κB directly via its kinase activity in melanoma cells undergoing ER stress.

In the present study, the induction of PD-L1 expression by IRE1α under ER stress in tumor is regarded as the bypass adaptive alteration to keep the immune reaction in balance, whereas is not the reason for tumor IRE1α-mediated anti-cancer immunosurveillance. Actually, the increased expression and secretion of pro-inflammatory chemokines and cytokines caused by IRE1α activation are largely responsible for the potentiated capacity of lymphocytes to eradicate tumor cells. While not contributes to the potentiated anti-tumor immunity, the up-regulation of tumor PD-L1 under ER stress could provide the molecular basis for the employment of anti-PD-1 antibody to block the interaction between PD-L1 and PD-1, and high PD-L1 expression is regarded as a promising biomarker for predicting better treatment outcome of immunotherapy [67]. Therefore, the induction of PD-L1 by tumorous IRE1α under ER stress is not the main reason for potentiated anti-tumor capacity of CD8^+^T cells, but helps to provide the molecular basis for the increase of immunotherapy efficacy.

There are some limitations of the present study as follows: First, the up-regulation of PD-L1 expression driven by tumorous IRE1α under ER stress seems to be contrary to the facilitative role of tumorous IRE1α in the activation of CD8^+^T cells. We believe that this phenomenon is actually a bypass adaptive alteration to keep the immune reaction in balance. While not contributes to the activation of anti-tumor immunity, the up-regulation of PD-L1 could provide the molecular basis for the employment of anti-PD-1 antibody immunotherapy along with ER stress inducer. Second, although the results obtained from in vitro co-culture system have proved that tumorous IRE1α robustly potentiated the anti-tumor capacity of CD8^+^T cells, and the killing effect seems to be synergic compared to either the mono-treatment with sublethal HA15 or the only presence of T cells, additional system that could testify antigen-specific killing of CD8^+^T cells should be employed in the future to provide more convincing evidence. Last but not least, apart from CD8^+^T cells in TME, alternative types of immune cells need to be analyzed to reveal the comprehensive regulatory effect of tumorous IRE1α on anti-tumor immunity.

Taken together, our present study demonstrates that tumorous IRE1α facilitates anti-cancer immunosurveillance and improves immunotherapy efficacy in melanoma via the regulation of chemokines, cytokines and PD-L1 expression. Pharmacological induction of ER stress is a promising strategy to amplify anti-tumor immunity and increase the efficacy of anti-PD-1 antibody, which needs forward investigations in the future.

### Supplementary Information

** Additional file 1.** Supplementary figures and figure legends.

** Additional file 2:** **Table 1.** Clinicopathologic characteristics of melanoma patient cohorts, related to Fig. 1. **Table 2.** Primary Antibodies Used. **Table 3.** Primers used for qPCR.

** Additional file 3.** Supplementary Materials and methods.

** Additional file 4.** Original and uncropped films of Western blots.

## Data Availability

The data that support the findings of this study are available upon request from the corresponding author.

## Abbreviations

TME: Tumor microenvironment
ER: Endoplasmic reticulum
ATCC: American Type Culture Collection
DMEM: Dulbecco’s modified Eagle’s medium
STR: Short-tandem repeat
MFI: Mean fluorescence intensity
PBMCs: Human peripheral blood mononuclear cells
SKCM: Skin cutaneous melanoma
ELISA: Enzyme-linked immune sorbent
