# Supplementary material for: Tumorous IRE1α facilitates CD8+T cells-dependent anti-tumor immunity and improves immunotherapy efficacy in melanoma
Source: Cell Commun Signal. 2024 Jan 30;22:83. doi: 10.1186/s12964-024-01470-8 (PMC10826282; doi:10.1186/s12964-024-01470-8)
Supplement: Supplementary file 1 — Additional file 1. Supplementary figures and figure legends. [file 12964_2024_1470_MOESM1_ESM.docx]

**Additional file 1**

**
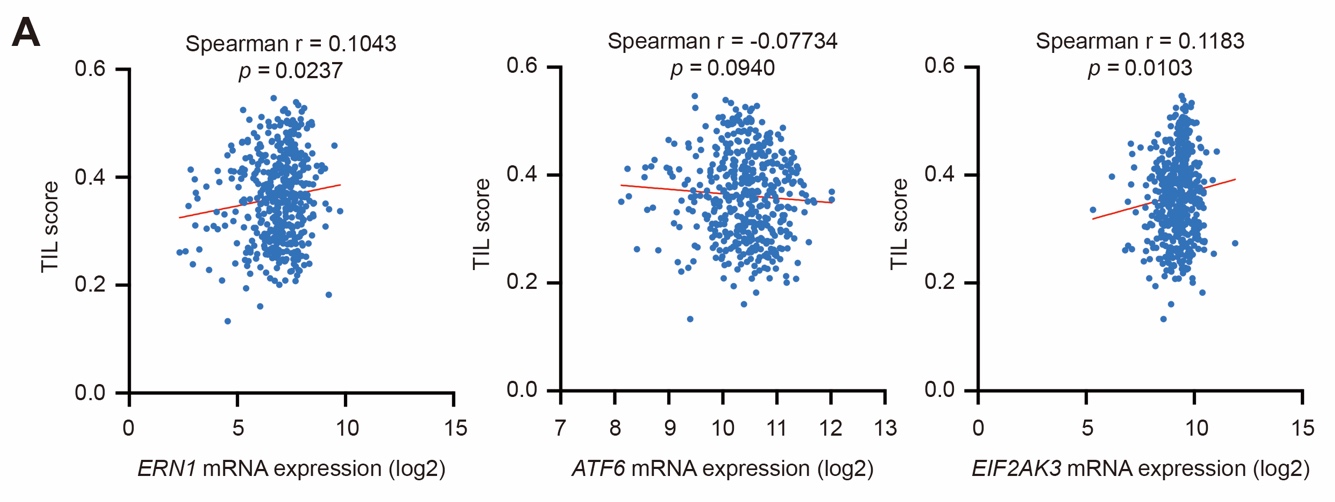
**

**Fig. S1** The expression of IRE1α, ATF6 nor PERK was not strongly associated with tumor-infiltrating lymphocytes score in melanoma. **A** Correlation analysis of IRE1α, ATF6 and PERK with tumor-infiltrating lymphocytes score in TCGA SKCM database. *r* value was calculated by Spearman correlation. *P* value was calculated by two tailed Student’s t-test.


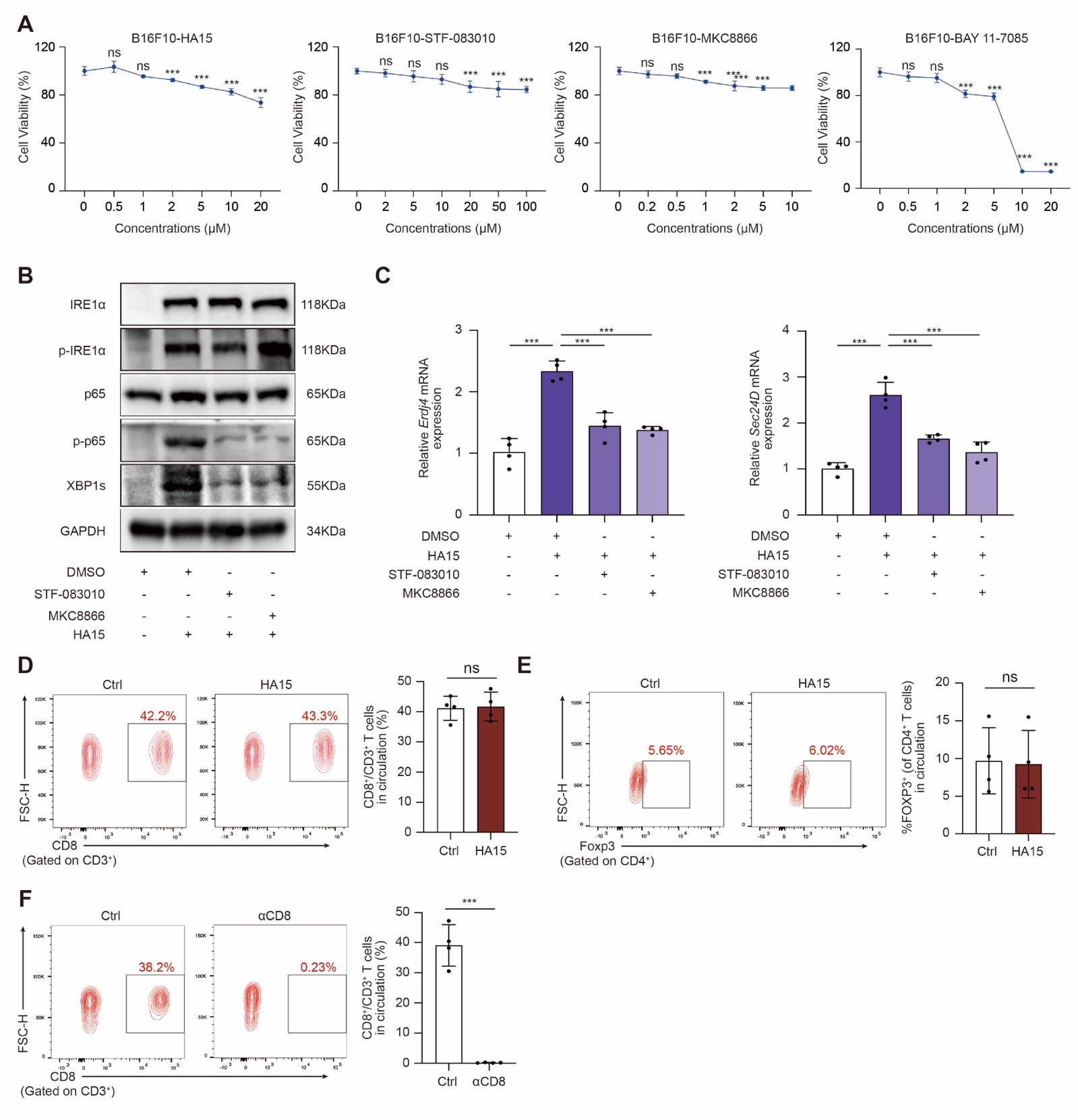


**Fig. S2** Tumorous IRE1α activates IRE1α-XBP1 and downstream NF-κB pathway in B16F10 melanoma cell line. **A** The relative cell viability at 24h of B16F10 melanoma cells after the treatment with HA15 of gradient concentrations (0, 0.5 μM, 1 μM, 2 μM, 5 μM, 10 μM, 20 μM), IRE1α inhibitor STF-083010 of gradient concentrations (0, 2 μM, 5 μM, 10 μM, 20 μM, 50 μM, 100 μM), IRE1α inhibitor MKC8866 of gradient concentrations (0, 0.2 μM, 0.5 μM, 1 μM, 2 μM, 5 μM, 10 μM), NF-κB inhibitor BAY 11-7085 of gradient concentrations (0, 0.5 μM, 1 μM, 2 μM, 5 μM, 10 μM, 20 μM) (n= 5). **B** Immunoblotting analysis of IRE1α, p-IRE1α, p65, p-p65, XBP1s and GAPDH expression in B16F10 cells treated with HA15 (10 μM) for 24h after pretreated with or without STF-083010 (10 μM) or MKC8866 (0.5 μM) for 24h. **C** Relative mRNA level of XBP transcriptional targets (*Erdj4*, *Sec24D*) in B16F10 cells treated with HA15 (10 μM) for 24h after pretreated with or without STF-083010 (10 μM) or MKC8866 (0.5 μM) for 24h (n= 4). **D** Representative flow cytometry data and summary plots of the frequency of CD8^+^ in peripheral blood from B16F10 xenografts received HA15 treatment as indicated (n= 4). **E** Representative flow cytometry data and summary plots of the frequency of Foxp3^+^CD4^+^ in peripheral blood from B16F10 xenografts received HA15 treatment as indicated (n= 4). **F** Representative flow cytometry data and summary plots of the frequency of CD8 staining in peripheral blood from B16F10 xenografts with CD8α antibody treatment to confirm depletion (n= 4). Data are representative of at least three independent experiments and shown as mean ± SD. One-way ANOVA or two-tailed Student’s t-test (**p* < 0.05; ***p* < 0.01; ****p* < 0.001; ns, not significant).


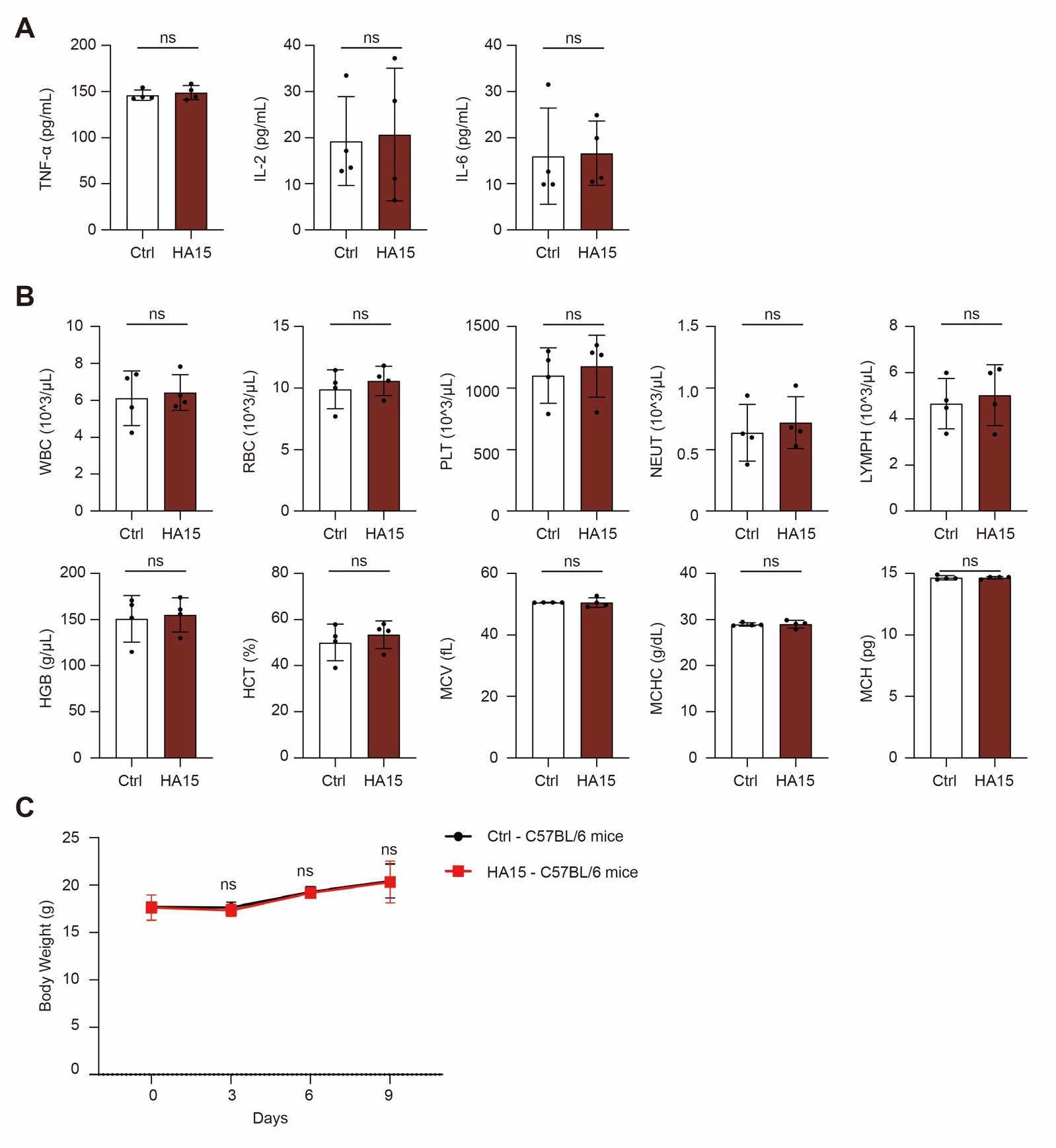


**Fig. S3** *In vivo* safety assessment of HA15 treatment in mice. **A** TNF-α, IL-2 and IL-6 levels in the serum of C57BL/6 mice bearing B16F10 tumors received HA15 treatment as indicated (n= 4). (B) The complete blood count (CBC) results after indicated treatment, including WBC, RBC, PLT, NEUT (neutrophil), LYMPH (lymphocyte), HGB (hemoglobin), HCT (Hematocrit), MCV (mean corpuscular volume), MCHC (mean corpuscular hemoglobin concentration) and MCH (mean corpuscular hemoglobin) (n= 4). (C) The body weights of the mice with indicated treatment as described in Figure 2A (n= 4). One-way ANOVA or two-tailed Student’s t-test (ns, not significant).


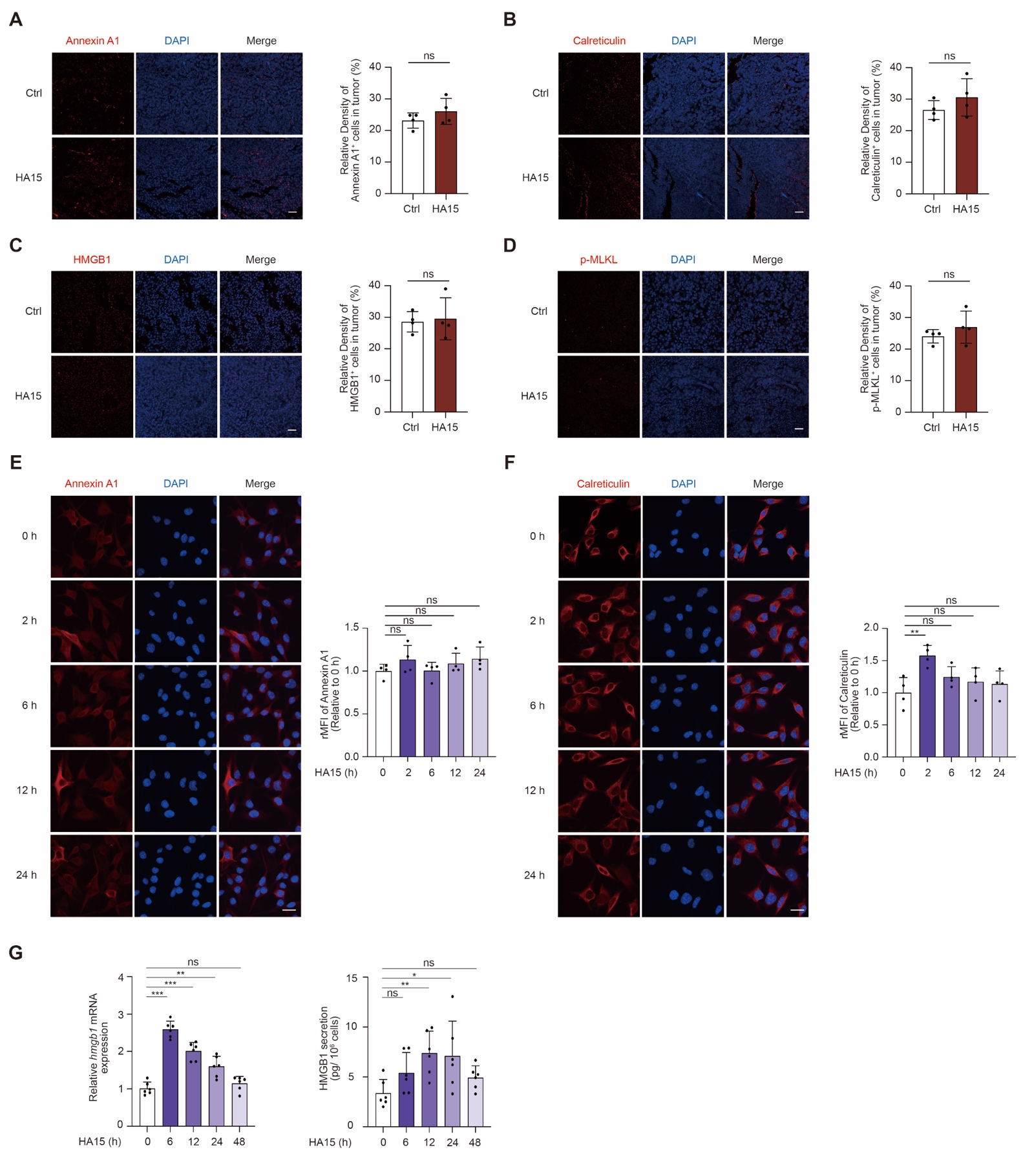


**Fig. S4** The improved anti-tumor effect of HA15 may not be associated with the induction of immunogenic or necroptotic cell death. Immunofluorescence staining of Annexin A1 (**A**), calreticulin (**B**), HMGB1 (**C**) and p-MLKL (**D**) in isolated transplanted tumors with indicated treatment. Scale bar= 30 μm. Immunofluorescence staining of Annexin A1(**E**) and calreticulin (**F**) in B16F10 cells with HA15 treatment (10 μM) in indicated time. Scale bar= 20 μm. (**G**) Relative mRNA level (n= 6) and ELISA (n= 6) analysis of HMGB1 in B16F10 cells treated with HA15 (10 μM) for indicated time. One-way ANOVA or two-tailed Student’s t-test (**p* < 0.05; ***p* < 0.01; ****p* < 0.001; ns, not significant)


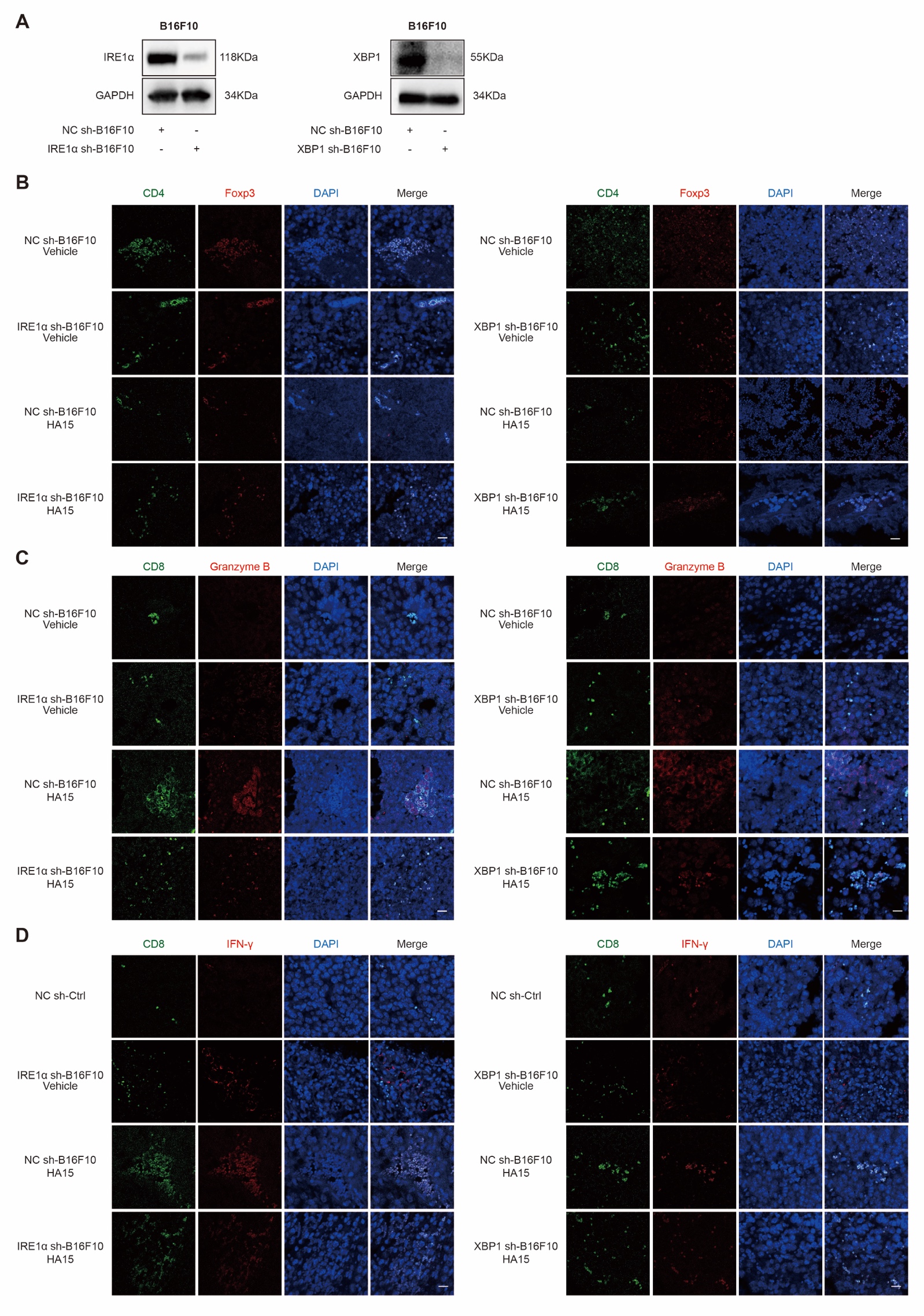


**Fig. S5** HA15 modulates the immune cell populations within the TME via IRE1a-XBP1 pathway. **A** Immunoblotting analysis of IRE1α or XBP1 in IRE1α shRNA Lentivirus transfected B16F10 cells or XBP1 shRNA Lentivirus transfected B16F10 cells. Immunofluorescence staining of Foxp3^+^CD4^+^T cells (**B**), Granzyme B^+^CD8^+^ T cells (**C**) and IFN-γ^+^CD8^+^ T cells (**D**) in isolated transplanted tumors with indicated treatment. Scale bar= 50 μm.


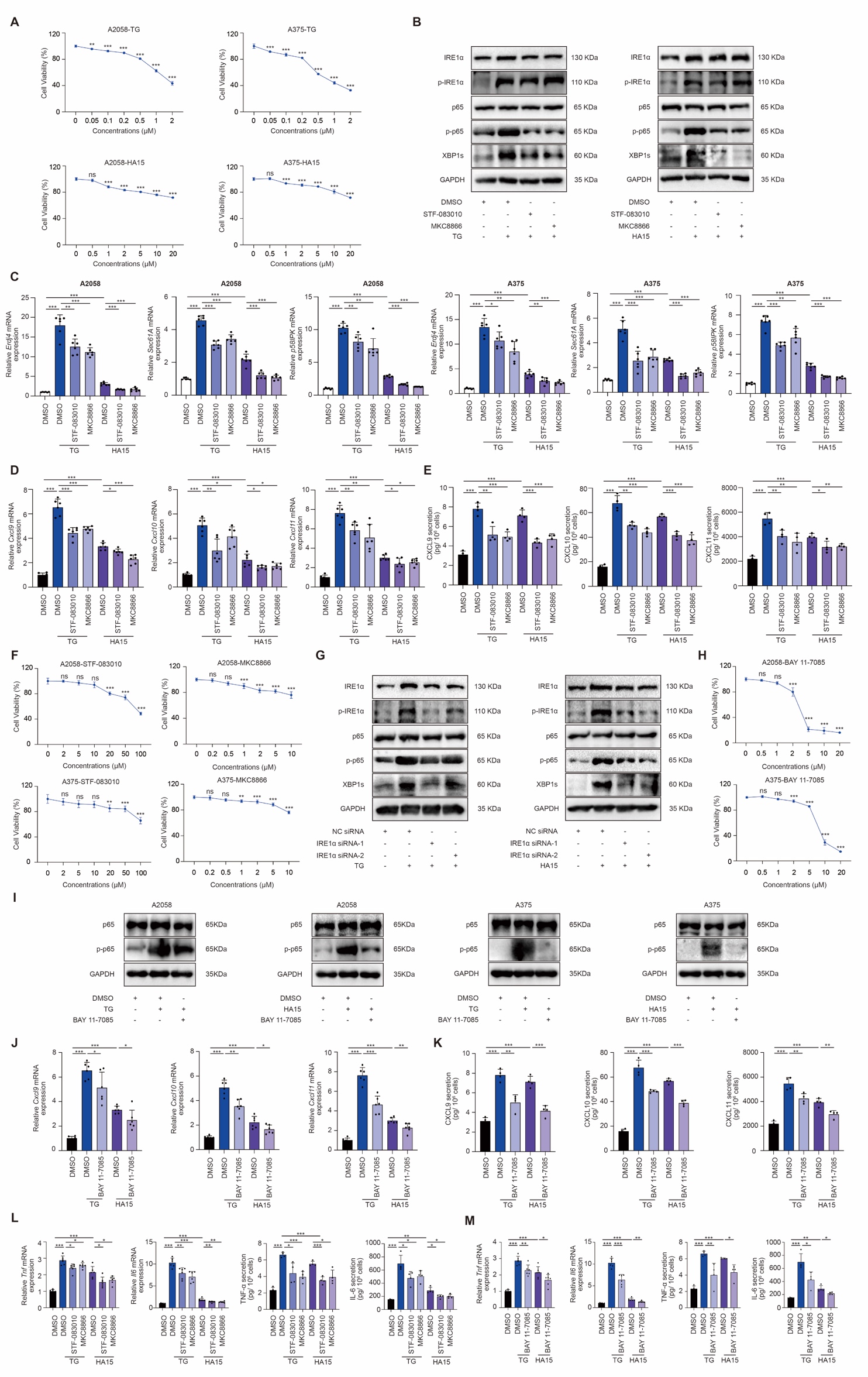


**Fig. S6** Tumorous IRE1α promotes the secretion of Th1-related chemokine and cytokines by activating IRE1α-NF-κB pathway. **A** The relative cell viability at 24h of A2058 and A375 melanoma cells after the treatment with TG of gradient concentrations (0, 0.05 μM, 0.1 μM, 0.2 μM, 0.5 μM, 1 μM, 2 μM) or HA15 of gradient concentrations (0, 0.5 μM, 1 μM, 2 μM, 5 μM, 10 μM, 20 μM) (n= 5). **B**, **G** Immunoblotting analysis of IRE1α, p-IRE1α, p65, p-p65, XBP1s and GAPDH in A375 cells treated with TG (0.2 μM) or HA15 (10 μM) for 24h after pretreated with or without STF-083010 (10 μM), MKC8866 (0.5 μM) or IRE1α siRNA for 24h. **C** Relative mRNA level of XBP transcriptional targets (*Erdj4*, *Sec61A, p58IPK*) in A2058 cells treated with TG (0.5 μM), HA15 (10 μM) or in A375 cells treated with TG (0.2 μM), HA15 (10 μM) for 24h after pretreated with or without STF-083010 (10 μM) or MKC8866 (0.5 μM) for 24h (n= 6). **D-E, J-M** Relative mRNA level (n= 6) and ELISA (n= 4) analysis of CXCL9, CXCL10, CXCL11, TNF-α and IL-6 in A375 cells treated with TG (0.2 μM) or HA15 (10 μM) for 24h after pretreated with or without STF-083010 (10 μM), MKC8866 (0.5 μM) or BAY 11-7085 (1 μM) for 24h. **F, H** The relative cell viability of A2058 and A375 melanoma cells after the treatment with IRE1α inhibitor STF-083010 of gradient concentrations (0, 2 μM, 5 μM, 10 μM, 20 μM, 50 μM, 100 μM), IRE1α inhibitor MKC8866 of gradient concentrations (0, 0.2 μM, 0.5 μM, 1 μM, 2 μM, 5 μM, 10 μM) or NF-κB inhibitor BAY 11-7085 of gradient concentrations (0, 0.5 μM, 1 μM, 2 μM, 5 μM, 10 μM, 20 μM) (n= 5) for 24 h. **I** Immunoblotting analysis of p65, p-p65 and GAPDH in A2058 cells treated with TG (0.5 μM), HA15 (10 μM) or in A375 cells treated with TG (0.2 μM) or HA15 (10 μM) for 24h after pretreated with or without BAY 11-7085 (1 μM) for 24h. Data are representative of at least three independent experiments and shown as mean ± SD. One-way ANOVA or two-tailed Student’s t-test (**p* < 0.05; ***p* < 0.01; ****p* < 0.001; ns, not significant).


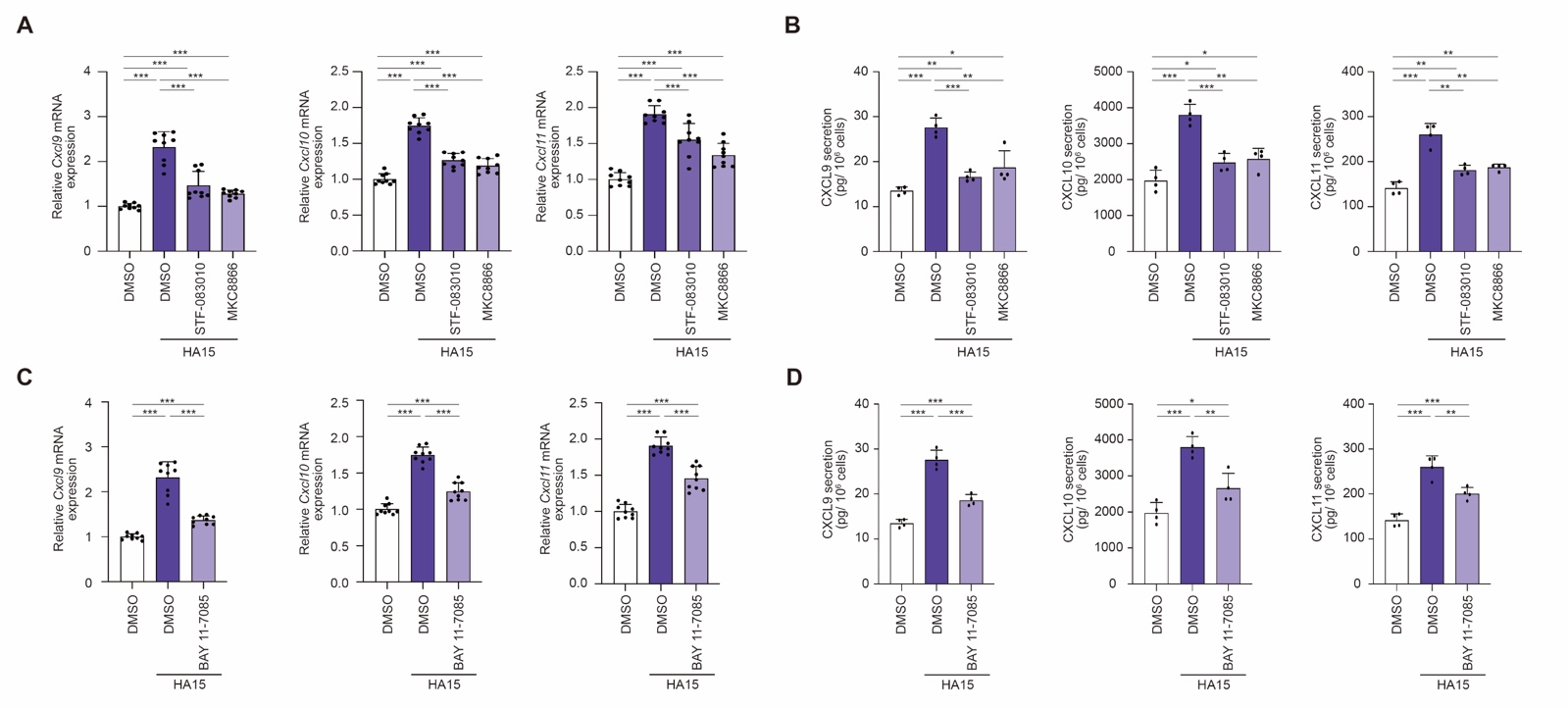


**Fig. S7** Tumorous IRE1α promotes the secretion of Th1-related chemokine by activating IRE1α-NF-κB pathway. **A-D** Relative mRNA level (n= 9) and ELISA (n= 4) analysis of CXCL9, CXCL10 and CXCL11 in B16F10 cells treated with HA15 (10 μM) for 24h after pretreated with or without STF-083010 (10 μM), MKC8866 (0.5 μM) or BAY 11-7085 (1 μM) for 24h. Data are representative of at least three independent experiments and shown as mean ± SD. One-way ANOVA or two-tailed Student’s t-test (**p* < 0.05; ***p* < 0.01; ****p* < 0.001).

**
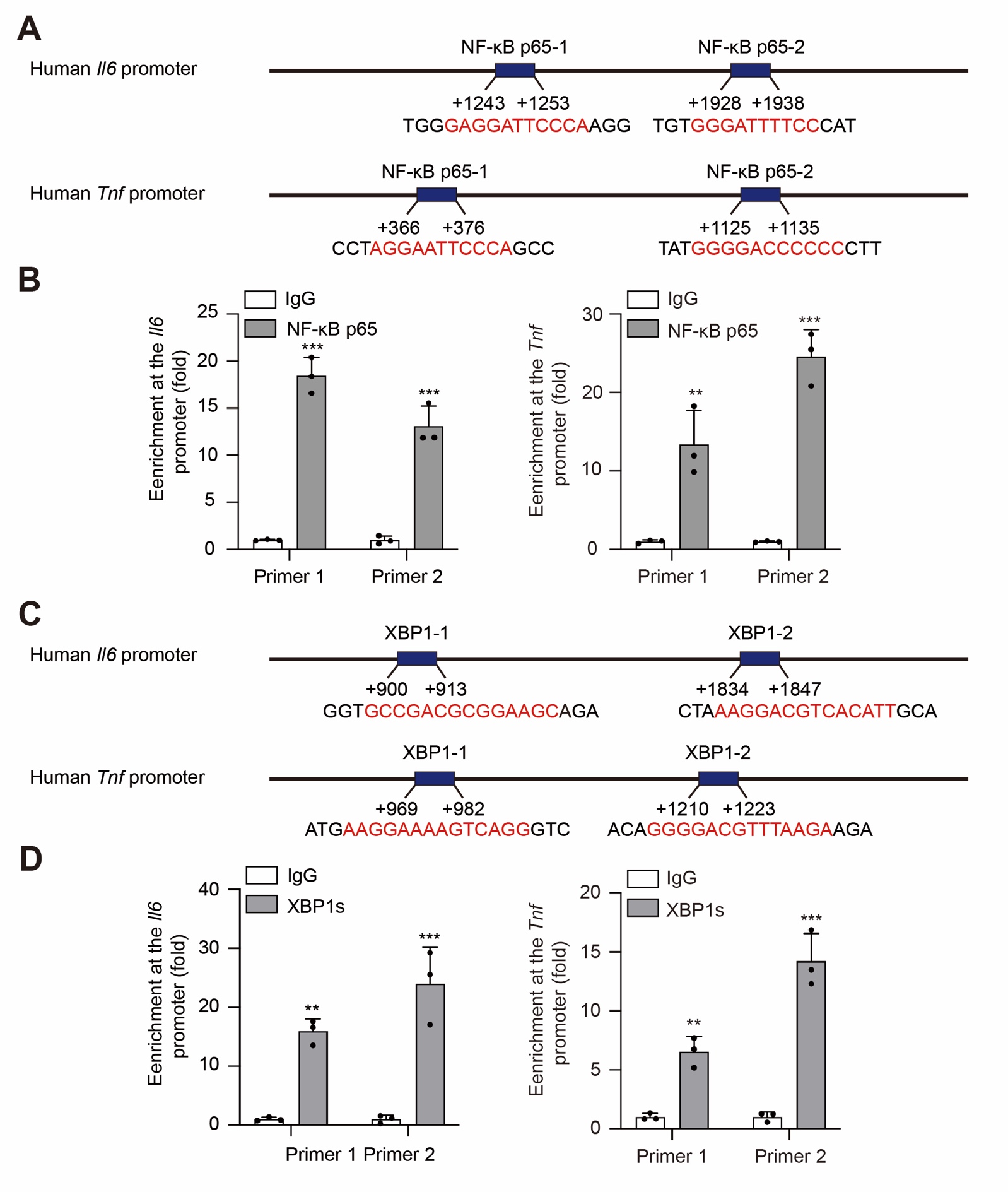
**

**Fig. S8** Recruitment of NF-κB and XBP1 to the *Il6* and *Tnf* promoters. **A** Schematic diagram of NF-κB binding regions in *Il6* promoter and *Tnf* promoter. **B** A2058 cells were subjected to ChIP with normal mouse IgG, NF-κB or Pol-II antibody as indicated (n= 3). **C** Schematic diagram of XBP1 binding regions in *Il6* promoter and *Tnf* promoter. **D** A2058 cells were subjected to ChIP with normal mouse IgG, XBP1 or Pol-II antibody as indicated (n= 3). ChIP samples were analyzed by qPCR using primers indicated in Additional file 2: Table 3. Data are representative of three independent experiments and shown as mean ± SD. Two-way ANOVA (**p* < 0.05; ***p* < 0.01; ****p* < 0.001).

**
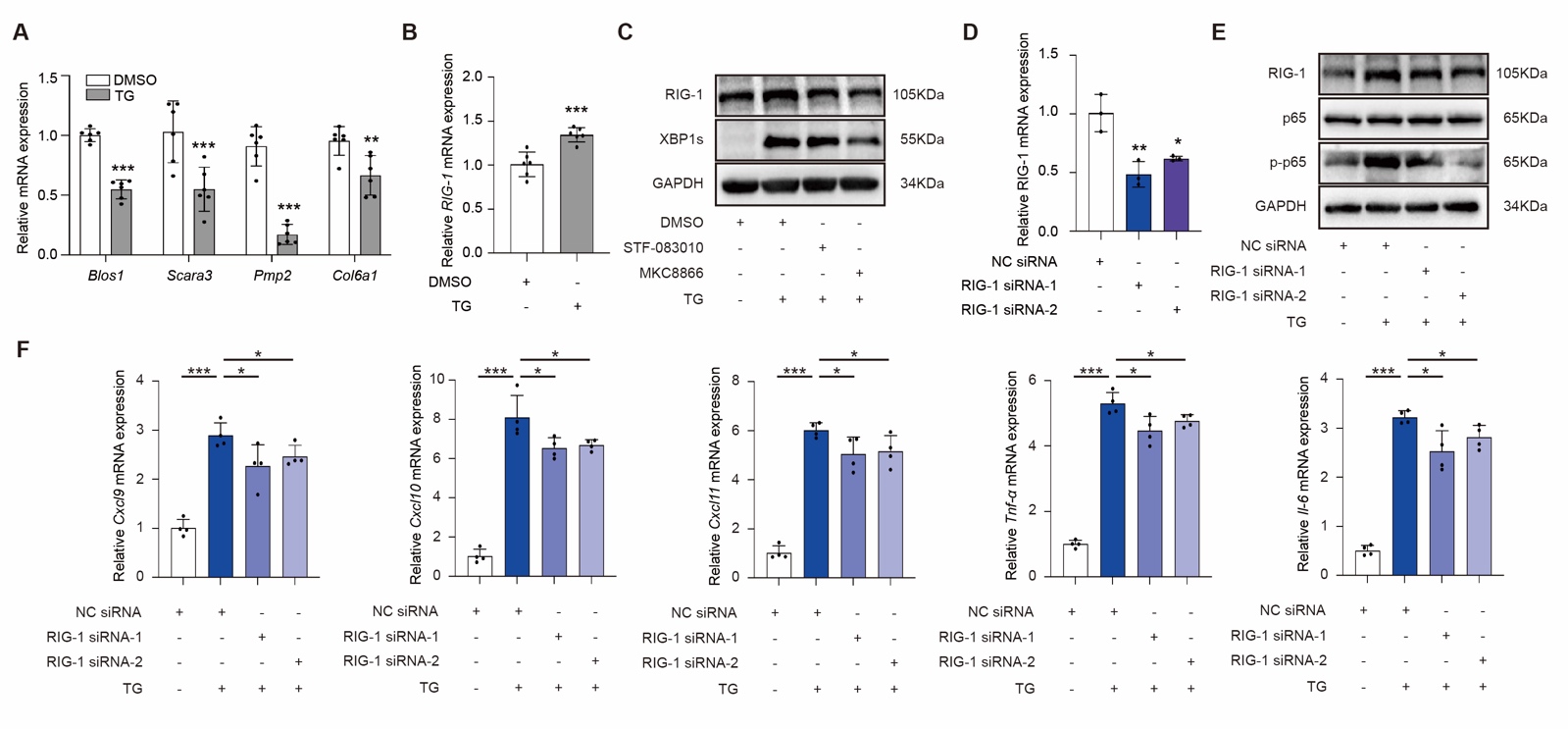
**

**Fig. S9** Tumorous IRE1α promotes the secretion of Th1-related chemokine and cytokines by activating IRE1α-RIDD-RIG-1 pathway. **A** Relative mRNA level of typical IRE1α RIDD targets (*Blos1, Scara3, Pmp2* and *Col6a1*) in A2058 cells treated with TG (0.5 μM) for 24h (n= 6). **B** Relative mRNA level of *RIG-1* in A2058 cells treated with TG (0.5 μM) for 24h (n= 6). **C** Immunoblotting analysis of RIG-1, XBP1s and GAPDH in A2058 cells treated with TG (0.5 μM) for 24h after pretreated with or without STF-083010 (10 μM) or MKC8866 (0.5 μM) for 24h. **D** Relative mRNA level of *RIG-1* in A2058 cells treated with RIG-1 siRNA for 24h (n= 3). **E** Immunoblotting analysis of RIG-1, p65, p-p65 and GAPDH in A2058 cells treated with TG (0.5 μM) for 24h after pretreated with or without RIG-1 siRNA for 24h. **F** Relative mRNA level of *Cxcl9*, *Cxcl10*, *Cxcl11*, *Tnf* and *Il-6* in A2058 cells treated with TG (0.5 μM) for 24h after pretreated with or without RIG-1 siRNA for 24h (n= 4). Data are representative of at least three independent experiments and shown as mean ± SD. Two-tailed Student’s t-test (**p* < 0.05; ***p* < 0.01; ****p* < 0.001).


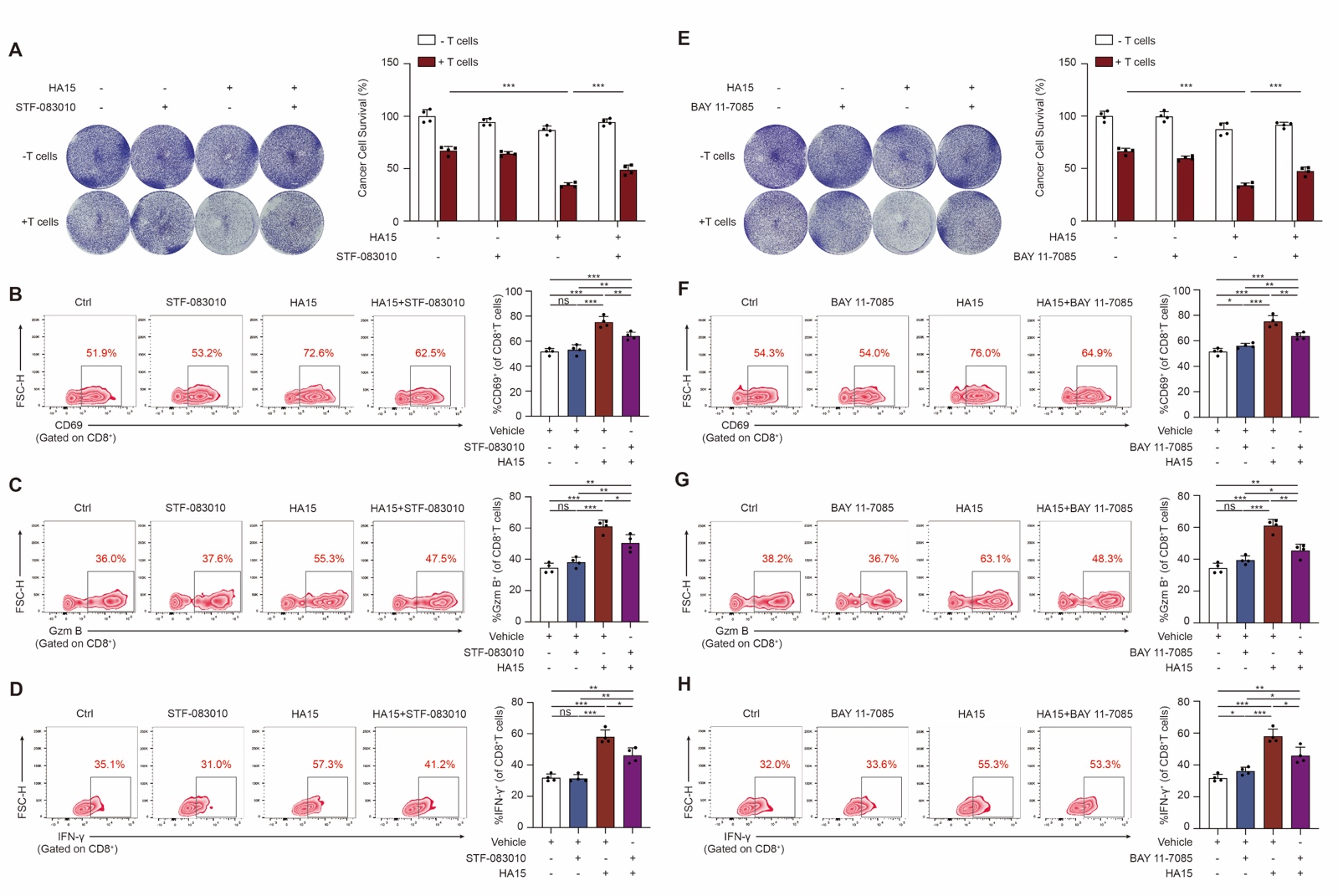


**Fig. S10** Tumorous IRE1α-dependent NF-κB activation contributes to the activation of CD8^+^T cells. **A, E** A375 melanoma cells treated with HA15 (10 μM) for 24h after pretreated with or without STF-083010 (10 μM) or BAY 11-7085 (1 μM) for 24h cocultured with or without activated T cell (1:3) for 24h were subjected to crystal violet staining. Cytotoxicity was quantified by a spectrometer at OD (570 nm) and normalized ratio of cancer cell survival was shown for each well (n= 4). **B-D, F-H** Representative flow cytometry data and summary plots of the frequency of CD8^+^ T cells evaluated for expression of CD69, Granzyme B and IFN-γ in co-culture system with indicated treatment (n= 4). Data are representative of four independent experiments and shown as mean ± SD. Two-tailed Student’s t-test (**p* < 0.05; ***p* < 0.01; ****p* < 0.001; ns, not significant).


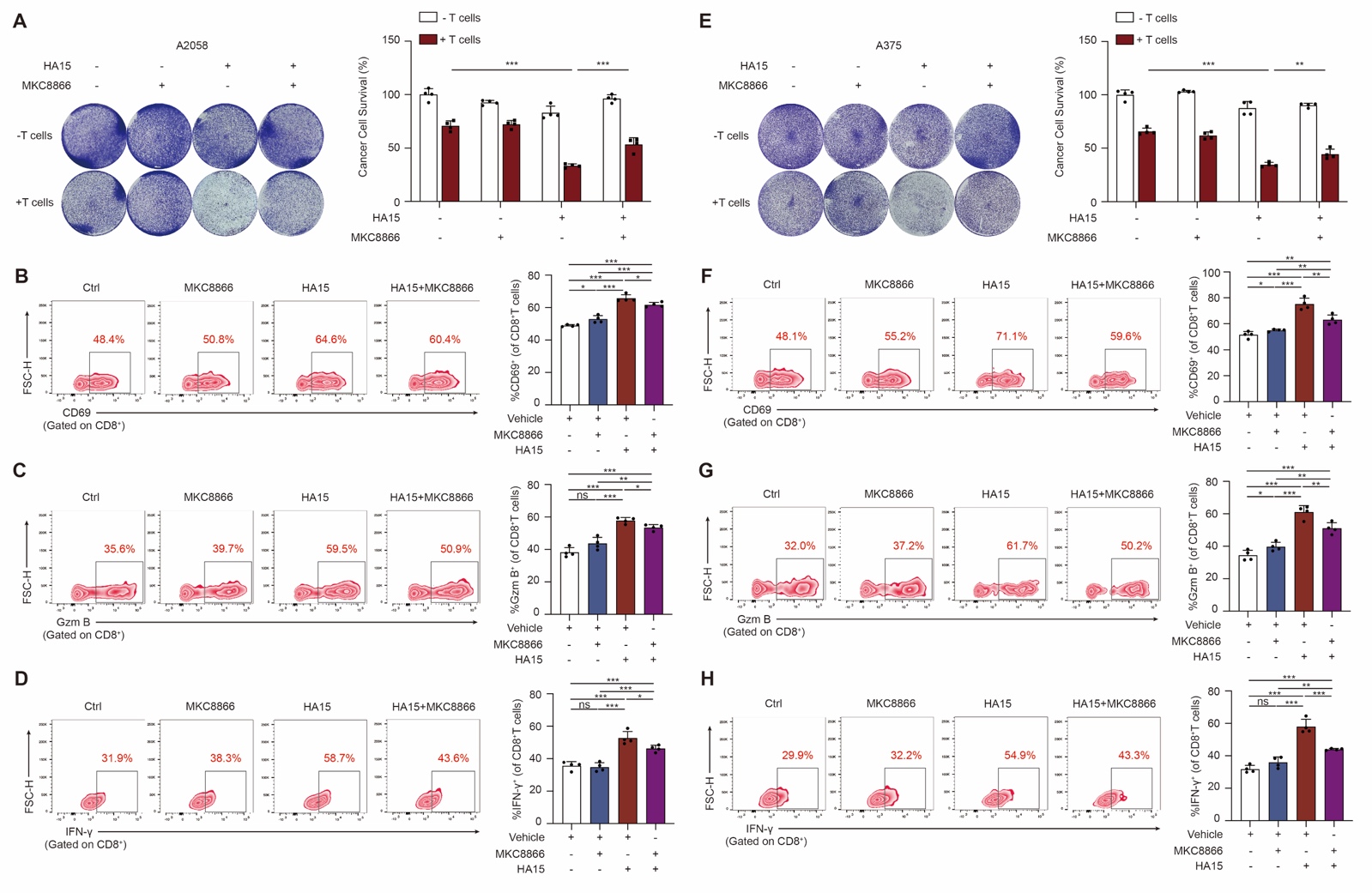


**Fig. S11** Tumorous IRE1α-dependent NF-κB activation contributes to the activation of CD8^+^T cells. **A, E** A2058 or A375 melanoma cells treated with HA15 (10 μM) for 24h after pretreated with or without MKC8866 (0.5 μM) for 24h cocultured with or without activated T cell (1:3) for 24h were subjected to crystal violet staining. Cytotoxicity was quantified by a spectrometer at OD (570 nm) and normalized ratio of cancer cell survival was shown for each well (n= 4). **B-D, F-H** Representative flow cytometry data and summary plots of the frequency of CD8^+^ T cells evaluated for expression of CD69, Granzyme B and IFN-γ in co-culture system with indicated treatment (n= 4). Data are representative of four independent experiments and shown as mean ± SD. Two-tailed Student’s t-test (**p* < 0.05; ***p* < 0.01; ****p* < 0.001; ns, not significant).


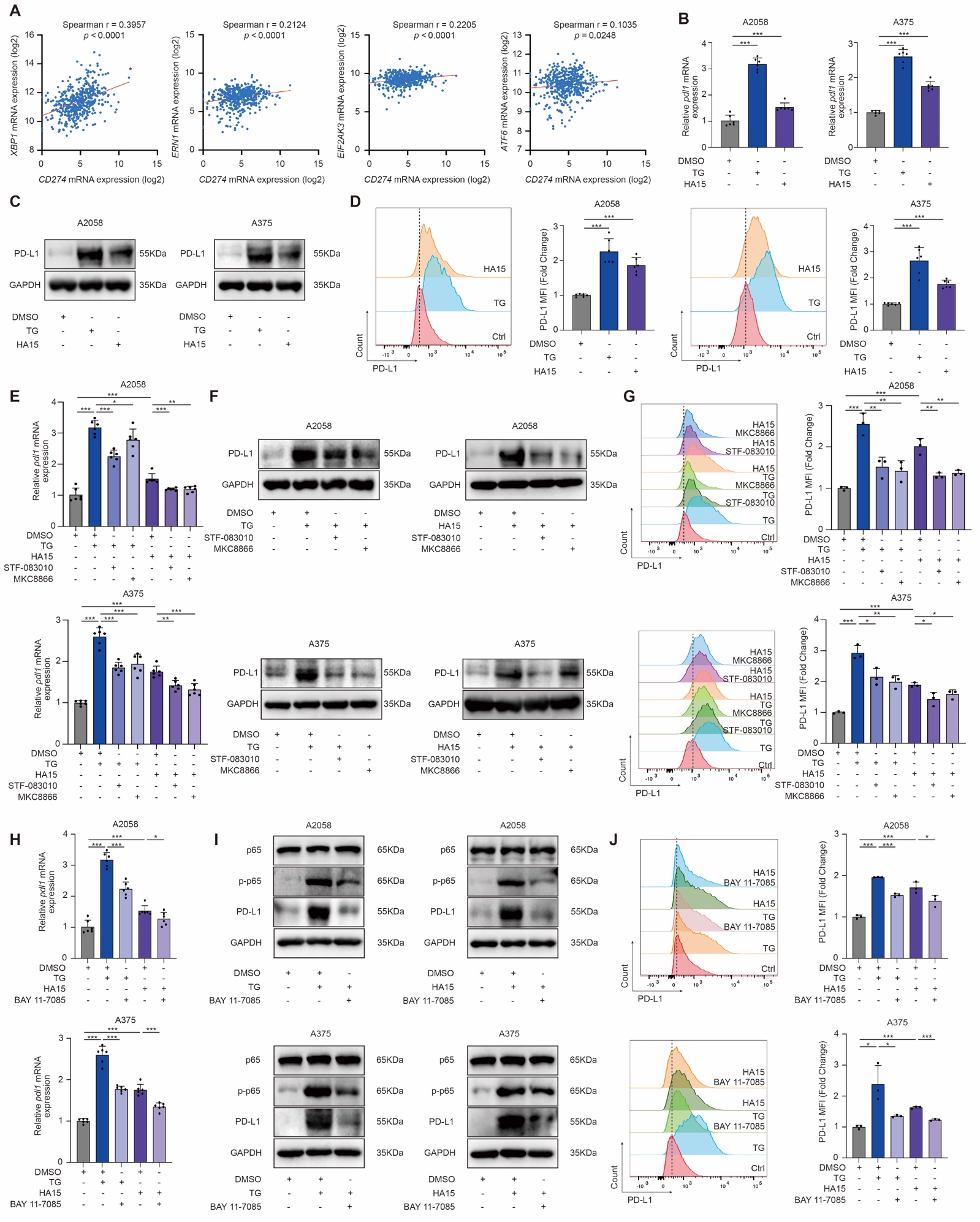


**Fig. S12** Tumorous IRE1α up-regulates PD-L1 expression via NF-κB pathway upon ER stress. **A** Correlation analysis of XBP1, IRE1α, ATF6 and PERK with PD-L1 expression in TCGA SKCM database. **B, E, H** Relative mRNA level of PD-L1 in A2058 cells treated with TG (0.5 μM), HA15 (10 μM) or A375 cells treated with TG (0.2 μM), HA15 (10 μM) for 24 h after pretreated with or without STF-083010 (10 μM), MKC8866 (0.5 μM) or BAY 11-7085 (1 μM) for 24 h (n= 6). **C, F** Immunoblotting analysis of PD-L1 expression in A2058 cells treated with TG (0.5 μM), HA15 (10 μM) or A375 cells treated with TG (0.2 μM), HA15 (10 μM) for 24 h after pretreated with or without STF-083010 (10 μM) or MKC8866 (0.5 μM) for 24 h. **D, G, J** The FACS analysis of relative membrane PD-L1 expression in A2058 cells treated with TG (0.5 μM), HA15 (10 μM) or A375 cells treated with TG (0.2 μM), HA15 (10 μM) for 24 h after pretreated with or without STF-083010 (10 μM), MKC8866 (0.5 μM) or BAY 11-7085 (1 μM) for 24 h (n= 3). **I** Immunoblotting analysis of p65, p-p65, PD-L1 and GAPDH expression in A2058 cells treated with TG (0.5 μM), HA15 (10 μM) or A375 cells treated with TG (0.2 μM), HA15 (10 μM) for 24 h after pretreated with or without BAY 11-7085 (1 μM) for 24 h. Data are representative of at least three independent experiments and shown as mean ± SD. Two-tailed Student’s t-test (**p* < 0.05; ***p* < 0.01; ***p < 0.001).
