# Supplementary material for: Tumorous IRE1α facilitates CD8+T cells-dependent anti-tumor immunity and improves immunotherapy efficacy in melanoma
Source: Cell Commun Signal. 2024 Jan 30;22:83. doi: 10.1186/s12964-024-01470-8 (PMC10826282; doi:10.1186/s12964-024-01470-8)
Supplement: Supplementary file 2 — Additional file 2: Table 1. Clinicopathologic characteristics of melanoma patient cohorts, related to Fig. 1. Table 2. Primary Antibodies Used. Table 3. Primers used for qPCR. [file 12964_2024_1470_MOESM2_ESM.docx]

| **Table 1. Clinicopathologic characteristics of melanoma patient cohorts, related to Figure 1.** | | | | | |
| --- | --- | --- | --- | --- | --- |
|  |  |  |  |  |  |
| **Patient No.** | **Gender** | **Age** | **AJCC stage** | **Breslow thickness** | **Ulceration** |
| 1 | Female | 73 | IIID | ＞4 mm | Positive |
| 2 | Female | 69 | IIB | 2-4 mm | Positive |
| 3 | Male | 69 | IIB | 1-2 mm | Positive |
| 4 | Female | 58 | IIA | 1-2 mm | Positive |
| 5 | Male | 59 | IVB | ＞4 mm | Positive |
| 6 | Female | 53 | IIIC | ＞4 mm | Positive |
| 7 | Female | 20 | IIC | ≤1 mm | Negative |
| 8 | Female | 66 | IIB | 2-4 mm | Positive |
| 9 | Female | 59 | IIIC | 2-4 mm | Positive |
| 10 | Female | 61 | IIIC | 2-4 mm | Negative |
| 11 | Male | 49 | IIB | 2-4 mm | Positive |
| 12 | Male | 74 | IIIC | ＞4 mm | Positive |
| 13 | Male | 61 | IIIC | ＞4 mm | Positive |
| 14 | Female | 52 | IIIC | ＞4 mm | Negative |
| 15 | Male | 73 | IIIC | ＞4 mm | Positive |
| 16 | Male | 62 | IVB | ＞4 mm | Positive |
| 17 | Male | 53 | IIC | ＞4 mm | Positive |
| 18 | Male | 49 | IIIB | 2-4 mm | Positive |
| 19 | Male | 78 | IIC | ＞4 mm | Positive |
| 20 | Male | 65 | IIC | ＞4 mm | Positive |
| 21 | Male | 36 | II | 2-4 mm | Positive |
| 22 | Male | 69 | IIB | ＞4 mm | Positive |
| 23 | Female | 64 | II | 2-4 mm | Positive |
| 24 | Male | 50 | II | ＞4 mm | Positive |
| 25 | Female | 69 | IV | 2-4 mm | Positive |
| 26 | Male | 72 | II | ＞4 mm | Positive |
| 27 | Male | 74 | IIC | ＞4 mm | Positive |
| 28 | Male | 69 | IIC | 2.25mm | Positive |
| 29 | Male | 62 | IIC | ≤1 mm | Positive |
| 30 | Female | 82 | IIC | 2-4 mm | Positive |
| 31 | Female | 63 | III | ＞4 mm | Positive |

| **Table 2. Primary Antibodies Used for Immunoblotting, Immunostaining, ChIP and FACS** | | |
| --- | --- | --- |
|  | | |
| **Antibody** | **Company (Cat. No.)** | **Dilutions** |
| rabbit anti-NF-κB p65 monoclonal antibody | CST, 8242S | WB: 1/ 1,000; ChIP: 1/ 100 |
| rabbit anti-Phospho-NF-κB p65 monoclonal antibody | CST, 3033S | WB: 1/ 1,000 |
| rabbit anti-PD-L1 monoclonal antibody | CST, 13684S | WB: 1/ 1,000 |
| rabbit anti-IRE1α monoclonal antibody | CST, 3294S | WB: 1/ 1,000 |
| rabbit anti-IRE1 (phospho S724) polyclonal antibody | Novus, NBP100-2323 | WB: 1/ 1,000 |
| mouse anti-XBP1s monoclonal antibody | CST, 27901 | ChIP: 1/ 100 |
| rabbit anti-XBP1s antibody | CST, 12782 | WB: 1/ 1,000 |
| rabbit anti-XBP1u polyclonal antibody | Proteintech, 25997-1-AP | IHC: 1/ 100 |
| rabbit anti-XBP1s polyclonal antibody | Proteintech, 24868-1-AP | WB: 1/ 1,000; IF: 1/ 100 |
| mouse anti-GAPDH monoclonal antibody | Proteintech, 60004-1-Ig | WB: 1/ 5,000 |
| rat anti-CD8 alpha monoclonal antibody | Abcam, ab22378 | IF: 1/ 200 |
| rabbit anti-CD8α antibody | ZSGB-BIO (ZA-0508) | IHC: 1/ 1 |
| rabbit anti-XBP1 polyclonal antibody | Novus (NBP1-77681) | IHC: 1/ 100 |
| rabbit anti-IFN-γ antibody | Biosynthesis Biotechnology, bs-0480R | IHC: 1/ 100 |
| rabbit anti-Phospho- MLKL (Ser345) (D6E3G) monoclonal antibody | CST, 37333 | IF: 1/ 100 |
| rabbit anti-HMGB1 polyclonal antibody | Proteintech, 10829-1-AP | IF: 1/ 100 |
| rabbit anti-calreticulin polyclonal antibody | Proteintech, 27298-1-AP | IF: 1/ 100 |
| rabbit anti-Annexin A1 polyclonal antibody | Proteintech, 21990-1-AP | IF: 1/ 100 |
| CoraLite® Plus 488 Anti-Mouse CD4 (RM4-5) | Proteintech, CL488-65141 | IF: 1/ 100 |
| Anti-Mouse Foxp3 (3G3) | Proteintech, 65089-1-Ig | IF: 1/ 100 |
| Anti-Mouse CD8a (53-6.7) | Proteintech, 65069-1-Ig | IF: 1/ 100 |
| rabbit anti-IFN-γ polyclonal antibody | SinoBiological, 105995-T08 | IF: 1/ 100 |
| rabbit anti-Granzyme B polyclonal antibody | Proteintech, 13588-1-AP | IF: 1/ 100 |
| anti-mouse Fc blocking Ab | Biolegend, 101320 | FACS: 1.0 µg per 10^6^ cells |
| Pacific Blue-conjugated anti-mouse CD45 | Biolegend, 103126 | FACS: 0.25 µg per 10^6^ cells |
| APC-conjugated anti-mouse CD3 | Biolegend, 100236 | FACS: 0.5 µg per 10^6^ cells |
| PE/Cyanine7-conjugated anti-mouse CD8α | Biolegend, 100722 | FACS: 0.25 µg per 10^6^ cells |
| BV650-conjugated anti-mouse CD11c | Biolegend, 117339 | FACS: 0.25 µg per 10^6^ cells |
| APC-conjugated anti-mouse CD4 | Biolegend, 100412 | FACS: 0.25 µg per 10^6^ cells |
| PE/Cyanine7-conjugated anti-mouse CD25 | Biolegend, 102016 | FACS: 0.25 µg per 10^6^ cells |
| APC-conjugated anti-mouse F4/80 | Biolegend, 123116 | FACS: 0.25 µg per 10^6^ cells |
| PE-conjugated anti-mouse/human CD11b | Biolegend, 101208 | FACS: 0.25 µg per 10^6^ cells |
| BV605-conjugated anti-mouse IFN-γ | Biolegend, 505840 | FACS: 1.0 µg per 10^6^ cells |
| PE-conjugated anti-human/ mouse Granzyme B | Biolegend, 372208 | FACS: 1.0 µg per 10^6^ cells |
| PE-conjugated anti-mouse Foxp3 | Biolegend, 126404 | FACS: 1.0 µg per 10^6^ cells |
| APC-conjugated anti-human CD3 | Biolegend, 300312 | FACS: 5.0 µL per 10^6^ cells |
| PE/Cyanine7-conjugated anti-human CD8α | Biolegend, 344750 | FACS: 0.25 µg per 10^6^ cells |
| FITC-conjugated anti-human CD69 | Biolegend, 310904 | FACS: 5.0 µL per 10^6^ cells |
| PE-conjugated anti-human IFN-γ | Biolegend, 502522 | FACS: 0.25 µg per 10^6^ cells |

| **Table 3. Primers used for qRT-PCR** | |  |
| --- | --- | --- |
|  |  |  |
| **Genes** | **Forward (5'-3')** | **Reverse (5'-3')** |
| *Ifng* | TCGGTAACTGACTTGAATGTCCA | TCGCTTCCCTGTTTTAGCTGC |
| *Tnf* | GAGGACCTGGGAGTAGATGAG | ACTCACCTCTTCAGAACGAATTG |
| *Il6* | ACTCACCTCTTCAGAACGAATTG | CCATCTTTGGAAGGTTCAGGTTG |
| *Cxcl9*  (human) | CCAGTAGTGAGAAAGGGTCGC | AGGGCTTGGGGCAAATTGTT |
| *Cxcl10*  (human) | GTGGCATTCAAGGAGTACCTC | TGATGGCCTTCGATTCTGGATT |
| *Cxcl11*  (human) | GACGCTGTCTTTGCATAGGC | GGATTTAGGCATCGTTGTCCTTT |
| *PD-L1* | TGGCATTTGCTGAACGCATTT | TGCAGCCAGGTCTAATTGTTTT |
| *Erdj4* | TCGGCATCAGAGCGCCAAATCA | ACCACTAGTAAAAGCACTGTGTCCAAG |
| *Sec61A1* | TGTCATCTCCCAAATGCTCTCA | ACAGGTAATAGCAAAGGCCAC |
| *p58IPK* | TGTGTTTGGGATGCAGAACTAC | TCTTCAACTTTGACGCAGCTT |
| *Gapdh* | TGGTATCGTGGAAGGACTC | AGTAGAGGCAGGGATGATG |
| *Erdj4* (mouse) | CTTAGGTGTGCCAAAGTCTGC | GGCATCCGAGAGTGTTTCATA |
| *Sec24D* (mouse) | GGAGAGGTCTTTGTTCCTTTGTT | GTCTCTGTTCTTGAGCTTCCC |
| *Gapdh* (mouse) | AGGAGAGTGTTTCCTCGTCC | TGCCGTGAGTGGAGTCATAC |
| *Cxcl9*  (mouse) | TCCTTTTGGGCATCATCTTCC | TTTGTAGTGGATCGTGCCTCG |
| *Cxcl10*  (mouse) | CCAAGTGCTGCCGTCATTTTC | GGCTCGCAGGGATGATTTCAA |
| *Cxcl11*  (mouse) | GGCTTCCTTATGTTCAAACAGGG | GCCGTTACTCGGGTAAATTACA |
| *Hmgb1*  (mouse) | GCTGACAAGGCTCGTTATGAA | CCTTTGATTTTGGGGCGGTA |
| *Il6-1* (XBP1) | CACCATCCTGAGGGAAGAGG | TGGGGATGTCAAAGGAGGAC |
| *Il6-2* (XBP1) | TGTCAAGACATGCCAAAGTGC | GACTCATGGGAAAATCCCACA |
| *Il6-1* (NF-κB) | CCCCAGTGAAACAGTGGTGA | CGTCTCCAGGTGGAGTGTGT |
| *Il6-2* (NF-κB) | GCTAGCCTCAATGACGACCTAAG | TGAGCCTCAGACATCTCCAGTC |
| *Tnf-1* (XBP1) | GCAAGAGCTGTGGGGAGAAC | CCATTCCTCAGAGCCGCTAC |
| *Tnf-2* (XBP1) | TGACCACAGCAATGGGTAGG | TGGCTTCCAAGGAACTCTGG |
| *Tnf-1* (NF-κB) | CACCTCTCCTTTGGCCATTC | GTCCCCTCCCTGCCTCTAGT |
| *Tnf-2* (NF-κB) | CACATGTAGCGGCTCTGAGG | CCCAGTGTGTGGCCATATCTT |
