# Supplementary material for: Tumorous IRE1α facilitates CD8+T cells-dependent anti-tumor immunity and improves immunotherapy efficacy in melanoma
Source: Cell Commun Signal. 2024 Jan 30;22:83. doi: 10.1186/s12964-024-01470-8 (PMC10826282; doi:10.1186/s12964-024-01470-8)
Supplement: Supplementary file 3 — Additional file 3. Supplementary Materials and methods. [file 12964_2024_1470_MOESM3_ESM.docx]

**Additional file 3**

**Supplementary Materials and methods**

**Clinical samples**

31 melanoma specimens for immunohistochemistry were collected at Department of Dermatology, Xijing Hospital, The Fourth Military Medical University between August 2013 and April 2022. Tumor sections were reviewed by pathologists at Xijing Hospital according to the AJCC classification. The clinicopathological information, including age, sex, AJCC stage, breslow thickness and ulceration are summarized in Table 1. Normal blood samples for co-culture and apoptosis experiment were collected from healthy volunteers working at our department (two man and two women, age ranged from 24-39 years with mean of 29.75 years). All subjects gave written informed consent according to the Declaration of Helsinki, and approval from the Clinical Research Ethics Committee of Xijing Hospital, Fourth Military Medical University.

**Bioinformatic Analysis**

A total of 456 melanoma samples with complete survival information were collected from TCGA database (https://www.cancer.gov/), and the mRNA transcriptome data were transformed into FPKM format for further analysis. The ssGSEA algorithm was used to evaluate the enrichment score of IRE1α activity and tumor-infiltrating lymphocytes (TIL) [1] in each melanoma sample via “GSVA” package [2]. The optimal cutoff values of IRE1α activity and TIL were selected by the “surv_cutpoint” function of “survival” package [3]. Patients were clustered according to IRE1α activity based on the normalized z-score of gene expression for the BioInfoMiner signature of 38 genes [4]. The z-score was calculated by the equation (X - m)/s, X stands for normalized log2 expression data of each gene in each sample; m stands for mean of expression of each gene among all samples; and s stands for standard deviation. This 38-genes signature was used to stratify melanoma (TCGA) into IRE1α high and IRE1α low activity tumors. Then based on these 2 tumor groups, the expression of the following T-cell markers was evaluated in the two groups using the transcriptome data: IFNG, IRF1, STAT1, GZMB, PRF1, CXCL9, CXCL10, CXCL11, CXCR3, HLA-DPA1 and HLA-DPB1.The TCGA melanoma samples were used to estimate the correlation between XBP1, CD274, ERN1, EIF2AK3, ATF6, PTPRC, CD8A, Granzyme B, IFN-γ, and XBP1 target genes, including DNAJB9, ERDJ4 gene expression by spearman correlation analysis and presented in the scatter diagrams.

**Immunohistochemistry (IHC)**

For the analysis of CD8α and XBP1 in paraffin-embedded melanoma tissues from patients, tissue sections were de-paraffinized and rehydrated with graded ethanol dilutions. After antigen retrieval in Tris-EDTA buffer (10 mM Tris Base, 1 mM EDTA solution, 0.05% Tween-20, pH 9.0), goat serum was added to block non-specific binding for 30 min. Tissue sections were then incubated with rabbit anti-CD8α antibody (1:1, ZA-0508, ZSGB-BIO, China), or rabbit anti-XBP1 polyclonal antibody (1:100, NBP1-77681, Novus Biologicals) at 4°C overnight, followed by horseradish peroxidase‒ conjugated goat anti-rabbit/mouse IgG (1:200, Earthox, Millbrae, CA) for 40 minutes at room temperature. The section was then incubated in 3-amino-9- ethylcarbazole, and subsequently counterstained with hematoxylin and mounted with glycerol. The final staining score was read by two pathologists and calculated as described previously [5]. The proportion was scored into four categories: 0 (0%), 1 (1%–33%), 2 (34%–66%) or 3 (67%–100%). The intensity was scored into four grades: 0 (none staining), 1 (weak staining), 2 (moderate staining) or 3 (strong staining). The final staining score was defined as the product of the percentage and the intensity scores.

**Lentiviral vectors and siRNA transfection**

For the knockdown of IRE1α and XBP1 expression, the lentiviral vectors encoding shRNAs against IRE1α and XBP1 were used to infect murine melanoma cells according to the manufacturer's protocol. After 72 hours of infection, puromycin (10 μg/mL) (Beyotime, China) was used to remove the cells with no infection efficiency and to obtain stably infected clones. For the transient knockdown of IRE1α and RIG-1, siRNAs against *IRE1α* and *Rig-1* were used to transfect human melanoma cells according to the manufacturer-recommended procedures of Lipofectamine 3000 (Invitrogen). Lentiviral vectors and siRNA were purchased from Tsingke, Beijing. The sequences of shRNAs are as follows: sh-mIRE1α: 5′- GCTCGTGAATTGATAGAGAAA-3′; sh-mXBP1: 5′- ACCACAAACTCCAGCTAGAAA-3′. The sequences of siRNAs against IRE1α and RIG-1 are as follows: si-h*IRE1α*-1: 5′- CCCAUCAACCUCUCUUCUGUA-3′; si-h*IRE1α*-2: 5′- CAUCGUUCACAGAGACCUA-3′; si-h*RIG-1*-1: 5′- CAGAAGAUCUUGAGGAUAA-3′; si-h*RIG-1*-2: 5′- CCUUCAGAAGUGUCUGAUA-3′.

**Cell viability assay**

Cell viability was evaluated using the cell counting kit-8 assay (7seabiotech, Shanghai, China) according to the manufacturer’s instructions. Briefly, cells were seeded in 96-well plates (7,000 cells per well) and treated with the indicated stimulations for 24 hours. The culture medium was replaced with a fresh medium containing the CCK-8 reagent (1:10) for each well, and the plate was incubated for 1 h at 37 °C. The absorbance at 450 nm was measured using a Model 680 Microplate Reader (Bio-Rad Laboratories, Hercules, CA).

**hTF (Transcription factor) target analysis**

Promoter region sequences for human *Il6* and *Tnf* were obtained via https://www.ncbi.nlm.nih.gov/gene/. The binding sites for transcription factor XBP1 to *Il6* promoter, transcription factor XBP1 to *Tnf* promoter, transcription factor NF-κB p65 to *Il6* promoter and transcription factor NF-κB p65 to *Tnf* promoter were predicted by JASPAR databases. According to the predicted scores, the higher scoring binding sites (Figure S3c, S3e) was chosen for validation by Chromatin immunoprecipitation (ChIP) assay, the most significantly enriched sites were used for real time PCR analysis.

**Chromatin immunoprecipitation (ChIP)**

ChIP assays were performed with EZ-Magna ChIP^TM^ A/G Chromatin Immunoprecipitation Kit (17-10086, Millipore Corp) as described in manufacturer’s instructions. Briefly, cells were crosslinked with 1% formaldehyde. After cell lysis, isolated nuclei were subjected to sonication for chromatin fragmentation. The sheared chromatin was immunoprecipitated at 4°C overnight with rotation using anti-XBP1s antibody (1:100, #27901, Cell Signaling) or anti-NF-κB antibody (1:100, #8242, Cell Signaling). Normal mouse IgG was used as the negative control, and anti-RNA Polymerase II (Millipore) was used as the positive control. Antibody-chromatin complexes were captured using magnetic protein A/G beads. Purified DNAs were subjected to quantitative PCR (qPCR). Primers for ChIP-qPCR are listed in Supplementary Table 3. % Input was calculated as follows: 100% × 2^(–△CT)^, and △CT = Ct (Ip) - Ct (Input).

**Hematological Assessment**

Hematological analysis of blood was carried out using a multispecies hematological analyzer (Sysmex XN-1000V, Kobe, Japan) using manufacturer’s protocol for the following parameters: WBC (white blood cell), RBC (red blood cell), PLT (platelet), NEUT (neutrophil), LYMPH (lymphocyte), HGB (hemoglobin), HCT (Hematocrit), MCV (mean corpuscular volume), MCHC (mean corpuscular hemoglobin concentration) and MCH (mean corpuscular hemoglobin).

**References**

1. Lucca LE, Axisa PP, Lu B, Harnett B, Jessel S, Zhang L*, et al.* Circulating clonally expanded T cells reflect functions of tumor-infiltrating T cells. J Exp Med. 2021;218.

2. Hanzelmann S, Castelo R & Guinney J. GSVA: gene set variation analysis for microarray and RNA-seq data. BMC Bioinformatics. 2013;14,7.

3. Wang XX, Wu LH, Dou QY, Ai L, Lu Y, Deng SZ*, et al.* Construction of m6A-based prognosis signature and prediction for immune and anti-angiogenic response. Front Mol Biosci. 2022;9,1034928.

4. Lhomond S, Avril T, Dejeans N, Voutetakis K, Doultsinos D, McMahon M*, et al.* Dual IRE1 RNase functions dictate glioblastoma development. EMBO Mol Med. 2018;10.

5. Yi X, Wang H, Yang Y, Wang H, Zhang H, Guo S*, et al.* SIRT7 orchestrates melanoma progression by simultaneously promoting cell survival and immune evasion via UPR activation. Signal Transduct Target Ther. 2023;8,107.
