# Supplementary material for: Tumorous IRE1α facilitates CD8+T cells-dependent anti-tumor immunity and improves immunotherapy efficacy in melanoma
Source: Cell Commun Signal. 2024 Jan 30;22:83. doi: 10.1186/s12964-024-01470-8 (PMC10826282; doi:10.1186/s12964-024-01470-8)

Fig. 4A- left

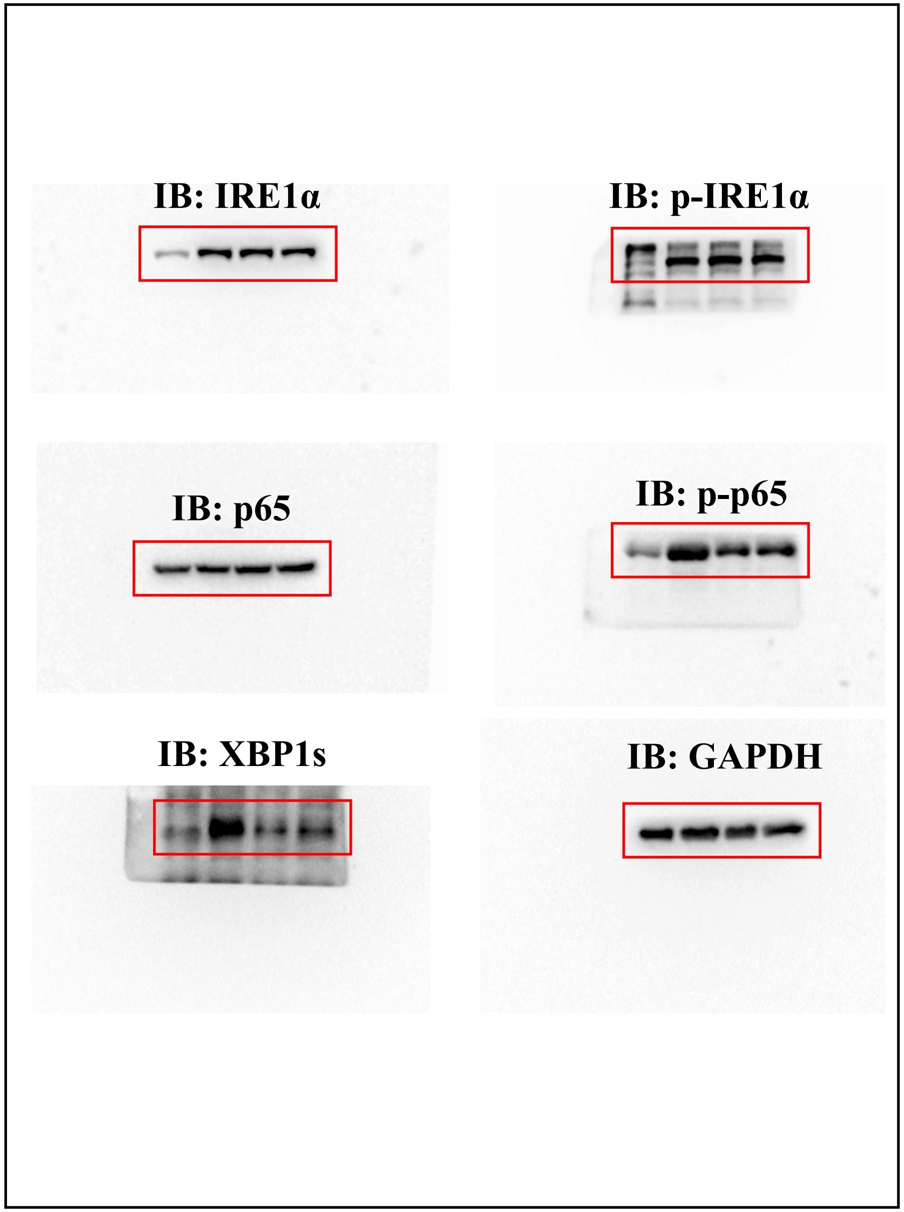

Fig. 4A- right

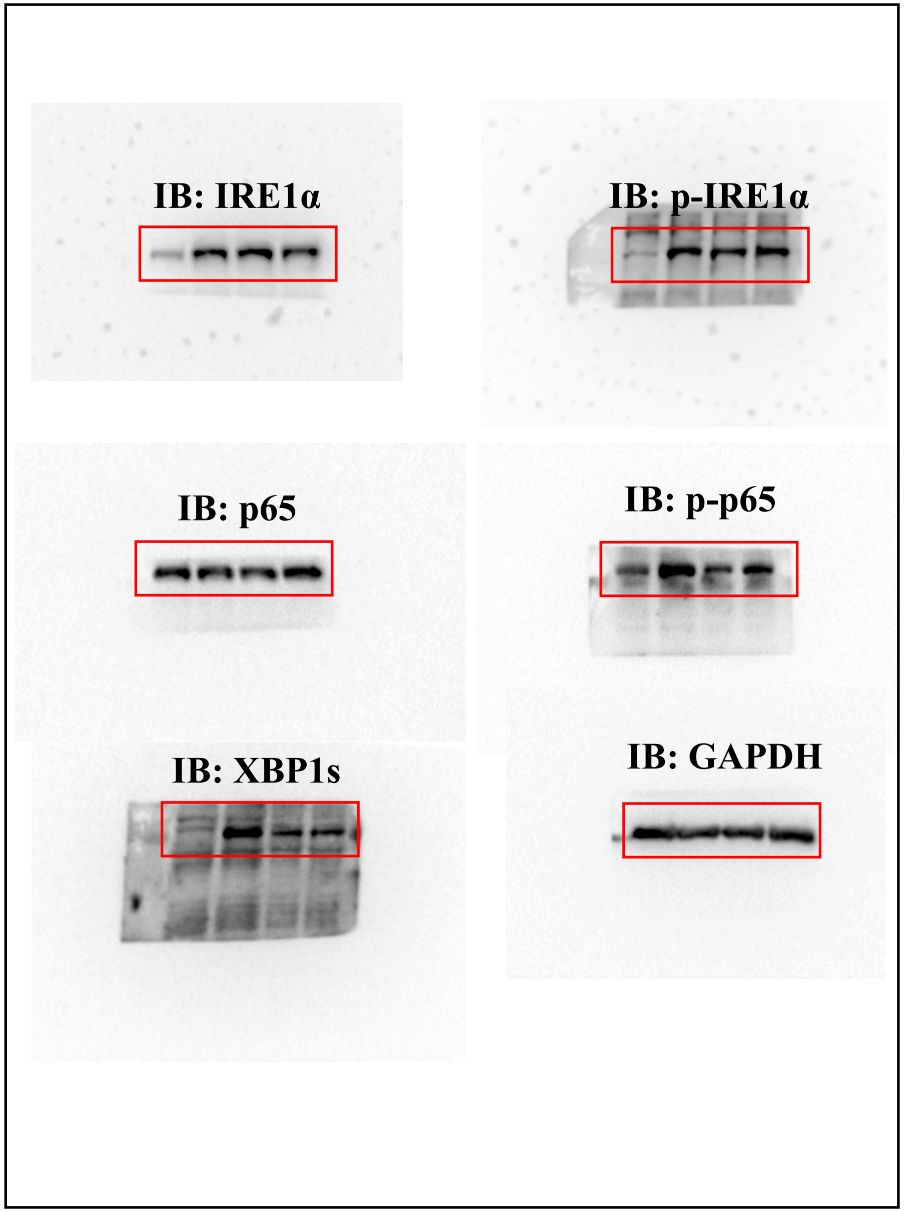

Fig. 4D- left

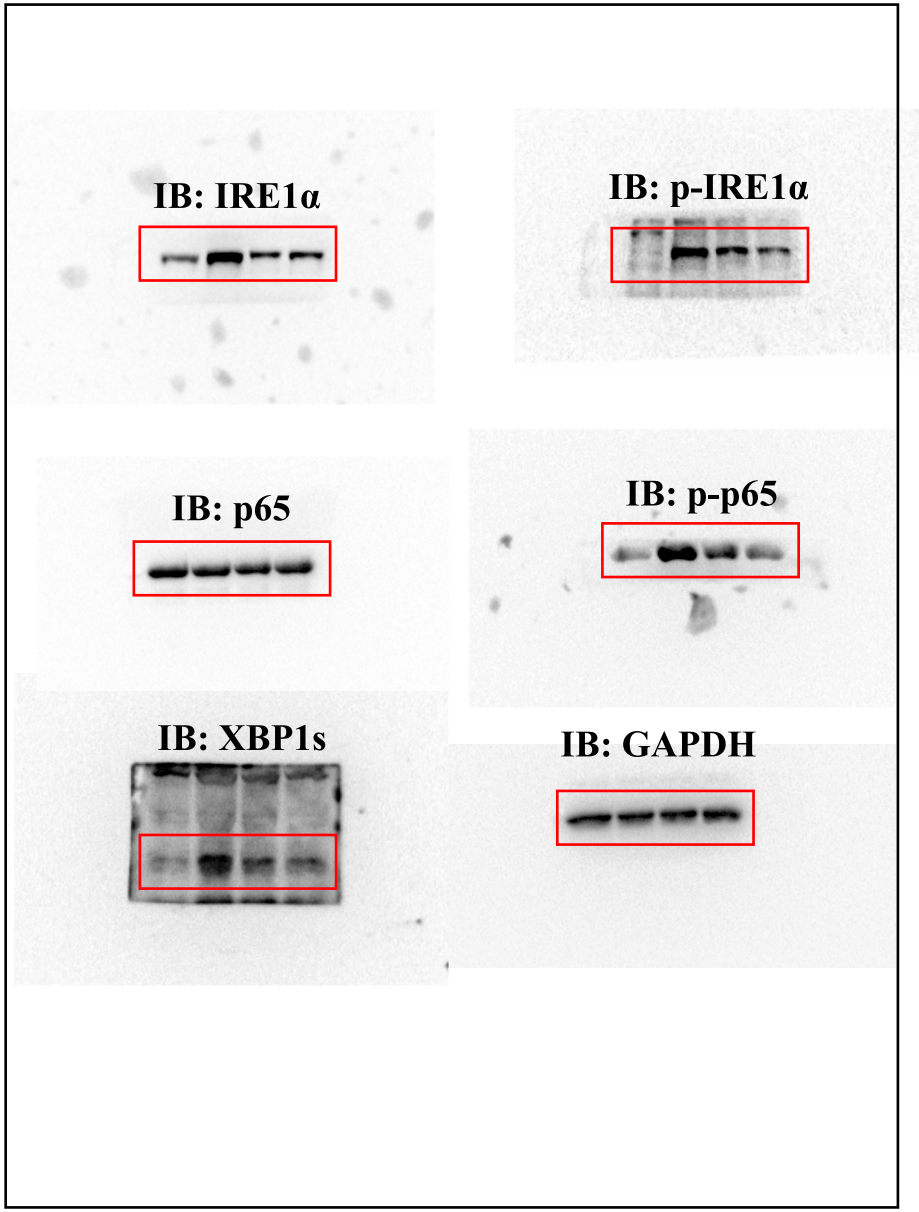

Fig. 4D- right

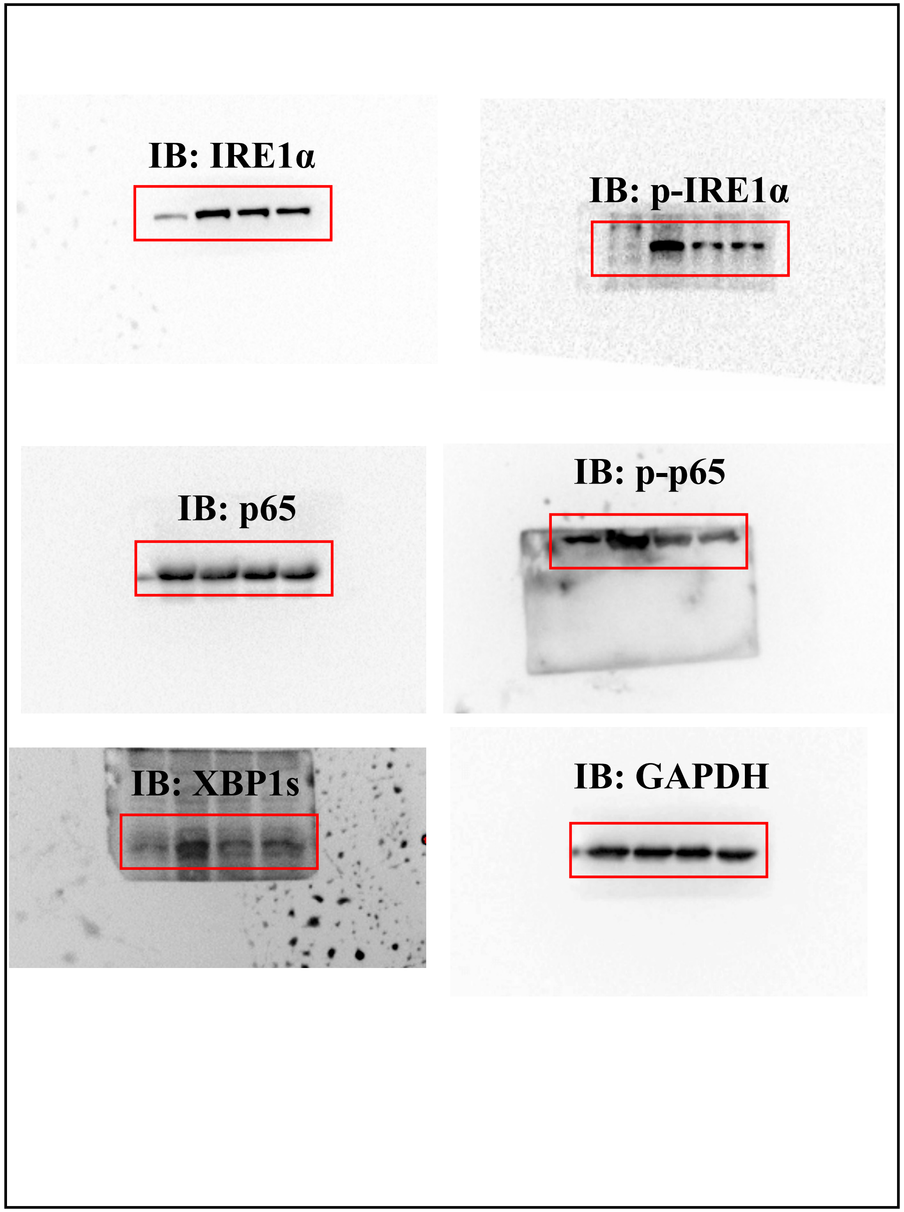

Fig. S2B

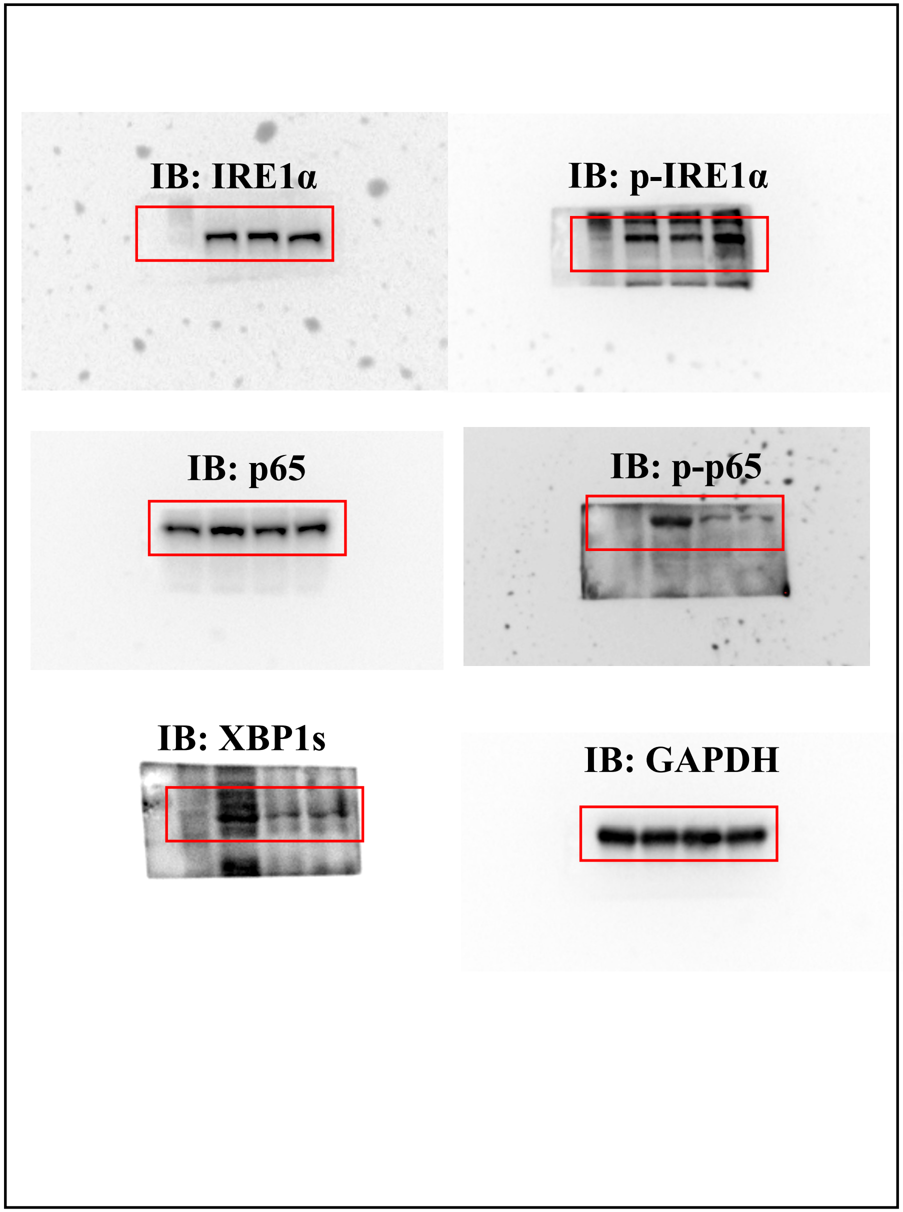

Fig. S3A

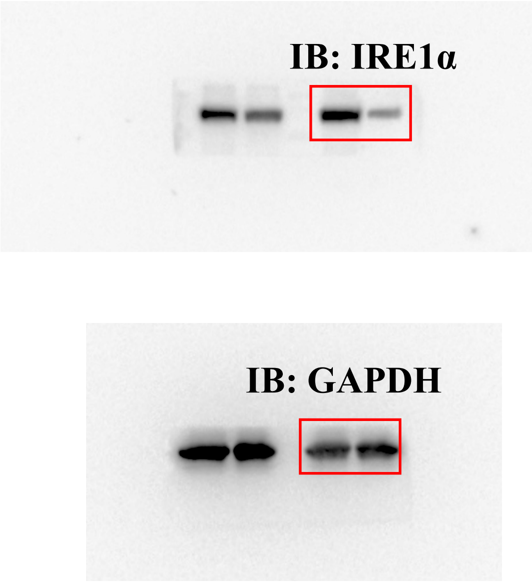

Fig. S3B

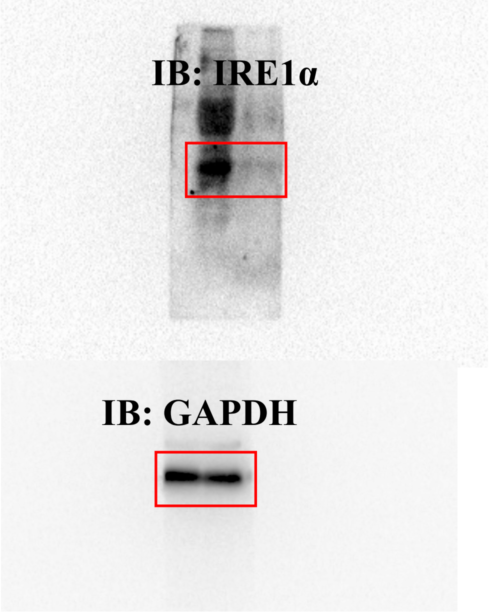

Fig. S4B- left

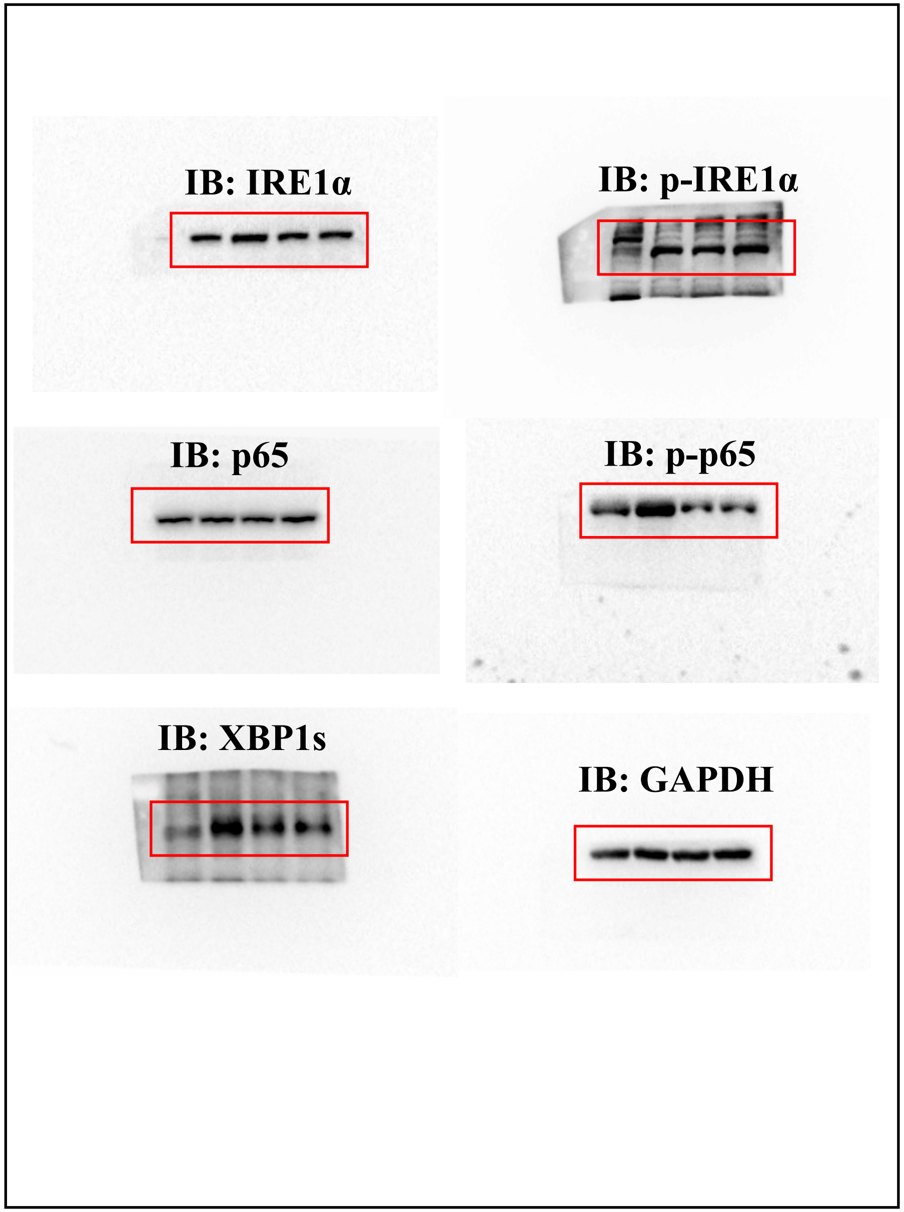

Fig. S4B- right

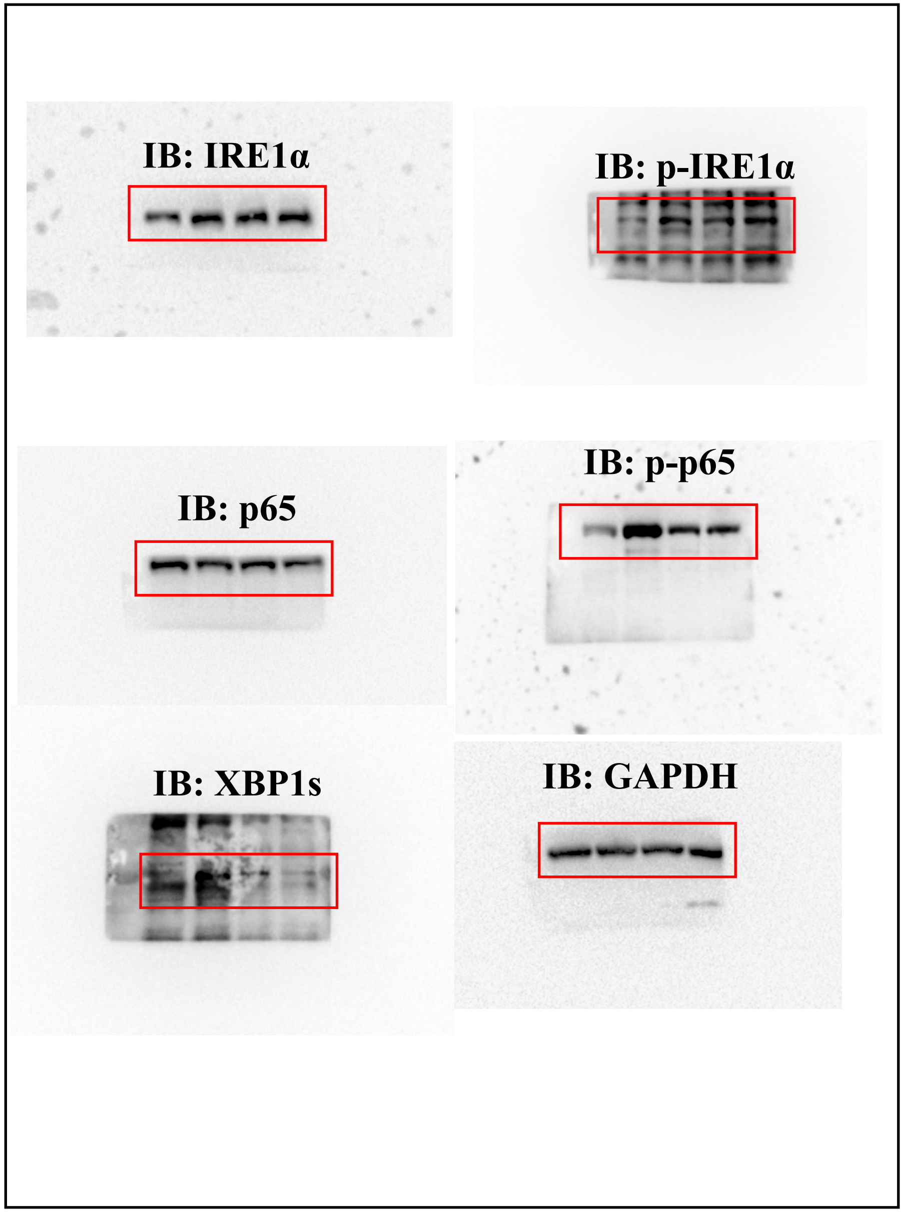

Fig. S4G- left

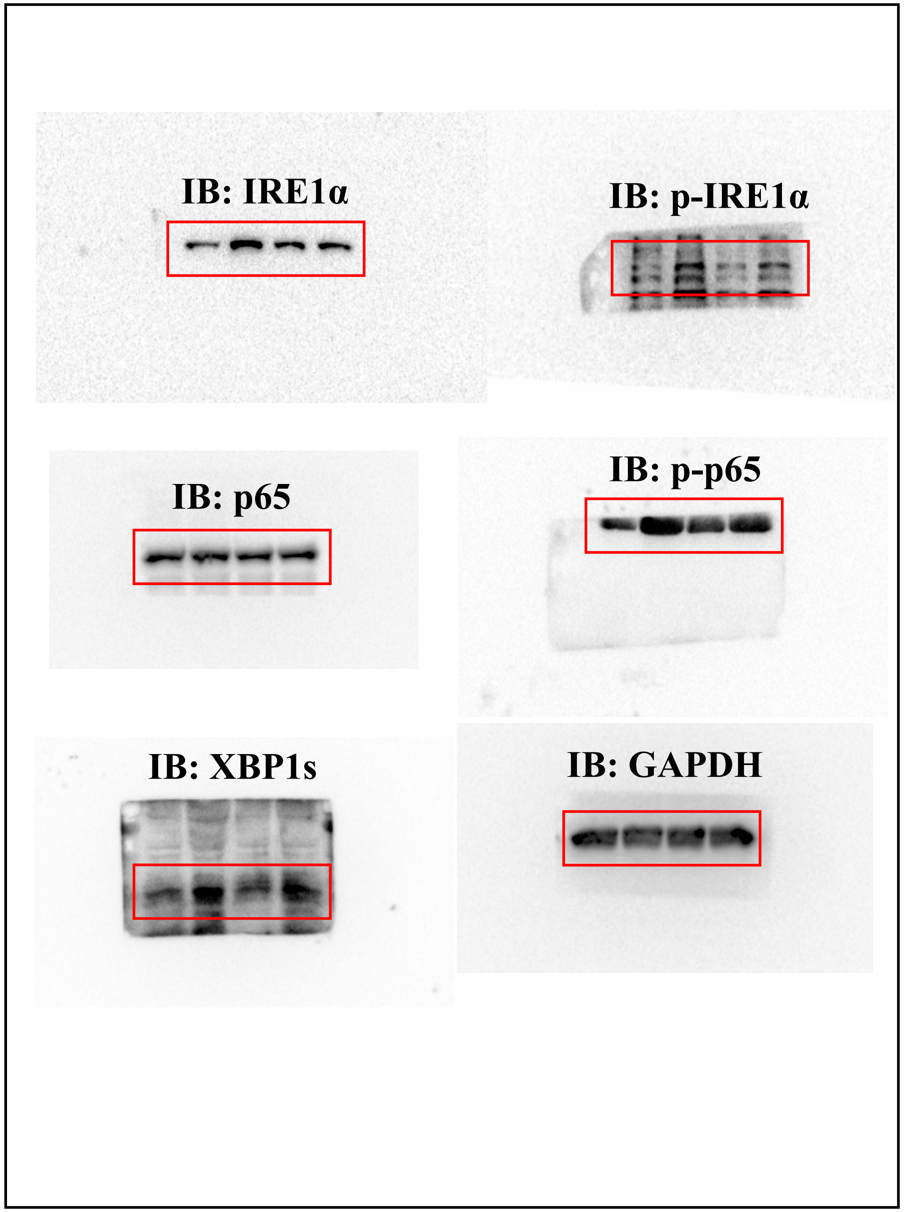

Fig. S4G- right

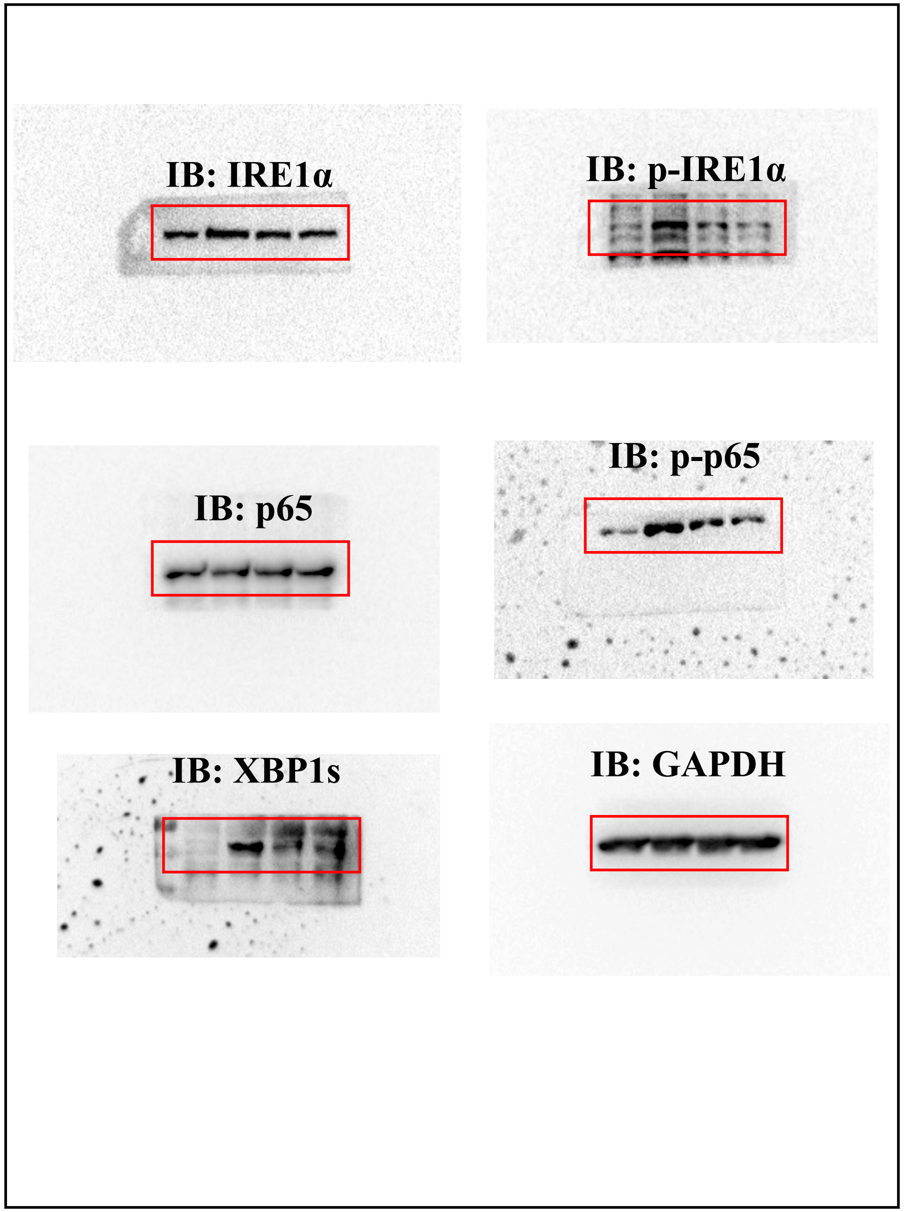

Fig. S4I- 1

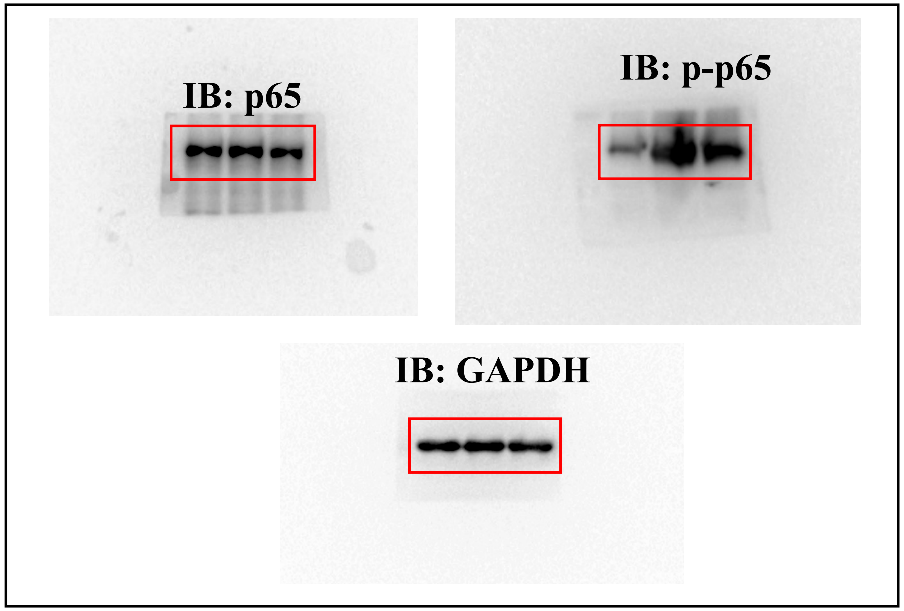

Fig. S4I- 2

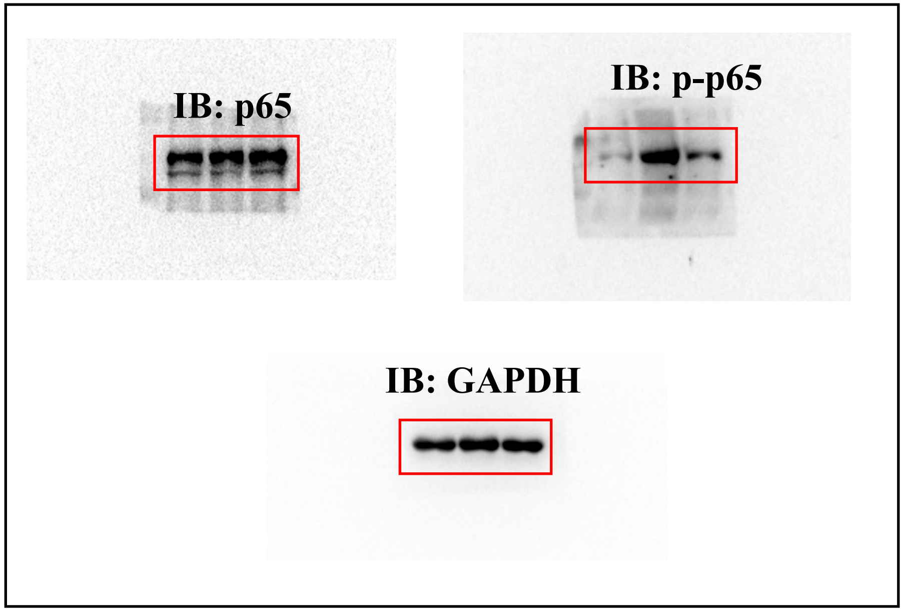

Fig. S4I- 3

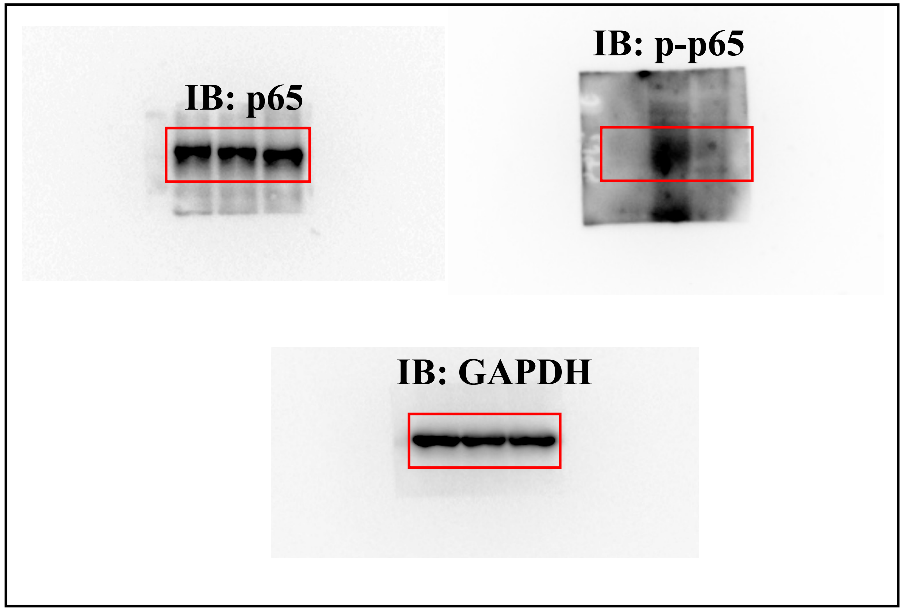

Fig. S4I- 4

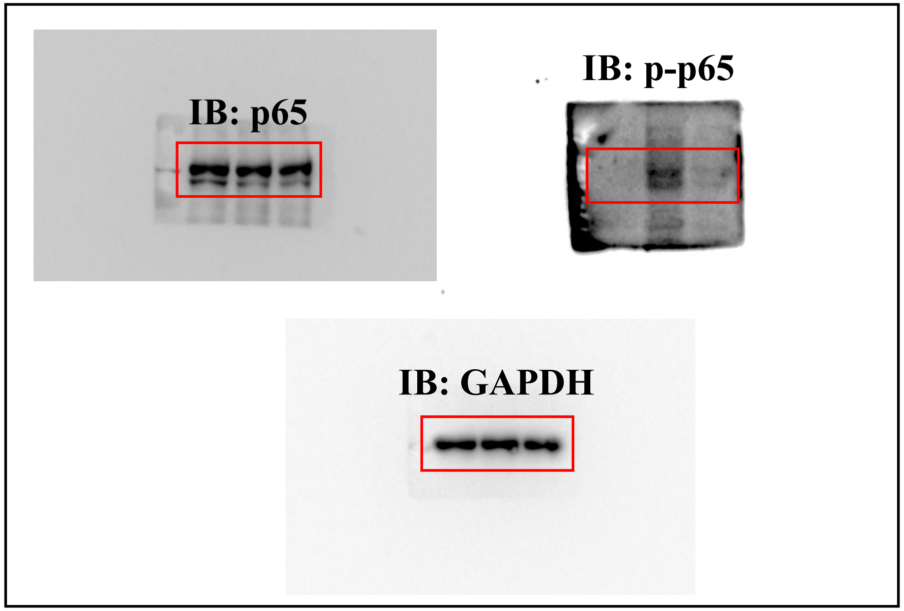

Fig. S5C

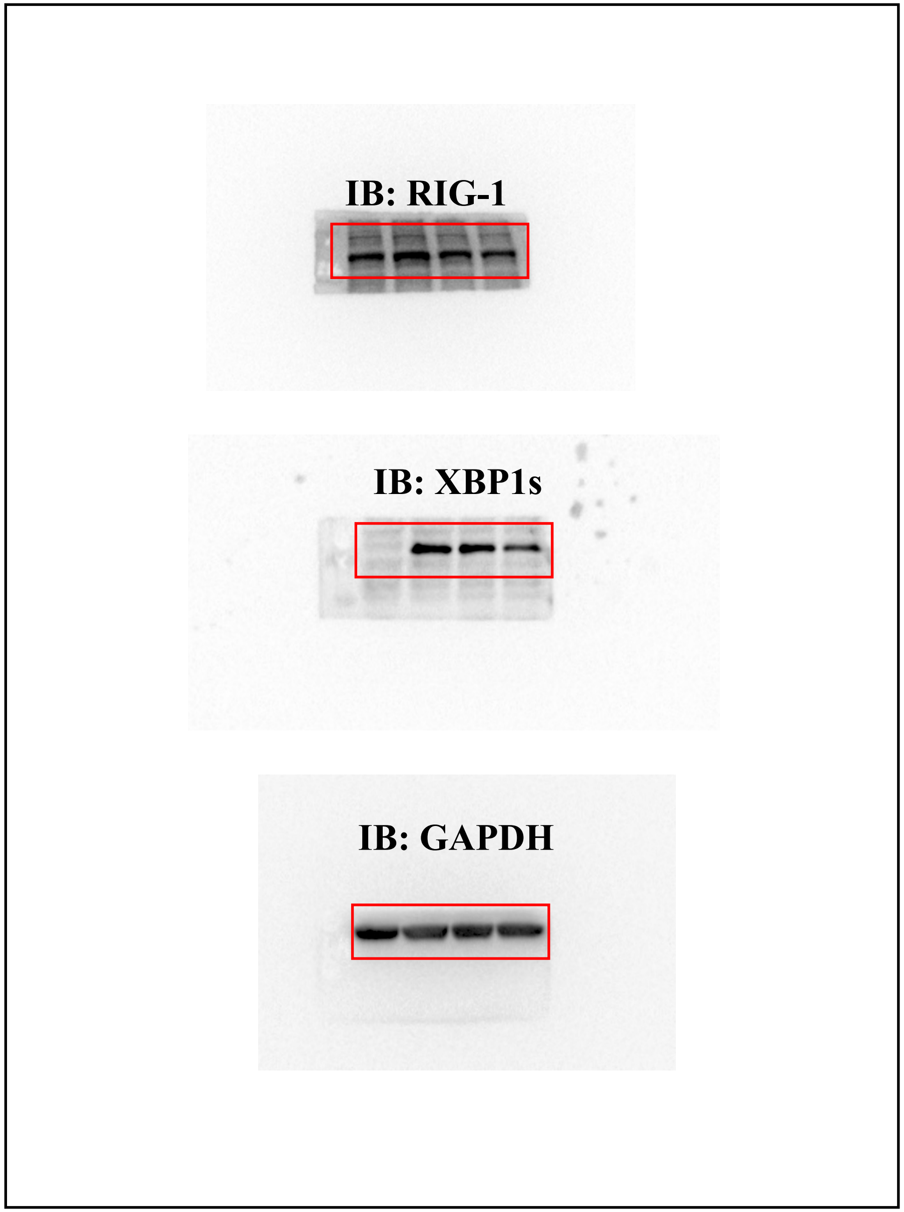

Fig. S5E

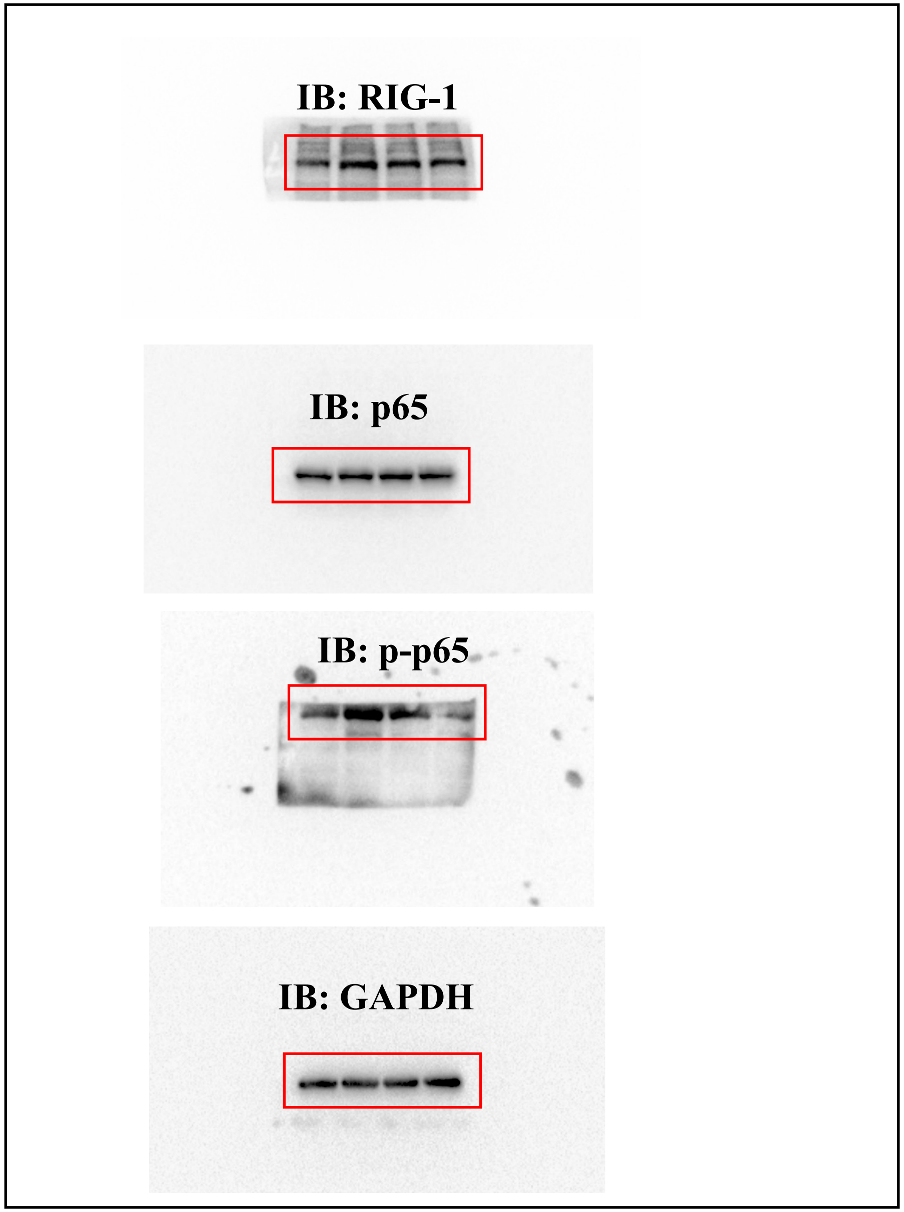

Fig. S8C- left

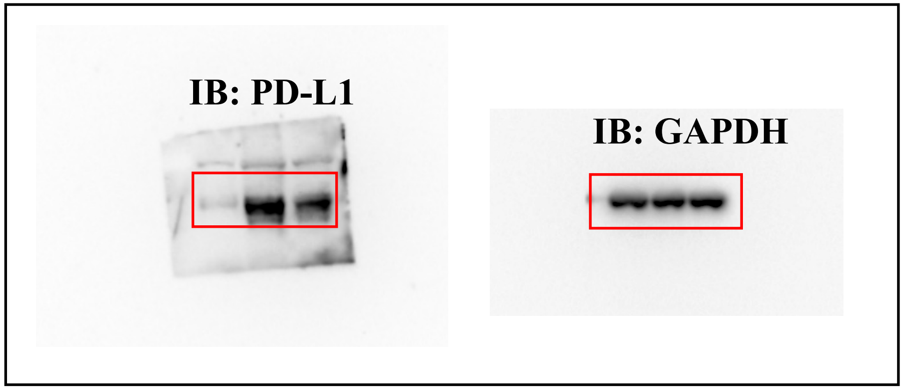

Fig. S8C- right

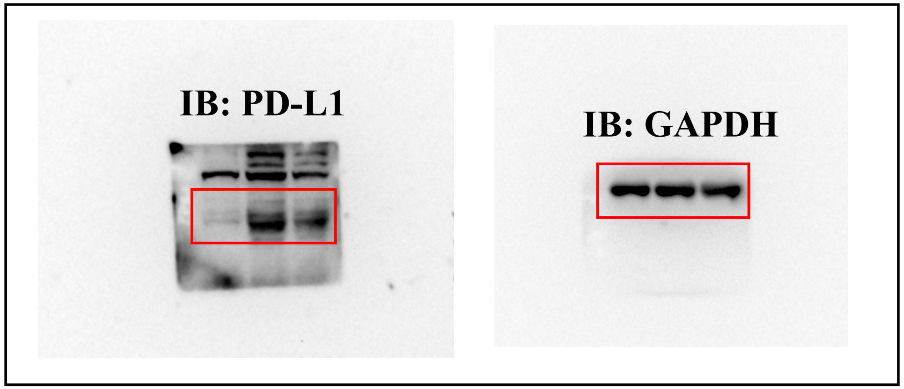

Fig. S8F- A2058 left

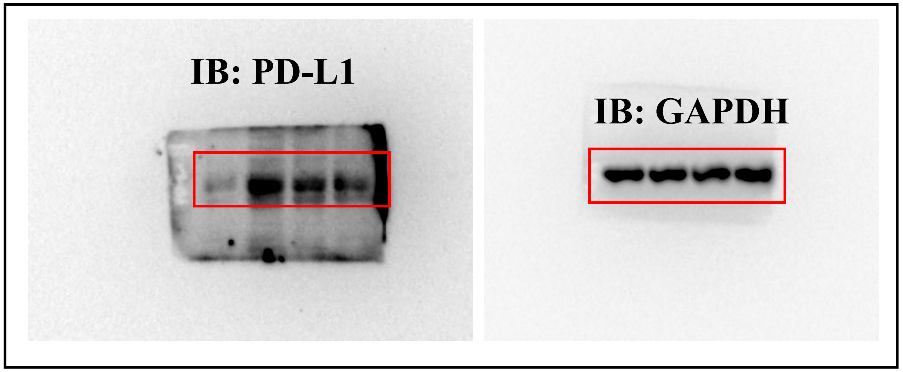

Fig. S8F- A2058 right

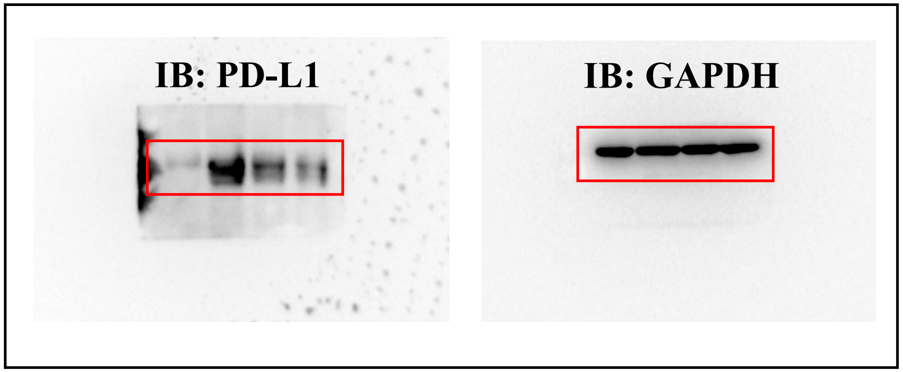

Fig. S8F- A375 left

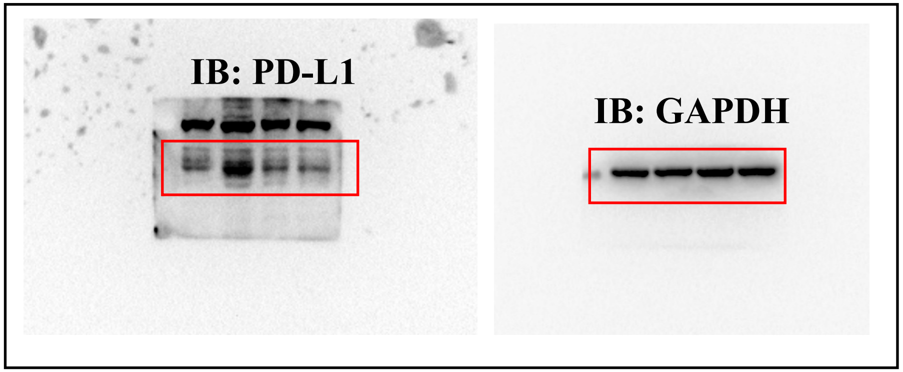

Fig. S8F- A375 right

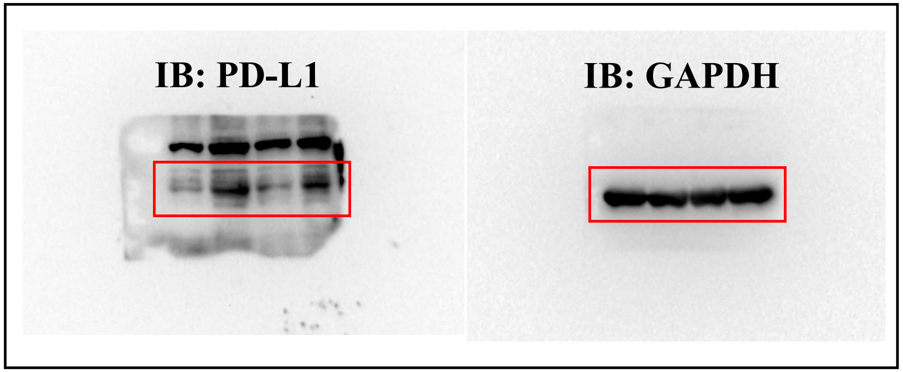

Fig. S8I- A2058 left

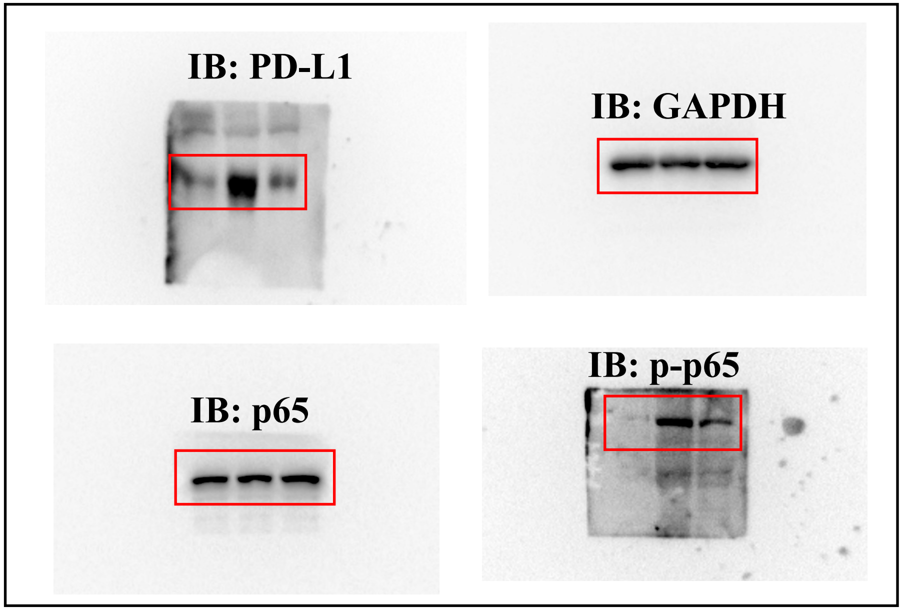

Fig. S8I- A2058 right

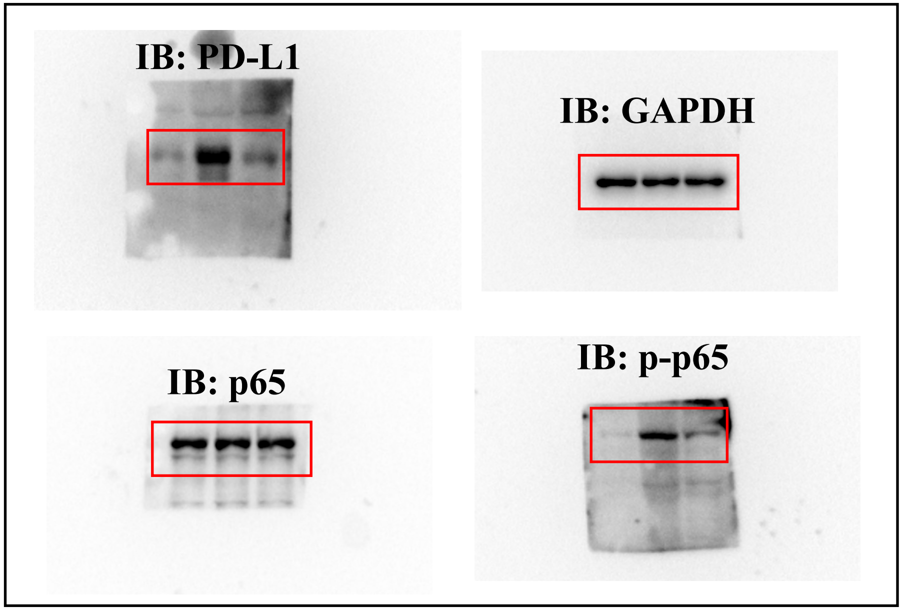

Fig. S8I- A375 left

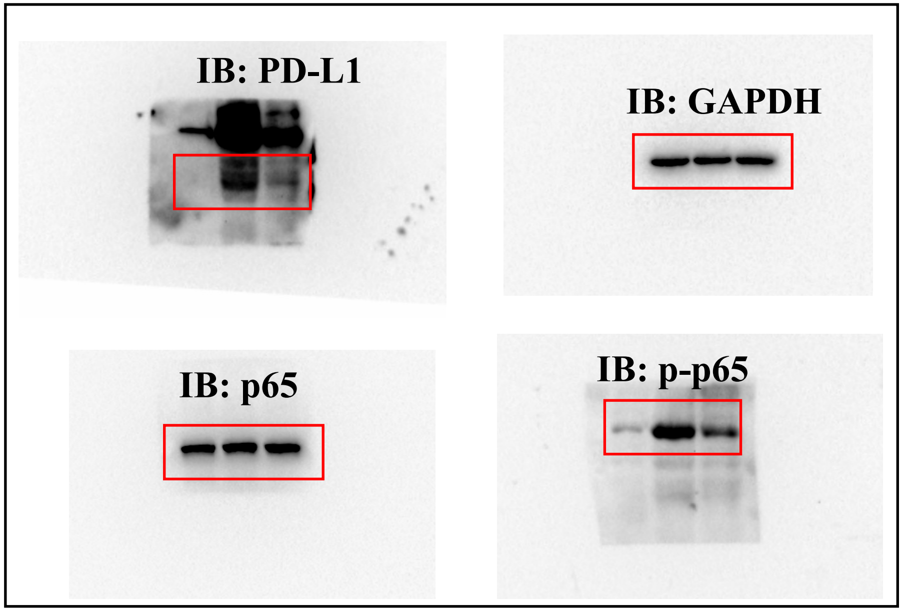

Fig. S8I- A375 right

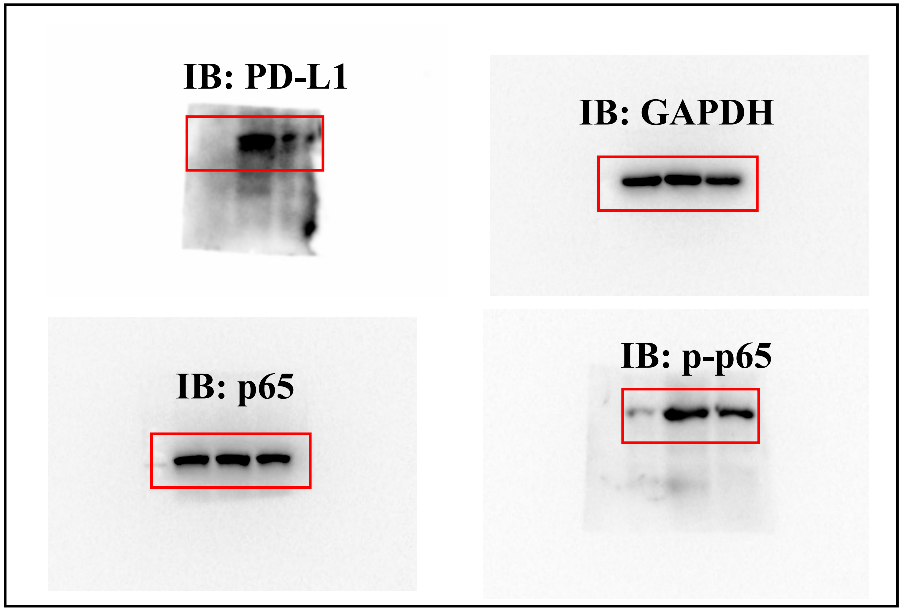

Supplement: Supplementary file 4 — Additional file 4. Original and uncropped films of Western blots. [file 12964_2024_1470_MOESM4_ESM.pdf]
